# Unexpected diversity of flower-associated *Aureobasidium* reveals sixteen novel species from northern Thailand

**DOI:** 10.3897/imafungus.17.188009

**Published:** 2026-08-03

**Authors:** Chanokned Senwanna, Pratthana Kodchasee, Chirayut Kathongthung, Milan C. Samarakoon, Pannida Khunnamwong, Jaturong Kumla, Nakarin Suwannarach

**Affiliations:** 1 Office of Research Administration, Chiang Mai University, Chiang Mai 50200, Thailand Biodiversity Center Kasetsart University (BDCKU) Bangkok Thailand; 2 Department of Biology, Faculty of Science, Chiang Mai University, Chiang Mai 50200, Thailand Department of Microbiology, Faculty of Science, Kasetsart University Bangkok Thailand; 3 Center of Excellence in Microbial Diversity and Sustainable Utilization, Chiang Mai University, Chiang Mai 50200, Thailand Center of Excellence in Microbial Diversity and Sustainable Utilization, Chiang Mai University Chiang Mai Thailand; 4 Department of Entomology and Plant Pathology, Faculty of Agriculture, Chiang Mai University, Chiang Mai 50200, Thailand Department of Biology, Faculty of Science, Chiang Mai University Chiang Mai Thailand; 5 Department of Microbiology, Faculty of Science, Kasetsart University, Bangkok 10900, Thailand Office of Research Administration, Chiang Mai University Chiang Mai Thailand; 6 Biodiversity Center Kasetsart University (BDCKU), Bangkok 10900, Thailand Department of Entomology and Plant Pathology, Faculty of Agriculture, Chiang Mai University Chiang Mai Thailand

**Keywords:** *
Aureobasidium
*, flower-associated fungi, multilocus phylogeny, taxonomy, tropical area, yeast-like fungi

## Abstract

*Aureobasidium* species are widely distributed fungi commonly associated with diverse ecological niches, including floral environments. However, their diversity in tropical regions, particularly in flower-associated habitats, remains poorly explored. During a survey of fungi associated with flowers from diverse plant hosts in northern Thailand in 2024, a total of 54 *Aureobasidium* strains were isolated from 31 flower species. Based on a polyphasic approach integrating multilocus phylogenetic analyses, morphological and physiological characteristics, and growth temperature profiles, these strains were identified as 16 novel species (*Aureobasidium anthicola***sp. nov**., *A. bougainvilleae***sp. nov**., *A. catharanthi***sp. nov**., *A. chiangmaiense***sp. nov**., *A. florale***sp. nov**., *A. floricola***sp. nov**., *A. florigenum***sp. nov**., *A. kwanphayaoense***sp. nov**., *A. lannaense***sp. nov**., *A. oroxyli***sp. nov**., *A. phayaoense***sp. nov**., *A. plumeriae***sp. nov**., *A. pollinicola***sp. nov**., *A. saisamorniae***sp. nov**., *A. savitreeae***sp. nov**., and *A. siamense***sp. nov**.) and five previously known species (*A. albui*, *A. castaneae*, *A. melanogenum*, *A. musti*, and *A. tremulum*). Species within the *A. melanogenum* and *A. thailandense* groups were delimited as novel species based on the genealogical concordance phylogenetic species recognition (GCPSR) concept, supported by pairwise homoplasy index tests and physiological traits. Carbon assimilation profiles represent useful phenotypic characteristics for distinguishing closely related species. Moreover, these are the first geographic records of *A. albui*, *A. castaneae*, *A. musti*, and *A. tremulum* in Thailand. Full descriptions, color photographs, illustrations, and a phylogenetic tree showing the phylogenetic positions of the *Aureobasidium* species obtained in this study are provided. This study highlights floral habitats as important and previously underexplored ecological niches for *Aureobasidium* in tropical ecosystems, providing new insights into its diversity, ecology, and distribution.

## Introduction

The family *Saccotheciaceae (Dothideales)* was established by Bonorden (1864) to accommodate *Saccothecium*. To date, 11 genera are included in this family, namely *Aureobasidium*, *Banningia*, *Columnosphaeria*, *Kabatiella*, *Moringomyces*, *Paivomyces*, *Pseudoselenophoma*, *Pseudoseptoria*, *Pseudosydowia*, *Saccothecium*, and *Selenophoma* (Hyde et al. 2024; Nascimento et al. 2025), with members occurring as saprophytes, endophytes, and pathogens in both extreme and benign environments, on various substrates, and on human skin (Hongsanan et al. 2020; Samaddar and Sharma 2023; da Silva et al. 2024; Jumbam et al. 2025). Among them, species of *Aureobasidium* are ascomycetous black yeast-like fungi that occupy a wide range of environments and substrates, including air, human skin, water, mangrove ecosystems, and organic and inorganic outdoor materials (i.e., food, house dust, phylloplanes, rocks, soil, and wood), playing roles as saprophytes, endophytes, and pathogens (Gostinčar et al. 2014; van Nieuwenhuijzen 2014; Li et al. 2015; Bennamoun et al. 2016; Nasr et al. 2018; Di Francesco et al. 2023; da Silva et al. 2024; Czachura and Piątek 2025; Jumbam et al. 2025). In addition, species of the genus are recognized as polyextremotolerant fungi that can survive under various extreme conditions, such as hypersaline water, glacial ice, deserts, frozen or salt-preserved food, and radiation-polluted areas (Gostinčar et al. 2014; Černoša et al. 2021, 2025; Zajc et al. 2022; Xiao et al. 2024). *Aureobasidium* species not only possess the ability to synthesize a variety of enzymes and natural products with potential applications in biotechnology and other industrial fields but are also used to promote plant growth and as biocontrol agents against various plant pathogens, such as *Alternaria alternata*, *Botrytis cinerea*, *Colletotrichum acutatum*, *Fusarium culmorum*, *Monilinia laxa*, *Penicillium digitatum*, *P. expansum*, and *Pseudomonas syringae* pv. actinidiae (Wachowska and Głowacka 2014; Spadaro and Droby 2016; Di Francesco et al. 2017, 2023; Gawai 2018; Prasongsuk et al. 2018; Agirman and Erten 2020; Yalage Don et al. 2020; Galli et al. 2021; Cignola et al. 2023; Iqbal et al. 2023; Cornish et al. 2025). Furthermore, *A. pullulans* is recognized for its ability to degrade aromatic pollutants and plastics (Webb et al. 2000; Wang et al. 2022a; Rensink et al. 2024).

Members of the genus *Aureobasidium* are morphologically classified as dimorphic fungi, exhibiting both yeast and mycelial forms that vary depending on the species and environmental conditions (Gaur et al. 2010; van Nieuwenhuijzen 2014). They are characterized by dark, slimy colonies, micronematous conidiogenous cells, and aseptate ellipsoidal conidia; primary conidia produce yeast-like budding secondary conidia (Prabhugaonkar and Pratibha 2018). Currently, 62 species are accepted in the genus *Aureobasidium*, of which 11 lack sequence data and have not been phylogenetically confirmed (Czachura and Piątek 2025; Jumbam et al. 2025; MycoBank 2026; Tan et al. 2026a, 2026b). Among these, *A. melanogenum* and *A. pullulans* are common species with the widest distributions and most diverse habitats (Onetto et al. 2020; Wang et al. 2022a; Rensink et al. 2024). Traditionally, species delimitation of *Aureobasidium* was based primarily on morphological characteristics, such as colony appearance, pigmentation, and conidial morphology; however, these traits often show high phenotypic variability and overlap among species, leading to taxonomic ambiguity (Dennis and Buhagiar 1973; Hermanides-Nijhof 1977; Kocková-Kratochvílová et al. 1980). Physiological and biochemical characteristics, including growth temperature profiles and carbon and nitrogen assimilation profiles, are also used to support the identification and differentiation of *Aureobasidium* species (de Hoog and Yurlova 1994; Yurlova and de Hoog 1997; Nasr et al. 2018; Jumbam et al. 2025). Subsequently, with the advent of molecular techniques, particularly DNA sequencing, species identification has become more reliable. Early studies primarily utilized ribosomal DNA regions, such as the internal transcribed spacer (ITS), for species-level identification (Yurlova et al. 1995, 1996; de Hoog et al. 1999; Prasongsuk et al. 2005; Peterson et al. 2013). Nevertheless, because of the limited resolution among closely related taxa, multilocus phylogenetic approaches incorporating additional gene regions, such as the 28S large subunit (LSU), RNA polymerase II second-largest subunit (*rpb2*), translation elongation factor 1-alpha (*tef1*), and β-tubulin (*tub*) loci, have been increasingly applied to resolve species boundaries and elucidate evolutionary relationships within the genus (Zalar et al. 2008; Humphries et al. 2017; Prasongsuk et al. 2018; da Silva et al. 2024; Jumbam et al. 2025). Additionally, phylogenetic analyses based on whole-genome sequences have demonstrated that the four *A. pullulans* varieties represent distinct species, namely *A. melanogenum*, *A. namibiae*, *A. pullulans*, and *A. subglaciale* (Gostinčar et al. 2014). Recent advances in species delimitation methods have improved taxonomic resolution within *Aureobasidium*. Comparative studies of genealogical concordance phylogenetic species recognition (GCPSR), coalescent-based species recognition (CBSR), and distance-based species recognition (DBSR) have shown that GCPSR provides the most accurate and consistent approach for defining species boundaries within the genus (Jumbam et al. 2025). This approach can be supported by physiological characterization, particularly carbon assimilation and fermentation tests, to improve the resolution of species delineation (Jumbam et al. 2025).

Several studies have established flowers as important habitats for fungal diversity. Floral structures provide unique microenvironments with abundant nutrients, transient moisture, and frequent biotic interactions, creating favorable conditions for fungal colonization (Vannette et al. 2021; Larrouy et al. 2023; Ruraż et al. 2023; Shi et al. 2024). A wide range of fungi, including yeasts, endophytes, epiphytes, and latent pathogens, have been reported from floral tissues, with common genera including *Alternaria*, *Aspergillus*, *Aureobasidium*, *Candida*, *Cladosporium*, *Colletotrichum*, *Fusarium*, *Metschnikowia*, and *Penicillium* (Zhou et al. 2014; Moreira et al. 2021; Zimowska et al. 2021; Niu et al. 2022; Di Francesco et al. 2023; Kanpiengjai et al. 2023; Agarbati et al. 2024; Shi et al. 2024). *Aureobasidium* species, particularly *A. pullulans*, have been increasingly recognized as common inhabitants of floral environments across diverse geographic regions and host plants. For instance, *A. pullulans* has been reported from a variety of floral sources, including tiger lily (*Lilium* sp.), monkshood (*Aconitum carmichaeli*), wild daisy (*Chrysanthemum boreale*), and eastern daisy fleabane (*Erigeron anuus*) in South Korea (Anastassiadis et al. 2003; Kim et al. 2015; Kim and Kim 2015; Kim et al. 2018; Choi et al. 2019); pink node flowers (*Caesulia axillaris*) in India (Choudhury et al. 2011); and the nectar of kidneyvetch (*Anthyllis vulneraria*), snapdragons (*Antirrhinum australe*), willow-leaved foxglove (*Digitalis obscura*), and blue broom (*Erinacea anthyllis*) in Spain (Pozo et al. 2011). It has also been reported from hummingbird flowers (*Epilobium canum*) in the USA (Morris et al. 2020). In Brazil, *A. pullulans* and *A. thailandense* have been isolated from inflorescences of bromeliad plants (Félix et al. 2021). The successful colonization of floral environments by *Aureobasidium* can be attributed to several adaptive features, including its ability to withstand osmotic stress in high-sugar nectar, resistance to UV radiation and desiccation through melanin production, and efficient surface colonization via extracellular polysaccharides (Gostinčar et al. 2014; Zalar et al. 2008). Previous studies found that *A. pullulans* effectively colonizes floral tissues, particularly stigmas, and suppresses fire blight through the competitive exclusion of the pathogen (Temple et al. 2020; Zeng et al. 2023). Furthermore, the dispersal capacity of *Aureobasidium* in floral environments, influenced by factors such as wind, rainfall, microhabitat heterogeneity, resource availability, and biotic interactions (e.g., pollinators), may contribute to shaping its distribution and diversification (Choi et al. 2019; Pozo et al. 2011; Di Francesco et al. 2023; Lignon et al. 2025).

Thailand is noted to be one of the world’s most biodiverse countries, with an estimated 15,000 plant species (approximately 8% of the global total), providing a foundation for diverse floral habitats that may support unique fungal and microbial communities (Donnelly 2024; Office of Natural Resources and Environmental Policy and Planning 2024; Saensouk et al. 2025). Nevertheless, research on flower-associated fungi in Thailand remains limited. Additionally, the diversity of *Aureobasidium* species within the country remains poorly investigated, with only a few reports available (Punnapayak et al. 2003; Peterson et al. 2013). To date, *A. melanogenum*, *A. pullulans*, and *A. thailandense* are among the few *Aureobasidium* species reported from various substrates in Thailand, and they have been investigated for their physiological characteristics and extracellular polysaccharide production (Sudhadham 2001; Punnapayak et al. 2003; Zalar et al. 2008; Peterson et al. 2013; Prasongsuk et al. 2018). During a survey of fungi associated with flowers in northern Thailand in 2024, a total of 54 *Aureobasidium* strains were isolated. Therefore, this study aimed to characterize and identify these *Aureobasidium* strains using morphological characteristics, growth temperature profiles, and multilocus phylogenetic analyses of the ITS, LSU, *rpb2*, *tef1*, and *tub* loci. Moreover, following Jumbam et al. (2025), multiple species delimitation approaches, including GCPSR and CBSR analyses, together with physiological characteristics, were employed to delimit novel species within the *A. melanogenum* and *A. thailandense* species groups.

## Materials and methods

### Flower collection and fungal isolation

**Figure 1.**
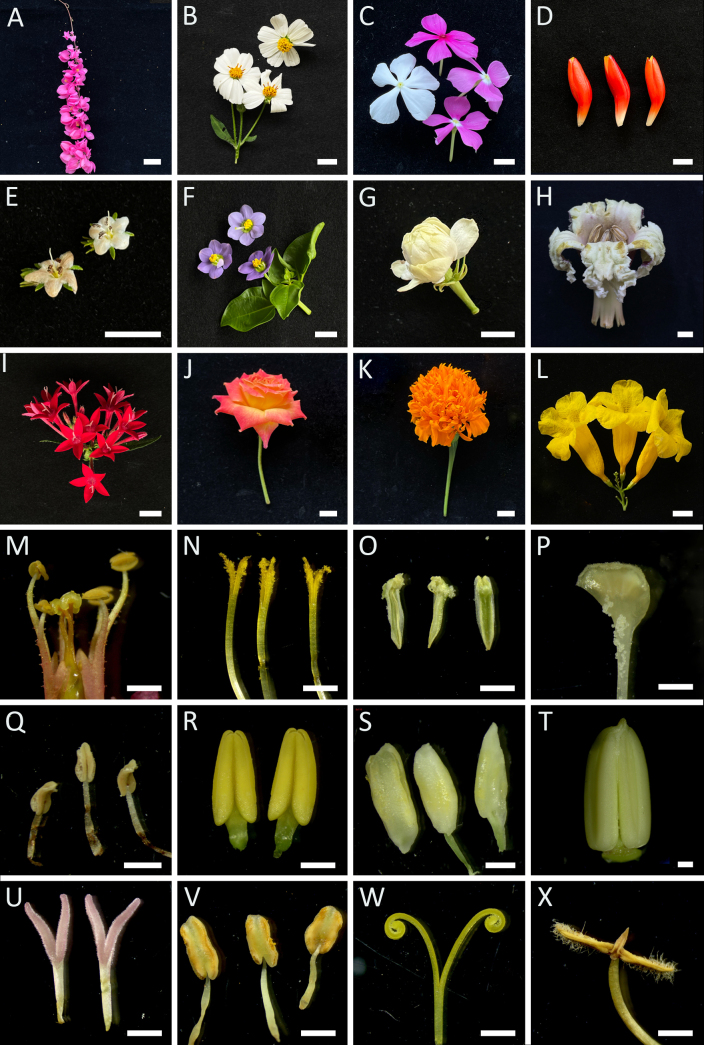
Some flower samples collected from northern Thailand (**A–L**) and their internal parts (**M–X**). **A, M***Antigonon leptopus*; **B, N***Bidens pilosa*; **C, O***Catharanthus roseus*; **D, P***Cheilocostus speciosus*; **E, Q***Ehretia microphylla*; **F, R***Exacum affine*; **G, S***Jasminum sambac*; **H, T***Oroxylum indicum*; **I, U***Pentas lanceolata*; **J, V***Rosa* sp.; **K, W***Tagetes erecta*; **L, X***Tecoma stans*. Scale bars: 1 cm (**A–L**); 1 mm (**M–X**).

Healthy flowers from 31 plant species (Fig. 1) were randomly collected from living plants in Mueang Chiang Mai and Phrao Districts (Chiang Mai Province) and Mueang Phayao District (Phayao Province), northern Thailand, from July to September 2024. Flowers were placed in sterile plastic bags, stored at 4 °C, and processed for fungal isolation within 24 h of collection.

The excised internal parts of flowers, including the carpel, stamen, pollen, and nectary, were removed at random from each flower species using sterile forceps. The samples were immersed in 5 mL of sterile saline solution (0.85% w/v NaCl) for 15 min and then vortexed for 1 min. The solution was then spread on potato dextrose agar (PDA; Condalab, Laboratorios Conda S.A., Spain) supplemented with 50 mg/L chloramphenicol. After incubation for 72 h at 25 °C in the dark, colonies with a characteristic shiny appearance were selected and transferred to fresh PDA plates for further incubation at 25 °C. Each pure fungal strain was preserved for short-term storage on PDA slants at 4 °C and for long-term storage in 20% glycerol at –80 °C at the Sustainable Development of Biological Resources Culture Collection (SDBR-CMU), Faculty of Science, Chiang Mai University, Chiang Mai, Thailand. Holotype specimens of the new species were deposited as dried specimens in the Chiang Mai University Biology Department’s Herbarium (CMUB), Chiang Mai University, Chiang Mai Province, Thailand, whereas ex-type living cultures were permanently maintained in a metabolically inactive state at SDBR-CMU, the Thailand Bioresource Research Center Culture Collection (TBRC), Pathum Thani Province, Thailand, and the Guizhou Medical University Culture Collection (GMBCC), Guiyang, China. Nomenclatural and taxonomic information was deposited in the MycoBank database (http://www.mycobank.org/).

### Fungal identification

#### Morphological studies

Pure cultures were examined on PDA, malt extract agar (MEA; Gibco, Life Technologies Corporation, USA), and oatmeal agar (OA; Difco, Becton, Dickinson and Company, USA). Colony diameters and culture characteristics were recorded after two weeks of incubation at 25 °C in the dark. Colony colors were recorded according to the *Methuen Handbook of Colour* (Kornerup and Wanscher 1967). In addition, the cardinal growth temperatures of the fungal strains were determined on MEA at 4, 10, 25, 30, and 37 °C after two weeks of incubation in the dark (Jumbam et al. 2025). A Nikon DS-Ri2 camera fitted to a Nikon ECLIPSE Ni compound microscope (Tokyo, Japan) was used to examine and capture microscopic features, including hyphae, conidiophores, conidiogenous cells, conidia, and chlamydospores. The fungal structures were measured using the Tarosoft Image Framework program (v. 0.9.0.7). Adobe Photoshop version 22.4.2 (Adobe Systems, USA) was employed to create the photographic plates.

#### DNA extractions, polymerase chain reaction, and sequencing

Genomic DNA was extracted from two-week-old *Aureobasidium* cultures of each strain using the OMEGA E.Z.N.A.® Forensic DNA Kit (Omega Bio-tek, Inc., USA), following the manufacturer’s protocol. The ITS, LSU, *rpb2*, *tef1*, and *tub* loci were amplified by polymerase chain reaction (PCR) using the ITS5/ITS4, LR0R/LR5, fRPB2-5F/fRPB2-7cR, EF1-728F/EF1-986R, and Bt2a/Bt2b primer pairs, respectively. PCR amplification and sequencing were performed according to previous studies (Senwanna et al. 2024, 2025; da Silva et al. 2024; Czachura and Piątek 2025; Kodchasee et al. 2026).

#### Sequence alignment and phylogenetic analyses

The closest matching taxa were determined using nucleotide BLAST searches conducted in GenBank (http://www.ncbi.nlm.nih.gov/). The alignments of each individual locus were compiled using representative sequences of the closest relatives of the strains, selected based on previous phylogenetic studies (da Silva et al. 2024; Czachura and Piątek 2025; Jumbam et al. 2025). Furthermore, the combined ITS, LSU, *rpb2*, *tef1*, and *tub* datasets were analyzed to confirm the phylogenetic placement of the new strains. The obtained alignments were deposited in TreeBase (https://www.treebase.org/) under submission number 32509 (Suppl. material 1). Phylogenetic analysis of the combined alignment was conducted using maximum likelihood (ML) in RAxML-HPC2 on XSEDE (v. 8.2.12) via the CIPRES web portal and Bayesian inference (BI) in MrBayes v. 3.2.6 (Ronquist et al. 2012).

In the ML tree, ML bootstrap support values (MLBS) were estimated under the GTRGAMMA nucleotide substitution model with 1,000 replicates. The best-fit models used for the BI analyses, which were determined based on the Akaike information criterion (AIC) using MrModeltest v. 2.3 (Nylander 2004), were SYM+I+G for ITS, GTR+I+G for LSU, *rpb2*, and *tub*, and GTR+G for *tef1*. Four simultaneous Markov chains were run for 6,000,000 generations, with trees sampled every 100 generations. The analysis was terminated when the average standard deviation of split frequencies fell below 0.01. The first 15,000 trees were discarded as burn-in, and the remaining 45,000 trees were used to calculate posterior probabilities (PP) in the majority-rule consensus tree. A phylogram generated from the ML analysis was visualized using FigTree v. 1.4.0 (Rambaut 2016) and edited using Adobe Illustrator version 25.2.3 and Adobe Photoshop version 22.4.2 (Adobe Systems, USA). The new nucleotide sequence data were deposited in GenBank.

#### Physiological characterization

The physiological characteristics of the newly identified *Aureobasidium* species obtained in this study were assessed according to the standardized methods described by Kurtzman et al. (2011), and the results of the growth tests were recorded after three weeks of incubation. Carbon and nitrogen assimilation tests were conducted in liquid media, whereas nitrogen assimilation tests were performed using starved inocula. Glucose fermentation was examined in a liquid medium using Durham fermentation tubes. Three replicates were conducted for each strain.

### Species delimitation analyses

#### Coalescent-based species recognition (CBSR)

CBSR analyses were conducted using the Poisson tree processes (PTP) (Zhang et al. 2013) and general mixed Yule-coalescent (GMYC) (Fujisawa and Barraclough 2013) models. The Bayesian implementation of the PTP model (bPTP) was conducted using the PTP web server (https://species.h-its.org/ptp/; accessed on 25 May 2026), applying both the maximum-likelihood solution (mlbPTP; Zhang et al. 2013) under a single Poisson distribution and the Bayesian solution (bPTP). The multi-rate PTP (mPTP; https://mptp.h-its.org/#/tree; accessed on 25 May 2026) was also performed (Kapli et al. 2017). This improved version of PTP does not require user-defined parameters and estimates support values for each delimited species using Markov chain Monte Carlo (MCMC). These values can be used to assess confidence in the maximum-likelihood delimitation. The maximum-likelihood tree obtained from the phylogenetic analysis was used as input for all PTP-based analyses. For GMYC analysis, the input file was generated using the BEAST v. 1.10.4 package (Suchard et al. 2018). The XML file was prepared in BEAUti v. 1.10.4 using the same alignment as that used in the phylogenetic analysis and was run in BEAST via the CIPRES Science Gateway. The resulting log file was examined in Tracer v. 1.7, and the tree file was summarized using TreeAnnotator v. 2.6.2. The resulting ultrametric tree was visualized in FigTree v. 1.4.0 and uploaded to the GMYC web server (https://species.h-its.org/gmyc/; accessed on 27 May 2026) using the single-threshold method. The resulting delimitation was then annotated on the phylogenetic tree for final presentation.

#### Genealogical concordance phylogenetic species recognition (GCPSR)

GCPSR analyses using the pairwise homoplasy index (PHI; Φw) were conducted in SplitsTree4 v. 4.17.1 (Huson and Bryant 2006) to assess the level of recombination among phylogenetically closely related species represented by type strains generated in this study within the *A. melanogenum* and *A. thailandense* species groups. The concatenated ITS, LSU, *rpb2*, *tef1*, and *tub* datasets of these related species were used for the PHI test and related analyses. The relationships between the newly generated strains and other closely related species were visualized by constructing split graphs using the LogDet transformation and split decomposition options. PHI results equal to or greater than 0.05 (Φw ≥ 0.05) indicated no significant recombination within the dataset, suggesting that related species within the group were genetically distinct. In contrast, PHI results below 0.05 (Φw < 0.05) indicated significant recombination within the dataset.

## Results

### Fungal isolation and sequence similarity

A total of 54 *Aureobasidium* strains were isolated from 31 flower species collected in northern Thailand (Suppl. material 2). The highest numbers of strains were recovered from *Catharanthus roseus* and *Ocimum tenuiflorum* (four strains each), followed by *Jatropha integerrima*, *Pentas lanceolata*, *Plumeria obtusa*, *Senna spectabilis*, and *Tectona grandis* (three strains each). DNA sequences (ITS, LSU, *rpb2*, *tef1*, and *tub*) obtained from each strain were deposited in GenBank (Suppl. material 3). Based on morphological characteristics and molecular data, all strains were confirmed to belong to the genus *Aureobasidium*. Based on sequence similarity, 12 strains were identified as five previously known species, including *A. albui* (one strain), *A. castaneae* (one strain), *A. melanogenum* (two strains), *A. musti* (two strains), and *A. tremulum* (six strains). This represents the first report of *A. albui*, *A. castaneae*, *A. musti*, and *A. tremulum* from Thailand. The remaining 42 strains potentially represented novel species. Therefore, a multilocus phylogenetic approach was employed to confirm their phylogenetic placement in combination with multiple species-recognition criteria.

### Phylogeny

**Figure 2.**
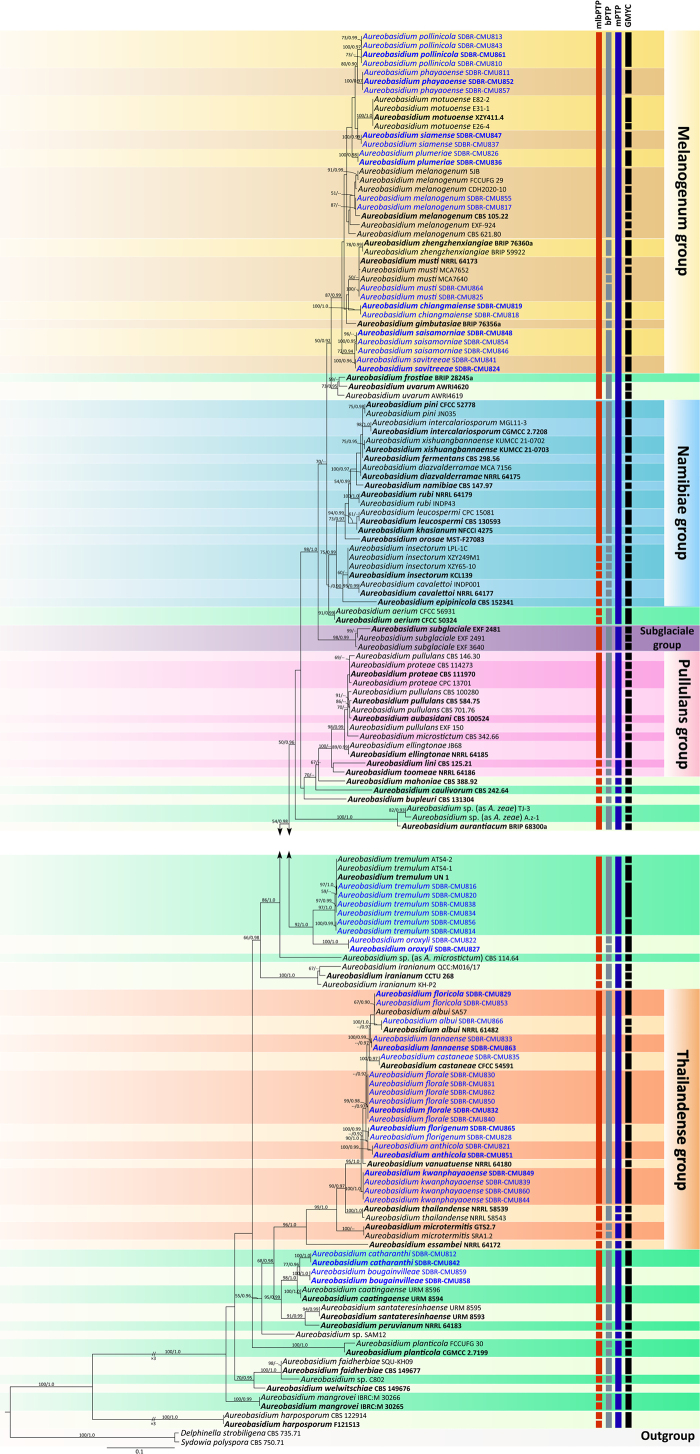
Phylogenetic tree generated from maximum-likelihood analysis of *Aureobasidium* based on a combined multilocus dataset of ITS, LSU, *rpb2*, *tef1*, and *tub* sequences. Maximum-likelihood bootstrap support values (MLBS) ≥ 50% and Bayesian posterior probabilities (BIPP) ≥ 0.90 are shown above the nodes as MLBS/BIPP. The tree is rooted with *Delphinella strobiligena* CBS 735.71 and *Sydowia polyspora* CBS 750.71. The fungal strains obtained in this study are shown in blue. Ex-type strains are shown in bold. Colored vertical bars on the right indicate the results of species delimitation (mlbPTP, red; bPTP, gray; mPTP, blue; and GMYC, black). The scale bar represents the expected number of nucleotide substitutions per site.

Phylogenetic analyses were based on a concatenated multilocus dataset of ITS, LSU, *rpb2*, *tef1*, and *tub* sequences comprising 155 taxa from representative strains of *Aureobasidium* species, including the outgroups. The total alignment comprised 3164 characters, including gaps (ITS: 1–612; LSU: 613–1474; *rpb2*: 1475–2398; *tef1*: 2399–2734; *tub*: 2735–3164). ML and BI analyses resulted in similar tree topologies that were consistent with those of previous studies (da Silva et al. 2024; Czachura and Piątek 2025; Jumbam et al. 2025). The best-scoring tree from the RAxML analysis, with a final ML optimization likelihood value of –23264.237773, is presented in Fig. 2. The RAxML analysis of the combined multilocus dataset had 1,455 distinct alignment patterns, with 34.98% undetermined characters or gaps. The estimated base frequencies were as follows: A = 0.247157, C = 0.245183, G = 0.268181, T = 0.239479; substitution rates AC = 1.991704, AG = 4.332866, AT = 1.683882, CG = 1.084287, CT = 10.819685, GT = 1.000000; gamma distribution shape parameter α = 0.539966. The average standard deviation of split frequencies reached 0.009496.

Multilocus phylogenetic analyses revealed that the newly generated sequences from 54 strains grouped within *Aureobasidium*, representing 16 novel species and five previously known species (*A. albui* SDBR-CMU866, *A. castaneae* SDBR-CMU835, *A. melanogenum* SDBR-CMU817 and SDBR-CMU855, *A. musti* SDBR-CMU825 and SDBR-CMU864, and *A. tremulum* SDBR-CMU814, SDBR-CMU816, SDBR-CMU820, SDBR-CMU834, SDBR-CMU838, and SDBR-CMU856) (Fig. 2). Furthermore, most *Aureobasidium* species could be grouped in accordance with previous studies into the *A. namibiae*, *A. melanogenum*, *A. pullulans*, *A. subglaciale*, and *A. thailandense* species groups (Zalar et al. 2008; Jumbam et al. 2025). The *A. melanogenum* group comprised *A. gimbutasiae*, *A. melanogenum*, *A. motuoense*, *A. musti*, *A. zhengzhenxiangiae*, and together with 18 strains representing seven new species (*A. chiangmaiense* SDBR-CMU818 and SDBR-CMU818, *A. phayaoense* SDBR-CMU811, SDBR-CMU852, and SDBR-CMU857, *A. plumeriae* SDBR-CMU826 and SDBR-CMU836, *A. pollinicola* SDBR-CMU810, SDBR-CMU813, SDBR-CMU843, and SDBR-CMU861, *A. saisamorniae* SDBR-CMU846, SDBR-CMU848, and SDBR-CMU854, *A. savitreeae* SDBR-CMU824 and SDBR-CMU841, and *A. siamense* SDBR-CMU836 and SDBR-CMU847) identified in this study, with 87% MLBS and 0.99 BIPP support. Additionally, the *A. thailandense* group comprised six previously described species (*A. albui*, *A. castaneae*, *A. essambei*, *A. microtermitis*, *A. thailandense*, and *A. vanuatuense*) and 18 strains representing six new species (*A. anthicola* SDBR-CMU821 and SDBR-CMU851, *A. florale* SDBR-CMU830, SDBR-CMU831, SDBR-CMU832, SDBR-CMU840, SDBR-CMU850, and SDBR-CMU862, *A. floricola* SDBR-CMU829 and SDBR-CMU853, *A. florigenum* SDBR-CMU828 and SDBR-CMU865, *A. kwanphayaoense* SDBR-CMU839, SDBR-CMU844, SDBR-CMU849, and SDBR-CMU860, and *A. lannaense* SDBR-CMU833 and SDBR-CMU863) identified in this study. Three additional new *Aureobasidium* species, namely *A. bougainvilleae* (SDBR-CMU858 and SDBR-CMU859), *A. catharanthi* (SDBR-CMU812 and SDBR-CMU842), and *A. oroxyli* (SDBR-CMU822 and SDBR-CMU827), and one known species, *A. tremulum* (SDBR-CMU814, SDBR-CMU816, SDBR-CMU820, SDBR-CMU834, SDBR-CMU838, and SDBR-CMU856), were resolved as distinct monophyletic lineages separate from other previously known species. Furthermore, GCPSR, using the pairwise homoplasy index (PHI), was applied to confirm the delimitation of new species within both the *A. melanogenum* and *A. thailandense* groups.

### Species delimitation analyses

#### Coalescent-based species recognition (CBSR)

Among the PTP-based methods (Fig. 2), the highest number of putative species was delimited by bPTP (49 species), followed by mlbPTP (39 species) and mPTP (23 species). The mPTP method grouped members of the *A. melanogenum* and *A. namibiae* groups, together with *A. aerium*, *A. frostiae*, and *A. uvarum*, into a single species, whereas bPTP delimited the strains of *A. aerium* as a single distinct species, and mlbPTP separated each strain as an individual species. Within the *A. melanogenum* group, mlbPTP delimited all strains, including *A. frostiae* and *A. uvarum*, as a single species, whereas bPTP delimited eight species. Notably, *A. frostiae* and two strains of *A. uvarum* were treated as conspecific by bPTP. Within the *A. namibiae* group, mlbPTP delimited six species: species 1 comprised *A. diazvalderramae*, *A. fermentans*, *A. intercalariosporum*, *A. khasianum*, *A. leucospermi*, *A. namibiae*, *A. orasae*, *A. pini*, *A. rubi*, and *A. xishuangbannaense*; species 2 comprised *A. insectorum* strains LPL-1C and XZY249M1; species 3 comprised *A. insectorum* strain XZY65-10; species 4 comprised *A. insectorum* strain KCL139 (type strain); species 5 corresponded to *A. cavalettoi*; and species 6 corresponded to *A. epipinicola*. In contrast, the bPTP analysis recognized nine species: species 1 comprised *A. diazvalderramae*, *A. fermentans*, *A. intercalariosporum*, *A. khasianum*, *A. leucospermi*, *A. namibiae*, *A. pini*, *A. rubi*, and *A. xishuangbannaense*, whereas species 2–9 were delimited as distinct species represented by individual strains of *A. cavalettoi*, *A. epipinicola*, *A. insectorum*, and *A. orasae*. Within the *A. subglaciale* group, mlbPTP, bPTP, and mPTP grouped the three strains into a single species. In the *A. pullulans* group, most taxa were delimited as distinct species by all PTP-based methods, except for *A. lini* and *A. toomeae*, which were grouped as one species by mPTP but resolved as distinct species by mlbPTP and bPTP. *Aureobasidium aurantiacum* and *Aureobasidium* sp. (as *A. zeae*) were treated as the same species by all PTP-based methods. For *A. iranianum*, mlbPTP and bPTP produced consistent results, whereas mPTP grouped all strains into a single species. Within the *A. thailandense* group, all PTP-based methods grouped *A. albui*, *A. anthicola*, *A. castaneae*, *A. florale*, *A. floricola*, *A. florigenum*, *A. kwanphayaoense*, *A. lannaense*, and *A. vanuatuense* as a single species. Two strains of *A. thailandense* were identified as a single species by mlbPTP and bPTP, whereas mPTP separated them as distinct species. The mlbPTP and bPTP methods each separated the two strains of *A. microtermitis* as individual species, whereas mPTP grouped them into a single species. *Aureobasidium essambei* was consistently treated as a distinct species by all PTP-based methods. The mPTP method grouped *A. bougainvilleae*, *A. caatingaense*, *A. catharanthi*, *A. peruvianum*, and *A. santateresinhaense* into a single species. Similarly, *A. faidherbiae*, *Aureobasidium* sp. C802, and *A. welwitschiae* were also grouped into a single species, whereas *A. harposporum*, *A. mangrovei*, *A. planticola*, and *Aureobasidium* sp. SAM12 were delimited as distinct species. Both mlbPTP and bPTP grouped *A. bougainvilleae*, *A. caatingaense*, and *A. catharanthi* into a single species, whereas *A. faidherbiae*, *A. peruvianum*, *A. santateresinhaense*, *Aureobasidium* sp. C802, *Aureobasidium* sp. SAM12, and *A. welwitschiae* were each delimited as a distinct species. Finally, *A. planticola* in bPTP and *A. harposporum* and *A. mangrovei* in mlbPTP were split such that each strain was delimited as an individual species.

The GMYC analysis identified 96 species hypotheses within *Aureobasidium* (Fig. 2). The analysis recovered species boundaries largely consistent with those inferred from the multilocus phylogenetic analyses. However, *A. insectorum*, *A. melanogenum*, *A. motuoense*, *A. musti*, *A. pullulans*, and *A. tremulum* were not well delimited. In addition, strains of *A. albui*, *A. iranianum*, *A. planticola*, *A. proteae*, *A. subglaciale*, *A. thailandense*, and *A. uvarum* were split into distinct species by GMYC. Notably, *A. albui* SA57 was treated as conspecific with *A. floricola*, consistent with the combined phylogenetic analyses.

The results of the species delimitation analyses revealed that the PTP-based methods were generally inconsistent with the multilocus phylogenetic analyses. In several lineages, PTP approaches either over-lumped distinct taxa into a single species (e.g., in the *A. melanogenum* and *A. namibiae* groups) or over-split closely related strains. In contrast, the GMYC model revealed a higher level of congruence with the multilocus phylogenetic analyses. Most species boundaries were consistent with the phylogenetic tree, indicating that GMYC more accurately captured evolutionary divergence among lineages. GMYC tended to separate strains within *A. iranianum* and *A. thailandense*; however, it was more effective in resolving species-level distinctions overall.

#### Genealogical concordance phylogenetic species recognition (GCPSR)

**Figure 3.**
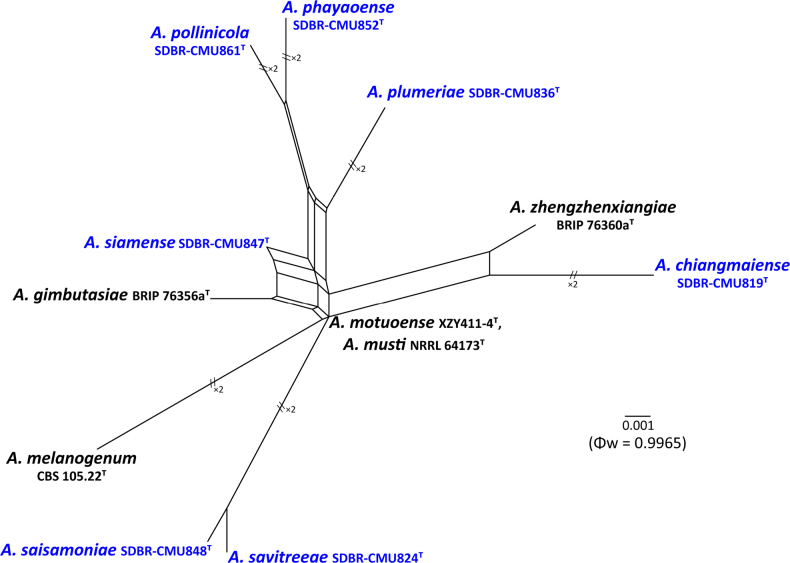
Splits graph from the pairwise homoplasy index (PHI) test generated from a concatenated multilocus dataset of ITS, LSU, *rpb2*, *tef1*, and *tub* sequences of closely related taxa in the *Aureobasidium melanogenum* group using the LogDet transformation and split decomposition. PHI test results (Φw) < 0.05 indicate significant recombination within the dataset. The strains obtained in this study are shown in bold blue type. Ex-type strains are marked with a superscript “T.”

**Figure 4.**
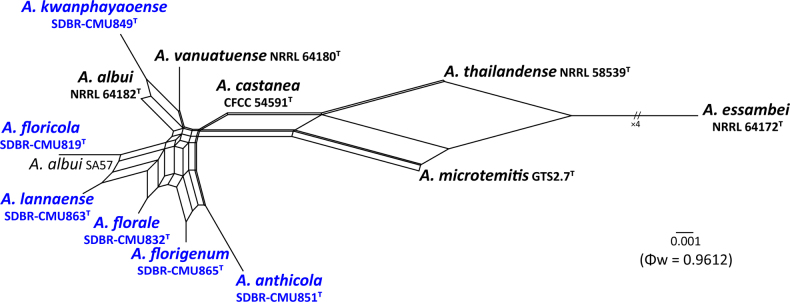
Splits graph from the pairwise homoplasy index (PHI) test generated from a concatenated multilocus dataset of ITS, LSU, *rpb2*, *tef1*, and *tub* sequences of closely related taxa in the *Aureobasidium thailandense* group using the LogDet transformation and split decomposition. PHI test results (Φw) < 0.05 indicate significant recombination within the dataset. The strains obtained in this study are shown in bold blue type. Ex-type strains are marked with a superscript “T.”

The level of recombination among newly generated strains and representative strains of phylogenetically closely related species in the *A. melanogenum* and *A. thailandense* groups was assessed using the PHI test based on a combined multilocus dataset of ITS, LSU, *rpb2*, *tef1*, and *tub* sequences. The PHI result showed no evidence of significant recombination (Φw = 0.9965) among *A. chiangmaiense*, *A. gimbutasiae*, *A. melanogenum*, *A. motuoense*, *A. musti*, *A. phayaoense*, *A. plumeriae*, *A. pollinicola*, *A. saisamorniae*, *A. savitreeae*, *A. siamense*, and *A. zhengzhenxiangiae* in the *A. melanogenum* group (Fig. 3). In the *A. thailandense* group, no significant recombination was detected (Φw = 0.9612) among *A. anthicola*, *A. albui*, *A. castaneae*, *A. essambei*, *A. floricola*, *A. florigenum*, *A. florale*, *A. kwanphayaoense*, *A. lannaense*, *A. microtermitis*, *A. thailandense*, and *A. vanuatuense* (Fig. 4).

#### Physiological characterization

Physiological tests were performed for the 16 newly identified *Aureobasidium* species described in this study, and the results are presented in Table 1. The results revealed distinct carbon assimilation patterns that provided valuable phenotypic evidence supporting the delimitation of the newly described species from their closest phylogenetic relatives. Glucose fermentation was negative for all 16 *Aureobasidium* species. Ammonium sulfate, ethylamine HCl, *L*-lysine, cadaverine, and creatine were assimilated as nitrogen sources by all *Aureobasidium* species, whereas none assimilated potassium nitrate or sodium nitrite. During the carbon assimilation tests, *A. anthicola* produced light brown pigmentation when grown in salicin, *D*-glucono-1,5-lactone, and *D*-gluconate. *Aureobasidium siamense* produced light brown pigmentation along with dark brown sediment when grown in *D*-glucono-1,5-lactone. In contrast, *A. oroxyli* developed light pink pigmentation when grown in *D*-glucose and was also the only species unable to assimilate *N*-acetyl-*D*-glucosamine and galactitol. *Aureobasidium savitreeae* produced light pink pigmentation and dark brown sediment during the assimilation of *D*-glucose, *D*-xylose, *L*-arabinose, sucrose, raffinose, melezitose, and erythritol. Moreover, none of the *Aureobasidium* species assimilated soluble starch or methanol. Only *A. chiangmaiense* was able to assimilate inulin. Among the strains examined, *A. floricola* and *A. phayaoense* did not assimilate methyl-*α*-*D*-glucoside and succinate, respectively. The inability to assimilate *DL*-lactate was observed in *A. chiangmaiense*, *A. florale*, *A. oroxyli*, and *A. phayaoense*. Detailed comparisons of the physiological characteristics of the newly described species with those of their closely related species are presented below.

**Table 1.** Assimilation profiles of carbon and nitrogen sources and fermentation ability of the *Aureobasidium* strains studied.

**Compounds**	**1**	**2**	**3**	**4**	**5**	**6**	**7**	**8**	**9**	**10**	**11**	**12**	**13**	**14**	**15**	**16**
**Fermentation**																
Glucose	-	-	-	-	-	-	-	-	-	-	-	-	-	-	-	-
**Carbon assimilation**																
*D*-glucose	++	+ ^L^	+ ^L^	+ ^L^	+ ^L^	+ ^D^	+ ^D^	+ ^B^	+ ^B^	+^**^	+ ^B^	+ ^B^	+ ^B^	+ ^B^	+^**L^	+ ^V^
*D*-galactose	+ ^D^	+ ^B^	+ ^L^	+ ^L^	+ ^L^	+ ^D^	+ ^V^	+	++	++	+ ^B^	w ^B^	+ ^B^	w ^B^	w ^L^	+ ^D^
*L*-sorbose	++	+ ^B^	+ ^L^	+	w ^L^	+ ^D^	+ ^B^	+ ^D^	++	++	+ ^B^	w ^B^	+ ^B^	w ^B^	w ^L^	+ ^D^
*N*-acetyl glucosamine	++	+ ^D^	w ^D^	+ ^L^	+ ^L^	+ ^D^	++	++	++	-	w ^D^	+ ^B^	+ ^B^	+ ^B^	w ^L^	+ ^V^
*D*-ribose	++	++	++	w	w ^B^	++	+ ^V^	++	++	+ ^B^	+ ^B^	+ ^V^	+ ^B^	+ ^B^	w ^L^	+ ^V^
*D*-xylose	++	+ ^D^	++	+ ^D^	+ ^B^	++	+ ^V^	++	++	+ ^L^	+ ^D^	+ ^B^	+ ^L^	+ ^B^	+^**L^	+ ^B^
*L*-arabinose	++	++	++	++	w ^B^	+ ^V^	+ ^V^	+ ^B^	++	+ ^L^	+ ^D^	+ ^B^	w ^B^	+ ^B^	+^**L^	+ ^V^
*D*-arabinose	+ ^D^	+ ^B^	++	++	w ^L^	w	++	++	w ^B^	++	+ ^D^	+ ^V^	+ ^B^	w ^B^	w ^L^	+ ^V^
*L*-rhamnose	++	+ ^D^	++	++	+ ^L^	+ ^D^	+ ^V^	++	++	++	+ ^D^	+ ^B^	w ^B^	+ ^B^	+ ^L^	+ ^B^
Sucrose	+ ^D^	w	++	++	+ ^L^	+	++	++	++	++	+ ^D^	+ ^B^	w ^B^	+ ^B^	+^**L^	+ ^V^
Maltose	++	+ ^D^	++	++	+ ^L^	+ ^D^	++	++	++	++	+ ^D^	+ ^B^	+ ^B^	+ ^B^	w ^L^	+ ^V^
*α-α-*trehalose	++	+ ^D^	++	+ ^L^	w ^L^	+ ^D^	++	++	++	++	+ ^D^	+ ^B^	w ^B^	w ^B^	+ ^L^	+ ^V^
Methyl-*α*-*D*-glucoside	+ ^D^	+ ^D^	w ^B^	++	w ^B^	-	+ ^D^	+ ^B^	w ^B^	w	+ ^D^	+ ^B^	w ^B^	w ^B^	+ ^L^	w ^V^
Cellobiose	++	+ ^D^	+	+ ^L^	+ ^L^	+ ^B^	++	+	++	+ ^L^	+ ^D^	+ ^B^	w ^B^	+ ^B^	+ ^L^	w ^V^
Salicin	+^*^	+ ^D^	w ^L^	w ^D^	w ^L^	+ ^D^	++	+ ^D^	++	+	+ ^D^	+ ^B^	w ^B^	+ ^B^	w ^L^	+ ^D^
Melibiose	++	+ ^D^	+	w ^L^	+ ^L^	++	++	++	++	w ^L^	w ^D^	w ^B^	w ^B^	w ^B^	w ^L^	w ^V^
Lactose	++	+ ^D^	w	++	w ^L^	++	++	++	+ ^V^	++	+ ^D^	w ^B^	+ ^B^	+ ^B^	w ^L^	w ^V^
Raffinose	+ ^D^	+ ^L^	++	w ^D^	+ ^L^	++	++	+ ^B^	++	+ ^L^	+ ^D^	+ ^B^	w ^B^	+ ^B^	+^**L^	w ^V^
Melizitose	++	++	++	+ ^L^	+ ^L^	++	++	++	++	++	+ ^D^	w ^B^	+ ^B^	+ ^B^	+^**L^	w ^V^
Inulin	-	-	-	w	-	-	-	-	-	-	-	-	-	-	-	-
Soluble starch	-	-	-	-	-	-	-	-	-	-	-	-	-	-	-	-
Glycerol	++	+ ^B^	w ^B^	+ ^L^	w ^B^	+ ^D^	++	+ ^B^	++	++	+ ^D^	w ^B^	+ ^B^	+ ^B^	w ^L^	+ ^V^
Erythritol	++	+ ^B^	w ^B^	w ^B^	w ^L^	+ ^B^	+ ^D^	+ ^D^	++	++	+ ^D^	w ^B^	w ^L^	+ ^B^	w^**L^	w ^V^
Ribitol	++	++	w ^D^	++	w ^L^	++	++	+	++	w	+ ^D^	+ ^B^	+ ^B^	+ ^B^	w ^L^	+ ^V^
*D*-glucitol	++	++	++	++	w ^L^	++	++	+ ^B^	++	w	+ ^D^	+ ^B^	+ ^B^	+ ^B^	+ ^L^	+ ^V^
*D*-mannitol	++	+ ^B^	++	++	w ^L^	++	++	++	++	+ ^D^	+ ^B^	+ ^B^	w ^L^	+ ^B^	w ^L^	w ^V^
Galactitol	++	++	++	++	w ^B^	+	+ ^V^	++	++	-	+ ^D^	+ ^B^	w ^L^	w ^B^	+ ^L^	+ ^V^
*myo*-Inositol	++	+ ^D^	++	w	w ^L^	+ ^D^	+ ^D^	+ ^B^	++	++	+ ^B^	w ^B^	w ^L^	+ ^B^	w ^L^	w ^V^
*D*-glucono-1,5-lactone	+^*^	+ ^D^	w ^B^	+ ^D^	w ^B^	+ ^B^	++	+ ^B^	++	++	+ ^D^	+ ^B^	w ^B^	+ ^B^	w ^L^	+*^D^
*D*-gluconate	+ ^D^	+ ^B^	+ ^B^	++	w ^B^	+ ^B^	w ^B^	+ ^B^	+ ^B^	+ ^D^	+ ^D^	+ ^B^	+ ^B^	w ^B^	w ^L^	w ^B^
*D*-glucuronate	+^*^	++	+ ^B^	++	w ^L^	+ ^D^	+ ^D^	++	++	++	+ ^D^	w ^B^	w ^B^	+ ^B^	w ^L^	w ^V^
*D*-galacturonic acid	++	w ^L^	w ^L^	++	w ^L^	+ ^D^	++	++	++	w	w ^D^	w ^B^	+ ^B^	w ^B^	w ^L^	w ^V^
*DL*-lactate	++	w	w	-	-	w ^D^	++	++	w	-	-	+ ^B^	w ^B^	+ ^B^	+ ^L^	w ^V^
Succinate	++	++	w ^L^	++	w ^L^	+ ^B^	++	++	++	++	-	w ^B^	+ ^B^	+ ^B^	+ ^L^	w ^V^
Citrate	++	w ^D^	w ^L^	++	+ ^L^	+ ^D^	++	++	++	++	+ ^D^	w ^B^	+ ^B^	+ ^B^	+ ^L^	w ^V^
Methanol	-	-	-	-	-	-	-	-	-	-	-	-	-	-	-	-
Ethanol	++	++	++	w	w ^L^	++	+ ^B^	+ ^B^	++	++	+ ^V^	+ ^V^	w ^B^	+ ^B^	w ^L^	w ^V^
Xylitol	++	++	++	w	w ^B^	++	++	++	w	++	+ ^B^	+ ^V^	w ^B^	+ ^B^	w ^L^	w ^V^
**Nitrogen assimilation**																
Ammonium sulfate	+	+	+	+	+	+	+	+	+	+	+	+	+	+	+	+
Potassium nitrate	-	-	-	-	-	-	-	-	-	-	-	-	-	-	-	-
Sodium nitrite	-	-	-	-	-	-	-	-	-	-	-	-	-	-	-	-
Ethylamine HCl	+	+	+	+	+	+	+	+	+	+	+	+	+	+	+	+
*L-*lysine	+	+	+	+	+	+	+	+	+	+	+	+	+	+	+	+
Cadaverine	+	+	+	+	+	+	+	+	+	+	+	+	+	+	+	+
Creatine	+	+	+	+	+	+	+	+	+	+	+	+	+	+	+	+

Strain 1: *A. anthicola* SDBR-CMU851, 2: *A. bougainvilleae* SDBR-CMU858, 3: *A. catharanthi* SDBR-CMU842, 4: *A. chiangmaiense* SDBR-CMU819, 5: *A. florale* SDBR-CMU832, 6: *A. floricola* SDBR-CMU829, 7: *A. florigenum* SDBR-CMU865, 8: *A. kwanphayaoense* SDBR-CMU849, 9: *A. lannaense* SDBR-CMU863, 10: *A. oroxyli* SDBR-CMU827, 11: *A. phayaoense* SDBR-CMU852, 12: *A. plumeriae* SDBR-CMU836, 13: *A. pollinicola* SDBR-CMU861, 14: *A. saisamorniae* SDBR-CMU848, 15: *A. savitreeae* SDBR-CMU824, and 16: *A. siamense* SDBR-CMU847. Characteristics: strong assimilation/fermentation (++), assimilation/fermentation (+), weak assimilation/ fermentation (w), negative assimilation/fermentation (-); pigmentation: light brown pigmentation (*), light pink pigmentation (**); sediment: black sediment (superscript B), white sediment (superscript V), light brown sediment (superscript L), dark brown sediment (superscript D).

### Taxonomy

#### Aureobasidium albui

Taxon classificationFungiDothidealesSaccotheciaceae

Jumbam & Aime, Persoonia 54: 171 (2025)

F071C410-EBAE-59E0-829D-0B9E0EA6483E

848255

Fig. 5

##### Cultural characteristics.

After 14 days of incubation at 25 °C in the dark, colonies on PDA reaching 32–40 mm diam., surface flat, spreading, round with radiating, irregular margin, velvety, slimy, fluffy, yellowish white (2A2), paler at the margin; on MEA reaching 40–50 mm diam., spreading, round with radiating, irregular margin, velvety, slimy, grayish brown (7F3) at center, becoming yellowish brown (5E4) to smoke brown (4E2) and white at the margin; on OA 35–47 mm diam., surface flat, spreading with entire margin, velvety, fluffy, whitish gray, light gray at the margin.

**Figure 5.**
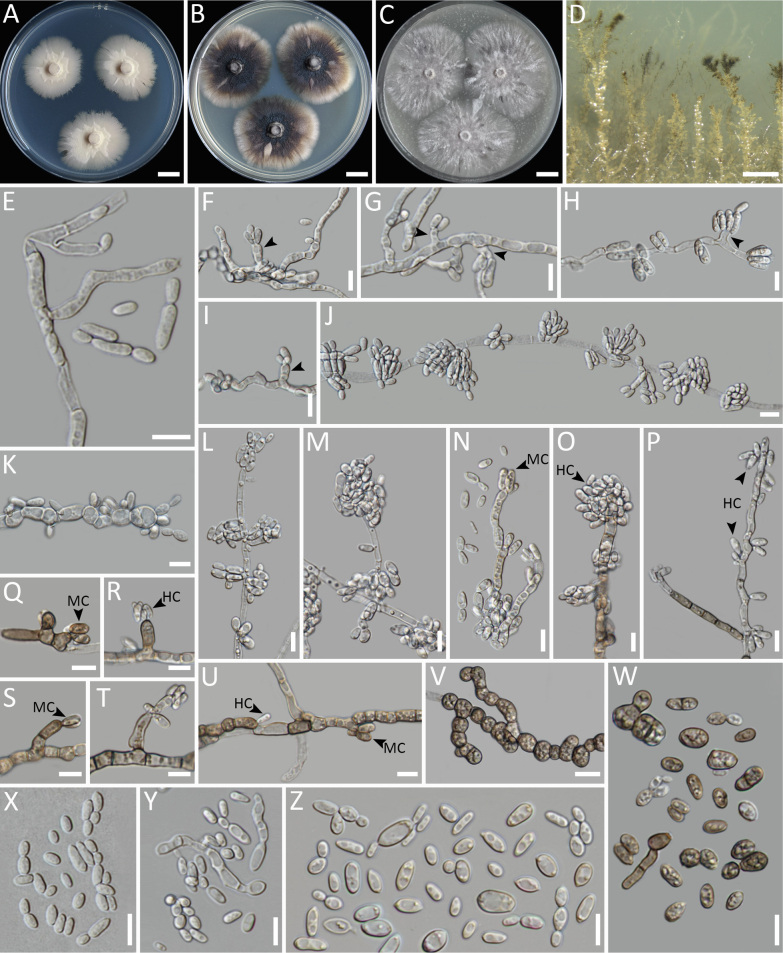
*Aureobasidium albui* (SDBR-CMU866). **A–C** colonies on PDA, MEA, and OA, respectively, after 14 d at 25 °C; **D** close-up of an immersed hyphal-tip colony on PDA after 14 d; **E** endoconidia; **F–I** conidiogenous cells (arrows) integrated into vegetative hyphae with hyaline conidia; **J, K** conidiogenous apparatus; **L–P** hyaline and melanized hyphae with groups of hyaline conidia (HC) and melanized conidia (MC); **Q–U***Hormonema*-like conidiogenous cells with hyaline conidia; **V** melanized hyphae developing into chlamydospores; **W** melanized conidia produced from melanized hyphae and chlamydospores; **X–Z** conidia, some exhibiting yeast-like budding, and germinating conidia. Scale bars: 1 cm (**A–C**); 500 μm (**D**); 10 μm (**E–W**).

##### Description.

***Anamorph****in vitro* dematiaceous hyphomycetes. ***Vegetative hyphae*** (2.6–)3.5–7.5(–9.6) μm wide, mostly hyaline, branched, septate, smooth, thin-walled; become light brown to brown with age, constricted at the septa, thick-walled. ***Conidiogenous cells*** (4.4–)6.0–11.0(–16.2) × 2.5–5.4(–6.7) μm (*x̄* = 8.6 × 4.3 μm, *n* = 25), mostly undifferentiated, intercalary, rarely terminal, producing conidia percurrently from indistinct, short lateral denticles, or differentiated on hyaline hyphae, occasionally elongated, solitary, becoming light brown to dark brown with age. ***Conidia*** (4.3–)5.9–9.8(–13.6) × (2.3–)3.2–5.6(–7.0) μm (*x̄* = 8.0 × 4.4 μm, *n* = 180), fusoid, obovoid to ellipsoidal or occasionally elongate, hyaline to light brown, aseptate, guttulate, smooth-walled, becoming brown with age, budding occasionally observed. ***Chlamydospores*** (5–)6.5–9.5(–11.2) μm wide (*x̄* = 8.1 μm, *n* = 35), produced in aerial hyphae, mostly intercalary, solitary or in branched chains, subglobose to ellipsoidal, pigmented, light to dark brown, smooth-, thick-walled, septate, 1–2 cells, constricted near the septa. ***Endoconidia*** (3.7–)4.2–5.8 × 2.3–3.5 μm (*x̄* = 4.9 × 2.9 μm, *n* = 20), obovoid to ellipsoidal, hyaline, occasionally seen in intercalary hyphal cells.

##### Cardinal temperatures for growth.

Minimum 4 °C, optimum in the range of 25 °C to 30 °C. No growth at 37 °C.

##### Strain examined.

THAILAND • Chiang Mai Province, Mueang District, Suthep, isolated from white cheesewood flower (*Alstonia scholaris*; *Apocynaceae*, *Gentianales*), Sep 2024, P. Kodchasee, C. Senwanna, J. Kumla and N. Suwannarach, living culture = SDBR-CMU866.

##### Habitat and distribution.

Phylloplane of *Cyrtomium falcatum* and unidentified leaf in the USA (Jumbam et al. 2025); flowers of *Alstonia scholaris* in Thailand (this study).

##### Notes.

In the phylogenetic tree, strain SDBR-CMU866 formed a well-supported clade with *A. albui* NRRL 61482 (ex-type), with 100% MLBS and 1.0 BIPP support (Fig. 2). Morphologically, strain SDBR-CMU866 is generally consistent with the type description of *A. albui* by Jumbam et al. (2025); however, detailed observations of some structures (e.g., conidiophores and conidiogenous cells) were not provided. Therefore, strain SDBR-CMU866 was identified as *A. albui*, and an updated morphological description of the species is provided herein. Prior to this study, *A. albui* had been reported only from the leaf phylloplane in the USA (Jumbam et al. 2025). This study represents the first report of *A. albui* in Thailand.

#### Aureobasidium anthicola

Taxon classificationFungiDothidealesSaccotheciaceae

Senwanna, Kodchasee, J. Kumla & N. Suwannar. sp. nov.

6CC00DCE-EC20-5DB2-BA5E-04B4820B8C9F

861957

Fig. 6

##### Etymology.

Refers to “inhabiting flowers,” the substrate from which this species was first isolated.

**Figure 6.**
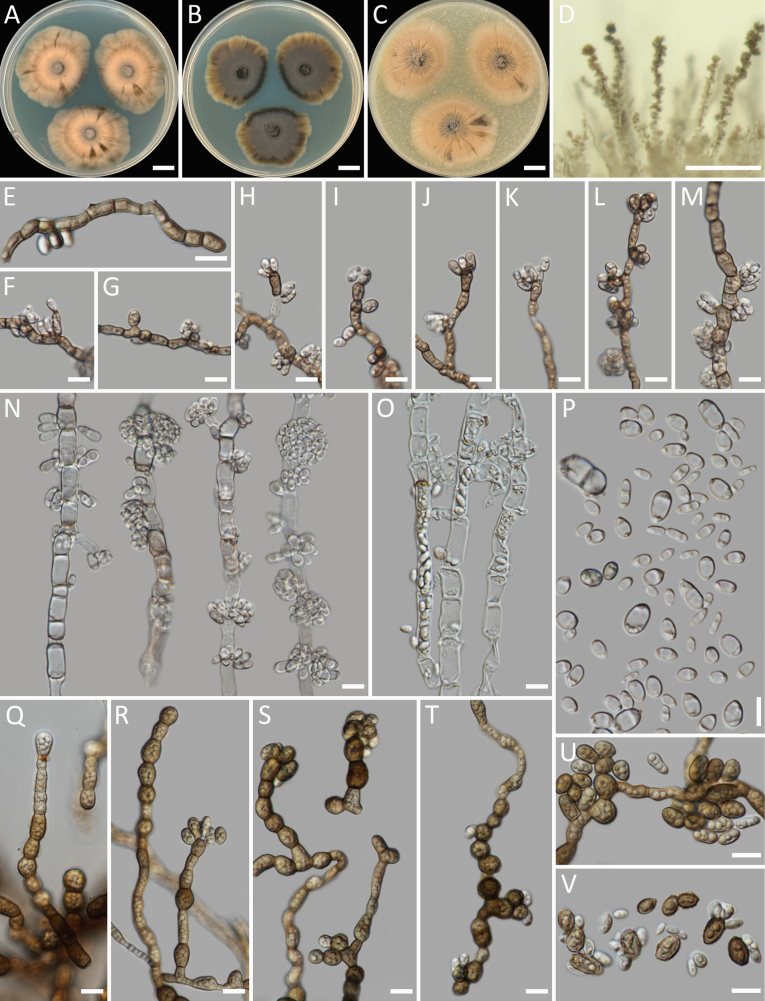
*Aureobasidium anthicola* (SDBR-CMU851, ex-type). **A–C** colonies on PDA, MEA, and OA, respectively, after 14 d at 25 °C; **D** close-up of an immersed hyphal-tip colony on PDA after 14 d; **E** hyphae with multiple lateral pegs; **F–K** conidiogenous cells integrated into vegetative hyphae with conidia; **L, M** conidiogenous apparatus; **N** hyphae with a group of conidia and a conidiogenous apparatus; **O** endoconidia; **P** conidia; **Q–S** melanized hyphae with groups of melanized conidia; **T** chlamydospores budding to produce hyaline conidia; **U, V** melanized conidia. Scale bars: 1 cm (**A–C**); 500 μm (**D**); 10 μm (**E–V**).

##### Type.

THAILAND • Phayao Province, Mueang District, isolated from bridal bouquet flower (*Plumeria pudica*; *Apocynaceae*, *Gentianales*), Aug 2024, P. Kodchasee, C. Senwanna, J. Kumla and N. Suwannarach, ***holotype*** = CMUB40138 (preserved in metabolically inactive state), living culture (ex-type) = SDBR-CMU851 = GMBCC2507.

##### Cultural characteristics.

After 14 days of incubation at 25 °C in the dark, colonies on PDA reaching 35–43 mm diam., surface flat, spreading, round with radiating margin, velvety, concentric, light gray (3D1) at center, pale orange (6A3), olive brown (4E5); on MEA reaching 30–37 mm diam., surface flat, spreading, round with radiating margin, velvety, brownish gray (4E2) to black, with olive brown at the margin; on OA 36–40 mm diam., surface flat, spreading with filiform, with entire margin, wrinkled, light gray (3D1) at center, pale orange (6A3) mixed with olive brown (4E5).

##### Description.

***Anamorph****in vitro* dematiaceous hyphomycetes. ***Vegetative hyphae*** (1.9–)2.9–7.7(–9) μm wide, hyaline to dark brown, branched, septate, smooth, thin-walled, becoming light brown to brown or golden brown with age, constricted at the septa. ***Conidiogenous cells*** (7.0–)9.1–14.0(–16.0) × 4.0–6.0(–8.9) μm (*x̄* = 11.4 × 6.2 μm, *n* = 25), undifferentiated, intercalary, terminal, producing conidia percurrently from indistinct, short lateral denticles, giving rise to conidia that aggregate in a mucoid mass, or differentiated on hyphae, hyaline to dark brown in age, occasionally elongated, solitary. ***Conidia*** (4.2–)6.3–10.5(–13.0) × (2.5–)3.4–6.0(–8.0) μm (*x̄* = 8.3 × 4.2 μm, *n* = 200), obovoid to ellipsoidal, hyaline to light brown, aseptate, guttulate, smooth-walled, becoming light brown to brown with ages. ***Chlamydospores*** (5.7–)7.2–10.4(–12.3) μm wide (*x̄* = 8.7 μm, *n* = 45), produced in aerial hyphae, mostly intercalary, solitary or in branched chains, subglobose to ellipsoidal, pigmented, light to dark brown, smooth-, thick-walled, septate, constricted near the septa. ***Endoconidia*** (2.7–)3.6–6.0(–6.6) × 2.2–3.1(–3.7) μm (*x̄* = 4.8 × 2.8 μm, *n* = 40), ellipsoidal, hyaline, occasionally seen in intercalary hyphal cells.

Fermentation of glucose is negative. *D*-glucose, galactose, sorbose, *N*-acetyl glucosamine, ribose, xylose, *L*-arabinose, *D*-arabinose, *L*-rhamnose, sucrose, maltose, *α*-*α*-trehalose, methyl-*α*-*D*-glucoside, cellobiose, salicin, melibiose, lactose, raffinose, melizitose, glycerol, erythritol, ribitol, glucitol, mannitol, galactitol, *myo*-inositol, *D*-glucono-1,5-lactone, *D*-gluconate, *D*-glucuronate, *D*-galacturonic acid, *DL*-lactate, succinate, citrate, ethanol, and xylitol are assimilated, but inulin, soluble starch, and methanol are not assimilated. Ammonium sulfate, ethylamine hydrochloride, *L*-lysine, cadaverine, and creatine are assimilated as sole nitrogen sources. Potassium nitrate and sodium nitrite are not assimilated. After 3 weeks, light brown pigmentation was observed in salicin, *D*-glucono-1,5-lactone, and *D-*glucuronate, while dark brown sediment formed in *D*-galactose, *D*-arabinose, sucrose, methyl-*α*-*D*-glucoside, raffinose, and *D*-gluconate.

##### Cardinal temperatures for growth.

Minimum 10 °C, optimum in the range of 25 °C to 30 °C. No growth at 4 °C and 37 °C.

##### Additional strains examined.

THAILAND • Chiang Mai Province, Mueang District, Suthep, isolated from Fukien tea flower (*Ehretia microphylla*; *Boraginaceae*, *Boraginales*), Jul 2024, P. Kodchasee, C. Senwanna, J. Kumla and N. Suwannarach, living culture = SDBR-CMU821.

##### Habitat and distribution.

Flowers of *Ehretia microphylla* and *Plumeria pudica*. Known only from northern Thailand.

##### Notes.

Based on the multilocus phylogenetic analyses, *A. anthicola* SDBR-CMU821 and SDBR-CMU851 (ex-type) formed a well-supported monophyletic lineage (100% MLBS and 0.99 BIPP) that was sister to *A. florigenum* (Fig. 2). The *rpb2* and *tef1* sequences of *A. anthicola* differed from those of *A. florigenum* by 1.74% (17/977 bp, including gaps) and 1.88% (5/266 bp, including gaps), respectively. Additionally, the PHI test confirmed the genetic distinctiveness of *A. anthicola* and *A. florigenum* (Fig. 4). Morphologically, colonies of *A. anthicola* on OA differed from those of *A. florigenum* by having a pale orange mixed with olive-brown color, whereas *A. florigenum* produced yellowish-white colonies mixed with light gray and with a brownish-gray margin. *Aureobasidium anthicola* produced endoconidia, whereas *A. florigenum* did not. Based on physiological characteristics, *A. anthicola* differed from *A. florigenum* by producing light brown pigment when grown in salicin, *D*-glucono-1,5-lactone, and *D*-glucuronate, which was not produced by *A. florigenum*. In addition, differences in sediment formation were observed during carbon assimilation (Table 1). Therefore, *A. anthicola* is introduced as a new species in the *A. thailandense* group.

#### Aureobasidium bougainvilleae

Taxon classificationFungiDothidealesSaccotheciaceae

Senwanna, Kodchasee, J. Kumla & N. Suwannar. sp. nov.

69BBB0AE-28E7-596D-A228-E1715A6371CD

861958

Fig. 7

##### Etymology.

Refers to the host genus *Bougainvillea*, from which this species was isolated.

**Figure 7.**
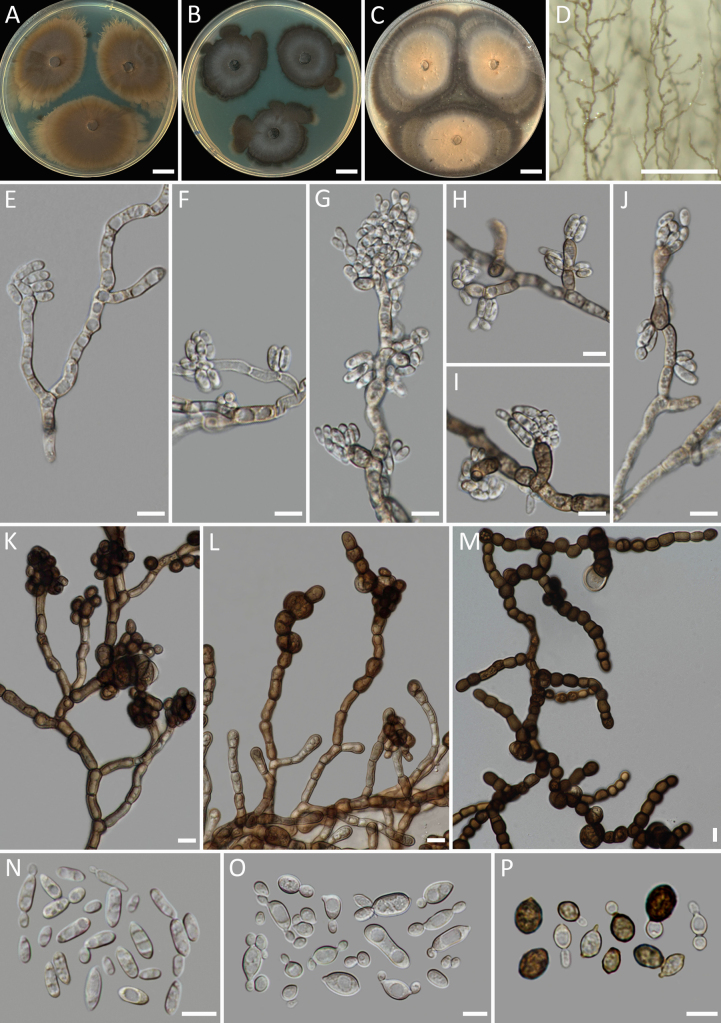
*Aureobasidium bougainvilleae* (SDBR-CMU858, ex-type). **A–C** colonies on PDA, MEA, and OA, respectively, after 14 d at 25 °C; **D** close-up of an immersed hyphal-tip colony on PDA after 14 d; **E–G** hyphae with groups of hyaline conidia; **H–J***hormonema*-like conidiogenous cells with hyaline conidia; **K** melanized hyphae with groups of melanized conidia; **L, M** melanized hyphae developing into chlamydospores; **N, O** conidia, some exhibiting yeast-like budding; **P** melanized conidia, some exhibiting yeast-like budding. Scale bars: 1 cm (**A–C**); 500 μm (**D**); 10 μm (**E–P**).

##### Type.

THAILAND • Phayao Province, Mueang District, isolated from bougainvillea (*Bougainvillea hybrid*; *Nyctaginaceae*, *Caryophyllales*), Aug 2024, P. Kodchasee, C. Senwanna, J. Kumla and N. Suwannarach, ***holotype*** = CMUB40141 (preserved in metabolically inactive state), living culture (ex-type) = SDBR-CMU858 = GMBCC2509.

##### Cultural characteristics.

After 14 days of incubation at 25 °C in the dark, colonies on PDA reaching 56–60 mm diam., surface flat, spreading, round with radiating, irregular margin, velvety, grayish brown (6E3), with grayish yellow (4B6) at the margin; on MEA reaching 35–44 mm diam., surface flat, spreading, round with radiating, lobate margin, velvety, brownish gray (4E2), grayish brown (6E3), and black at the margin; on OA 62–67 mm diam., surface flat, spreading with filiform, entire margin, velvety, slimy, pale orange (6A3), with yellowish brown (5E8), light brown (6D4) at the margin.

##### Description.

***Anamorph****in vitro* dematiaceous hyphomycetes. ***Vegetative hyphae*** 3.0–11.0(–19.0) μm, hyaline to brown, branched, septate, smooth, thin-walled, becoming light to dark brown with age, constricted at the septa, thick-walled. ***Conidiogenous cells*** 5.0–15.0(–17.0) × 3.5–8.8 μm (*x̄* = 12.2 × 5.1 μm, *n* = 25), undifferentiated, intercalary, terminal, producing conidia percurrently from indistinct, short lateral denticles, giving rise to conidia that aggregate in a mucoid mass, or differentiated on hyphae, occasionally elongated, solitary, becoming brown with age. ***Conidia*** (3.8–)6.0–11.5(–13.5) × (2.5–)3.5–7.0(–9.4) μm (*x̄* = 8.7 × 4.7 μm, *n* = 180), obovoid to ellipsoidal, elongated, or globose to subglobose, hyaline becoming light to dark brown with age, aseptate, occasionally with an indistinct truncated base and a hilum, smooth-walled, guttulate, budding occasionally observed. ***Chlamydospores*** (8.2–)10.1–16.0(–20.0) μm wide (*x̄* = 13.3 μm, *n* = 30), produced in aerial hyphae, mostly intercalary, solitary or in branched chains, globose to subglobose, pigmented, light to dark brown, smooth-, thick-walled, septate, 1–2 cells, constricted near the septa. ***Endoconidia*** absent.

Fermentation of glucose is negative. *D*-glucose, galactose, sorbose, *N*-acetyl glucosamine, ribose, xylose, *L*-arabinose, *D*-arabinose, *L*-rhamnose, sucrose (weak), maltose, *α*-*α* -trehalose, methyl-*α*-*D*-glucoside, cellobiose, salicin, melibiose, lactose, raffinose, melizitose, glycerol, erythritol, ribitol, glucitol, mannitol, galactitol, *myo*-inositol, *D*-glucono-1,5-lactone, *D*-gluconate, *D*-glucuronate, *D*-galacturonic acid (weak), *DL*-lactate (weak), succinate, citrate (weak), ethanol, and xylitol are assimilated, but inulin, soluble starch, and methanol are not assimilated. Ammonium sulfate, ethylamine hydrochloride, *L*-lysine, cadaverine, and creatine are assimilated as sole nitrogen sources. Potassium nitrate and sodium nitrite are not assimilated. After 3 weeks, light brown sediment formed in *D*-glucose, raffinose, and *D*-galacturonic acid, while dark brown sediment formed in *N*-acetylglucosamine, *D*-xylose, *L*-rhamnose, maltose, *α*-*α* -trehalose, methyl-*α*-*D*-glucoside, cellobiose, salicin, melibiose, lactose, *myo*-inositol, *D*-glucono-1,5-lactone, citrate, and xylitol. Black sediment formed in *D*-galactose, *L*-sorbose, *D*-arabinose, glycerol, erythritol, *D*-mannitol, and *D*-gluconate.

##### Cardinal temperatures for growth.

Minimum 10 °C, optimum in the range of 25 °C to 30 °C. No growth at 4 °C and 37 °C.

##### Additional strains examined.

THAILAND • Phayao Province, Mueang District, isolated from bougainvillea (*Bougainvillea hybrid*; *Nyctaginaceae*, *Caryophyllales*), Aug 2024, P. Kodchasee, C. Senwanna, J. Kumla and N. Suwannarach, living culture = SDBR-CMU859.

##### Habitat and distribution.

Flowers of *Bougainvillea hybrid*. Known only from northern Thailand.

##### Notes.

Phylogenetic analyses showed that *A. bougainvilleae* strains SDBR-CMU858 (ex-type) and SDBR-CMU859 formed a distinct monophyletic lineage that was sister to *A. catharanthi*, with 77% MLBS and 0.96 BIPP support (Fig. 2). The *rpb2*, *tef1*, and *tub* sequences of *A. bougainvilleae* differed from those of *A. catharanthi* by 4.96% (47/948 bp, including gaps), 9.29% (25/269 bp, including gaps), and 2.93% (12/409 bp, including gaps), respectively. Morphologically, colonies of *A. bougainvilleae* on PDA, MEA, and OA were grayish brown with a grayish-yellow margin, brownish gray with a grayish-brown to black margin, and pale orange with a light brown margin, respectively. In contrast, colonies of *A. catharanthi* were olive brown with grayish-yellow margins on PDA, grayish yellow mixed with olive brown and with grayish-yellow margins on MEA, and light gray with dark gray margins on OA. Although the conidial size ranges of *A. bougainvilleae* and *A. catharanthi* overlapped, the conidia of *A. bougainvilleae* became brown at maturity, whereas those of *A. catharanthi* remained hyaline. *Aureobasidium bougainvilleae* can be distinguished from *A. catharanthi* by its ability to assimilate *D*-xylose, *D*-arabinose, *L*-rhamnose, maltose, *α*,*α*-trehalose, cellobiose, melibiose, lactose, raffinose, ribitol, *D*-mannitol, *myo*-inositol, *D*-glucuronate, succinate, and ethanol, with sediment formation observed during growth on these carbon sources (Table 1). Thus, *A. bougainvilleae* is described here as a new species based on phylogenetic analysis and morphological characteristics.

#### Aureobasidium castaneae

Taxon classificationFungiDothidealesSaccotheciaceae

C.M. Tian & N. Jiang, Journal of Fungi 7 (1, no. 64): 11 (2021)

F00F7A81-EC1E-538D-B595-DC9118244A5F

838314

Fig. 8

##### Cultural characteristics.

After 14 days of incubation at 25 °C in the dark, colonies on PDA reaching 34–45 mm diam., surface flat, spreading, round with radiating, irregular margin, velvety, slimy, pale yellow (2A3), becoming olive brown (4E5) at the margin; on MEA reaching 28–35 mm diam., surface flat, spreading, round with radiating, irregular margin, velvety, slimy, dark brown (6F8) at center, becoming olive brown (4E5) to smoke brown (4E2) and white at the margin; on OA 32–45 mm diam., surface flat, spreading with irregular margin, velvety, slimy, pale yellow (2A3), becoming brownish gray (4E2), yellowish brown (5E8) at the margin.

**Figure 8.**
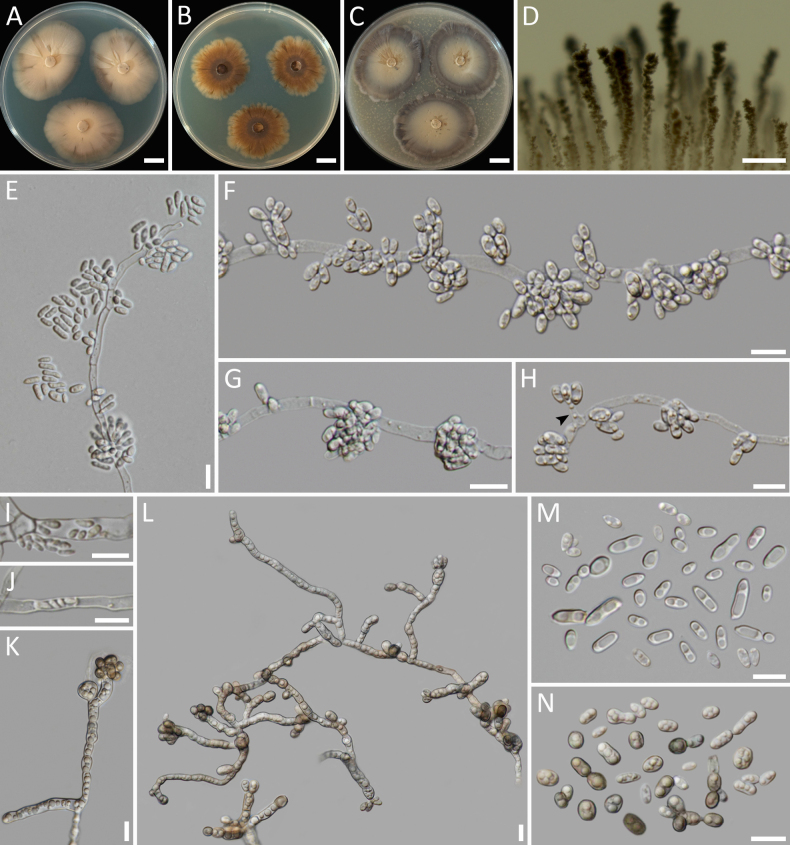
*Aureobasidium castaneae* (SDBR-CMU835). **A–C** colonies on PDA, MEA, and OA, respectively, after 14 d at 25 °C; **D** close-up of an immersed hyphal-tip colony on PDA after 14 d; **E–G** conidiogenous apparatus with hyaline conidia; **H** conidiogenous cell (arrow) integrated into a vegetative hypha with hyaline conidia; **I, J** endoconidia; **K, L** melanized hyphae with groups of hyaline conidia (HC) and melanized conidia (MC); **M** conidia; **N** hyaline and melanized conidia. Scale bars: 1 cm (**A–C**); 500 μm (**D**); 10 μm (**E–N**).

##### Description.

***Anamorph****in vitro* dematiaceous hyphomycetes. ***Vegetative hyphae*** 2.0–5.0(–6.6) μm wide, mostly hyaline, branched, septate, smooth, thin-walled, becoming light brown to brown, guttulate, thick-walled. ***Conidiogenous cells*** (4.8–)6.0–14.0(–16.0) × 2.5–6.0(–6.7) μm (*x̄* = 9.7 × 4.6 μm, *n* = 25), undifferentiated from hyphae, intercalary, rarely terminal, producing conidia percurrently from indistinct lateral denticles, giving rise to conidia that aggregate in a mucoid mass, or differentiated from hyphae, occasionally elongated, solitary, erect to lightly curved, hyaline becoming lightly melanized with age. ***Conidia*** (4.3–)5.0–9.0(–11.0) × (2.5–)3.0–4.5(–5.6) μm (*x̄* = 7 × 3.7 μm, *n* = 150), obovoid to ellipsoid, globose to subglobose, hyaline, becoming light brown to brown with age, aseptate, occasionally with an indistinct truncated base and a hilum, guttulate, smooth-walled, budding occasionally observed. ***Endoconidia*** (2.9–)3.4–5.6 × 1.6–2.5 μm (*x̄* = 4.0 × 1.9 μm, *n* = 25), obovoid to ellipsoid, hyaline, occasionally seen in intercalary hyphal cells.

##### Cardinal temperatures for growth.

Minimum 10 °C, optimum in the range of 25 °C to 30 °C. No growth at 4 °C and 37 °C.

##### Strains examined.

THAILAND • Chiang Mai, Mueang District, Suthep, isolated from American cassia flower (*Senna spectabilis*; *Fabaceae*, *Fabales*), Aug 2024, P. Kodchasee, C. Senwanna, J. Kumla and N. Suwannarach, living culture = SDBR-CMU835.

##### Habitat and distribution.

Leaf spots of *Castanea henryi* in China (Jiang et al. 2021); flowers of *Senna spectabilis* in Thailand (this study).

##### Notes.

Phylogenetic analysis showed that strain SDBR-CMU835 clustered with *A. castaneae* CFCC 54591 (ex-type) in the same clade, with strong statistical support (100% MLBS and 0.97 BIPP) (Fig. 2). The LSU and ITS sequences of strain SDBR-CMU835 showed 100% similarity to those of *A. castaneae* CFCC 54591. The morphology of strain SDBR-CMU835 is consistent with the description provided by Jiang et al. (2021). Therefore, this strain was identified as *A. castaneae*. In addition, an updated description of the melanized structures of the species is provided. Prior to this study, *A. castaneae* was reported on *Castanea henryi* in China (Jiang et al. 2021). This study represents the first report of *A. castaneae* in Thailand.

#### Aureobasidium catharanthi

Taxon classificationFungiDothidealesSaccotheciaceae

Senwanna, Kodchasee, J. Kumla & N. Suwannar. sp. nov.

968C2E17-24E8-555B-AF25-0B87C2437EAB

861959

Fig. 9

##### Etymology.

Refers to the host genus *Catharanthus*, from which this species was isolated.

**Figure 9.**
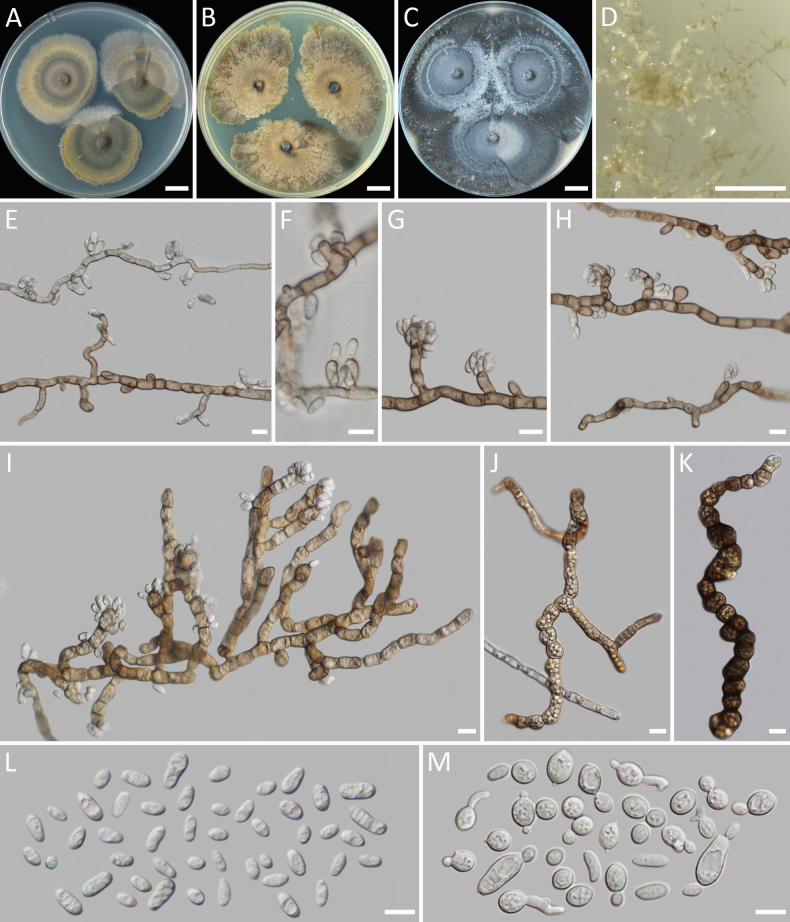
*Aureobasidium catharanthi* (SDBR-CMU842, ex-type). **A–C** colonies on PDA, MEA, and OA, respectively, after 14 d at 25 °C; **D** close-up of an immersed hyphal-tip colony on PDA after 14 d; **E–I** conidiogenous cells integrated into vegetative hyphae with conidia and conidiogenous apparatus; **J, K** melanized hyphae developing into chlamydospores; **L, M** conidia, some exhibiting yeast-like budding. Scale bars: 1 cm (**A–C**); 500 μm (**D**); 10 μm (**E–M**).

##### Type.

THAILAND • Phayao Province, Mueang District, isolated from vinca flower (*Catharanthus roseus*; *Apocynaceae*, *Gentianales*), Aug 2024, P. Kodchasee, C. Senwanna, J. Kumla and N. Suwannarach, ***holotype*** = CMUB40140 (preserved in metabolically inactive state), living culture (ex-type) = SDBR-CMU842 = GMBCC2503.

##### Cultural characteristics.

After 14 days of incubation at 25 °C in the dark, colonies on PDA reaching 36–44 mm diam., surface flat, spreading, round with radiating margin, velvety, olive brown (4E6), with grayish yellow (4B6) at the margin; on MEA reaching 55–60 mm diam., surface flat, spreading with lobate margin, velvety, shiny, grayish yellow (4B6), with olive brown (4E5) mixed with grayish yellow (4B6) at the margin; on OA 60–68 mm diam., surface flat, spreading with filiform, entire margin, velvety, slimy, light gray (3D1), with dark gray (3F1) at the margin.

##### Description.

***Anamorph****in vitro* dematiaceous hyphomycetes. ***Vegetative hyphae*** (3.9–)5.0–10.0(–16.0) μm, hyaline to brown, branched, septate, smooth, thin-walled, becoming light brown to brown with age, constricted at the septa, thick-walled. ***Conidiogenous cells*** (6.8–)9.3–13.0(–20.1) × 4.0–6.0 μm (*x̄* = 10.2 × 5.3 μm, *n* = 25), undifferentiated, intercalary, terminal, producing conidia percurrently from short lateral denticles, or differentiated on hyphae, occasionally elongated, solitary, light brown to brown. ***Conidia*** obovoid to ellipsoidal, or subglobose, hyaline, aseptate, occasionally with an indistinct truncated base and a hilum, smooth-walled, guttulate, (5.4–)6.8–11.5(–14.2) × (3.3–)4–5.3(–6.4) μm (*x̄* = 8.9 × 4.7 μm, *n* = 150), budding occasionally observed. ***Chlamydospores*** (8.0–)9.5–14.8(–18.1) μm wide (*x̄* = 12.2 μm, *n* = 30), frequent produced in aerial hyphae, mostly intercalary, solitary or in branched chains, globose to subglobose, light to dark brown, smooth-, thick-walled, septate, 1–2 cells, constricted near the septa. ***Endoconidia*** absent.

Fermentation of glucose is negative. *D*-glucose, galactose, sorbose, *N*-acetyl glucosamine (weak), ribose, xylose, *L*-arabinose, *D*-arabinose, *L*-rhamnose, sucrose, maltose, *α*-*α*-trehalose, methyl-*α*-*D*-glucoside (weak), cellobiose, salicin (weak), melibiose, lactose (weak), raffinose, melizitose, glycerol (weak), erythritol (weak), ribitol (weak), glucitol, mannitol, galactitol, *myo*-inositol, *D*-glucono-1,5-lactone (weak), *D*-gluconate, *D*-glucuronate, *D*-galacturonic acid (weak), *DL*-lactate (weak), succinate (weak), citrate (weak), ethanol, and xylitol are assimilated, but inulin, soluble starch, and methanol are not assimilated. Ammonium sulfate, ethylamine hydrochloride, *L*-lysine, cadaverine, and creatine are assimilated as sole nitrogen sources. Potassium nitrate and sodium nitrite are not assimilated. After 3 weeks, light brown sediment formed in *D*-glucose, *D*-galactose, *L*-sorbose, salicin, *D*-galacturonic acid, succinate, and citrate, while dark brown sediment formed in *N*-acetyl glucosamine, ribitol, *D*-glucono-1,5-lactone, *D*-gluconate, *D*-glucuronate, and xylitol. Black sediment formed in methyl-*α*-*D*-glucoside, glycerol, and erythritol, while white sediment observed in ethanol.

##### Cardinal temperatures for growth.

Minimum 10 °C, optimum in the range of 25 °C to 30 °C. No growth at 4 °C and 37 °C.

##### Additional strains examined.

THAILAND • Phayao Province, Mueang District, isolated from vinca flower (*Catharanthus roseus*; *Apocynaceae*, *Gentianales*), Aug 2024, P. Kodchasee, C. Senwanna, J. Kumla and N. Suwannarach, living culture = SDBR-CMU812.

##### Habitat and distribution.

Flowers of *Catharanthus roseus*. Known only from northern Thailand.

##### Notes.

Phylogenetic analyses showed that *A. catharanthi* formed a distinct monophyletic lineage and was sister to *A. bougainvilleae*, with 77% MLBS and 0.96 BIPP support (Fig. 2). The differences in sequence data, morphological characteristics, and physiological traits between *A. catharanthi* and *A. bougainvilleae* are described above. Therefore, *A. catharanthi* is introduced here as a new species.

#### Aureobasidium chiangmaiense

Taxon classificationFungiDothidealesSaccotheciaceae

Senwanna, Kodchasee, J. Kumla & N. Suwannar. sp. nov.

1796FCCE-A1E2-53AF-9024-DF2E4190AB69

 861960

Fig. 10

##### Etymology.

Refers to Chaing Mai Province, where the flowers containing the new fungus were collected.

**Figure 10.**
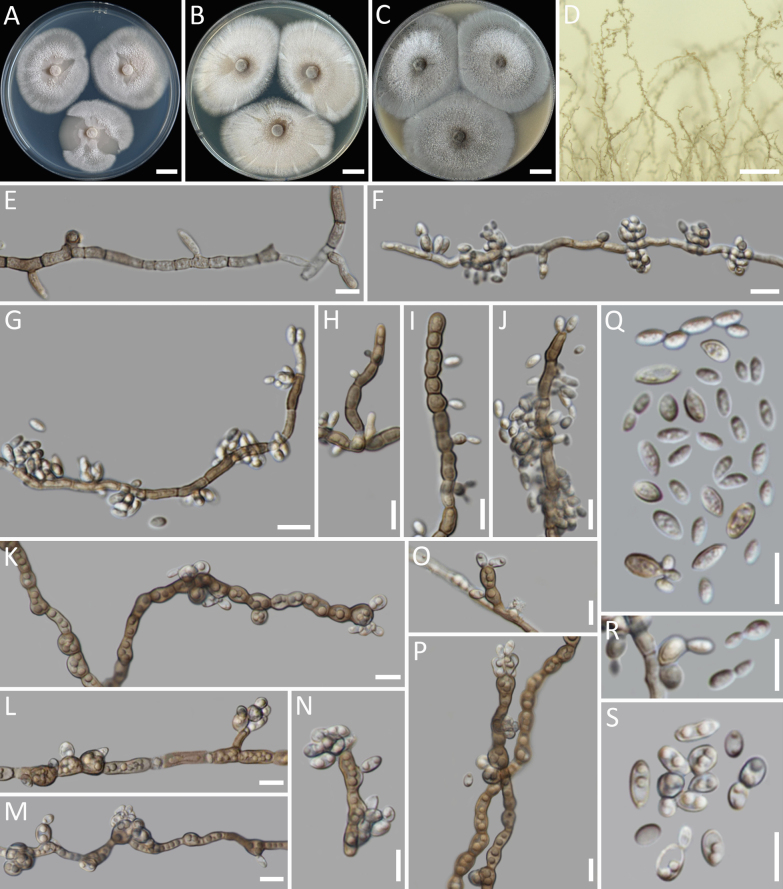
*Aureobasidium chiangmaiense* (SDBR-CMU819, ex-type). **A–C** colonies on PDA, MEA, and OA, respectively, after 14 d at 25 °C; **D** close-up of an immersed hyphal-tip colony on PDA after 14 d; **E–J** conidiogenous apparatus; **K–P** melanized hyphae developing into chlamydospores and producing hyaline conidia; **Q, R** conidia, some exhibiting yeast-like budding; **S** hyaline conidia produced from a melanized hypha. Scale bars: 1 cm (**A–C**); 500 μm (**D**); 10 μm (**E–S**).

##### Type.

THAILAND • Chiang Mai Province, Mueang District, Chang Phueak, isolated from oleander flower (*Nerium oleander*; *Apocynaceae*, *Gentianales*), Jul 2024, P. Kodchasee, C. Senwanna, J. Kumla and N. Suwannarach, ***holotype*** = CMUB40129 (preserved in metabolically inactive state), living culture (ex-type) = SDBR-CMU819 = GMBCC2497 = TBRC 21780.

##### Cultural characteristics.

After 14 days of incubation at 25 °C in the dark, colonies on PDA reaching 50–55 mm diam., surface round with slightly raised, filiform margin, velvety, white to orange gray (5B2), with light gray (3D1) at the margin; on MEA reaching 40–46 mm diam., surface flat, spreading with entire, filiform margin, velvety, grayish orange (6B5), with white to light gray (3D1) at the margin; on OA 42–47 mm diam., surface flat, woolly, spreading with filiform, entire margin, light gray (3D1) to brownish gray (4E2), with light gray (3D1) at the margin.

##### Description.

***Anamorph****in vitro* dematiaceous hyphomycetes. ***Vegetative hyphae*** 3.0–7.0(–8.8) μm wide, hyaline to brown, branched, septate, smooth, thin-walled, getting darker and thicker with age, light brown to brown, constricted at the septa, guttulate. ***Conidiogenous cells*** 9–10.5(–12.5) × 4.2–7.6 μm (*x̄* = 10.2 × 6.0 μm, *n* = 25), undifferentiated, intercalary, terminal, producing conidia percurrently from indistinct, short lateral denticles, giving rise to conidia that aggregate in a mucoid mass, or differentiated on hyphae, hyaline to light brown. ***Conidia*** obovoid to ellipsoidal, fusoid, or subglobose, hyaline to light brown, becoming brown with age, aseptate, occasionally with an indistinct truncated base and a hilum, smooth-walled, guttulate, (3.8–)5.2–7.9(–11.7) × (2.0–)2.8–5.0(–8.1) μm (*x̄* = 6.7 × 3.8 μm, *n* = 160), budding occasionally observed. ***Chlamydospores*** (5.5–)7–9(–11.5) (*x̄* = 8.1 μm, *n* = 25) µm wide, produced in aerial hyphae, mostly intercalary, in branched chains, globose to subglobose, light to dark brown, smooth-, thick-walled, septate, constricted near the septa. ***Endoconidia*** absent.

Fermentation of glucose is negative. *D*-glucose, galactose, sorbose, *N*-acetyl glucosamine, ribose (weak), xylose, *L*-arabinose, *D*-arabinose, *L*-rhamnose, sucrose, maltose, *α*-*α*-trehalose, methyl-*α*-*D*-glucoside, cellobiose, salicin (weak), melibiose (weak), lactose, raffinose (weak), melizitose, inulin (weak), glycerol, erythritol (weak), ribitol, glucitol, mannitol, galactitol, *myo*-inositol (weak), *D*-glucono-1,5-lactone, *D*-gluconate, *D*-glucuronate, *D*-galacturonic acid, succinate, citrate, ethanol (weak), and xylitol (weak) are assimilated, but soluble starch, *DL*-lactate, and methanol are not assimilated. Ammonium sulfate, ethylamine hydrochloride, *L*-lysine, cadaverine, and creatine are assimilated as sole nitrogen sources. Potassium nitrate and sodium nitrite are not assimilated. After 3 weeks, light brown sediment formed in *D*-glucose, *D*-galactose, *N*-acetyl glucosamine, *α*-*α*-trehalose, cellobiose, melibiose, melizitose, and glycerol, while dark brown sediment formed in *D*-xylose, salicin, raffinose, and *D*-glucono-1,5-lactone. Black sediment formed in erythritol.

##### Cardinal temperatures for growth.

Minimum 10 °C, optimum in the range of 25 °C to 30 °C. No growth at 4 °C and 37 °C.

##### Additional strains examined.

THAILAND • Chiang Mai Province, Mueang District, Chang Phueak, isolated from Persian gentian flower (*Exacum affine*; *Gentianaceae*, *Gentianales*), Jul 2024, P. Kodchasee, C. Senwanna, J. Kumla and N. Suwannarach, living culture = SDBR-CMU818.

##### Habitat and distribution.

Flowers of *Exacum affine* and *Nerium oleander*. Known only from northern Thailand.

##### Notes.

In the multilocus phylogenetic analyses, *A. chiangmaiense* SDBR-CMU818 and SDBR-CMU819 (ex-type) formed a distinct monophyletic lineage (100% MLBS and 1.00 BIPP) that was sister to *A. musti* and *A. zhengzhenxiangiae* (Fig. 2). The *rpb2* sequences of *A. chiangmaiense* showed 98.01% similarity to that of *A. musti* MCA7652, differing by 1.99% (18/906 bp, including gaps), and 96.33% similarity to that of *A. zhengzhenxiangiae* BRIP 76360a, differing by 3.67% (23/627 bp, including gaps). The sequences of *A. chiangmaiense* differed from those of *A. musti* SDBR-CMU825 and SDBR-CMU864 obtained in this study by 3.92% in *rpb2* (40/1,020 bp, including gaps), 9.38% in *tef1* (21/224 bp, including gaps), and 6.60% in *tub* (27/409 bp, including gaps). However, *A. chiangmaiense* is distinguishable from *A. musti* based on differences in colony characteristics. Colonies of *A. chiangmaiense* are white to orange gray with a light gray margin on PDA and grayish orange with a white to light gray margin on MEA, whereas *A. musti* forms reddish to purple colonies on PDA and yellow to orange colonies on MEA (Jumbam et al. 2025; this study). Moreover, *A. chiangmaiense* did not grow at 4 °C or 37 °C, whereas *A. musti* was able to grow at both temperatures. The conidia of *A. chiangmaiense* are wider (2.0–8.1 µm) than those of *A. musti* (0.5–4.8 µm) (Jumbam et al. 2025; this study). Nevertheless, morphological and colony comparisons with the type strain of *A. zhengzhenxiangiae* could not be made owing to the absence of available data (Tan et al. 2026b). Furthermore, the PHI test confirmed the genetic distinctiveness of *A. chiangmaiense*, *A. musti*, and *A. zhengzhenxiangiae* (Fig. 3). *Aureobasidium chiangmaiense* can be distinguished from *A. musti* by its ability to assimilate *L*-sorbose, *D*-arabinose, *L*-rhamnose, salicin, glycerol, erythritol, ribitol, *D*-mannitol, galactitol, *myo*-inositol, *D*-glucono-1,5-lactone, *D*-gluconate, succinate, ethanol, and xylitol (Jumbam et al. 2025). In addition, *A. chiangmaiense* differs from *A. musti* in its inability to assimilate potassium nitrate and sodium nitrite and its ability to assimilate creatine, whereas *A. musti* can assimilate potassium nitrate and sodium nitrite but not creatine (Jumbam et al. 2025). Based on the combined morphological, physiological, and molecular evidence, *A. chiangmaiense* is described herein as a new species.

#### Aureobasidium florale

Taxon classificationFungiDothidealesSaccotheciaceae

Senwanna, Kodchasee, J. Kumla & N. Suwannar. sp. nov.

A2B49BBB-9E20-58B3-A413-81F119CA88CA

861961

Fig. 11

##### Etymology.

Refers to its association with flowers, the substrate from which this species was isolated.

**Figure 11.**
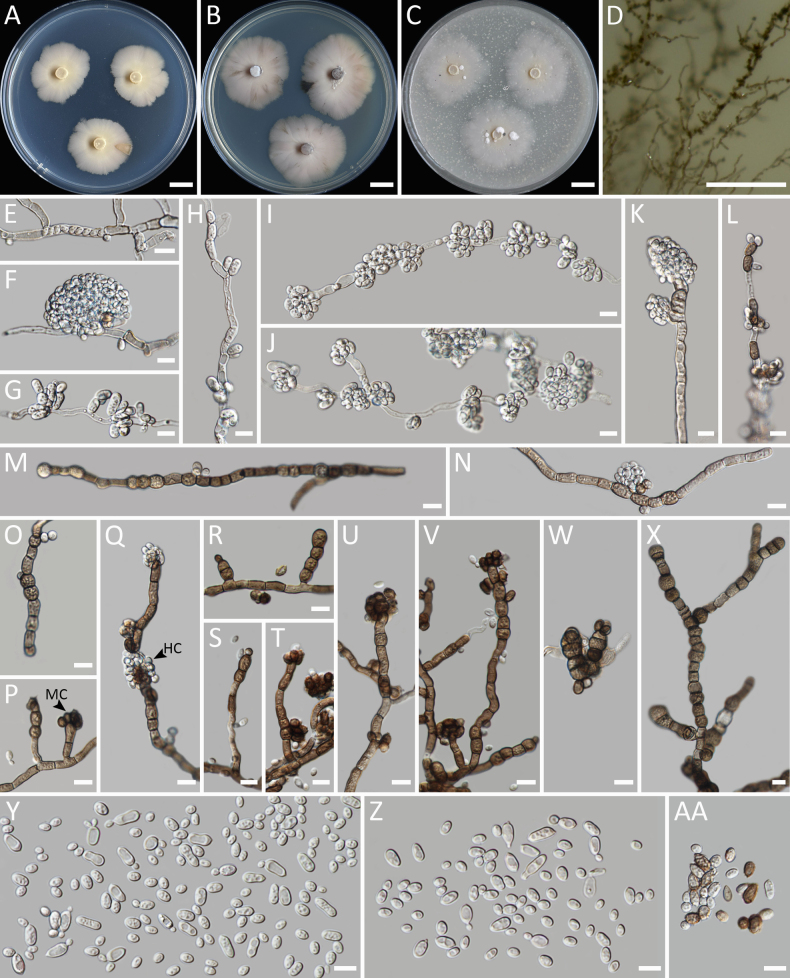
*Aureobasidium florale* (SDBR-CMU832, ex-type). **A–C** colonies on PDA, MEA, and OA, respectively, after 14 d at 25 °C; **D** close-up of an immersed hyphal-tip colony on PDA after 14 d; **E** endoconidia; **F–L** hyphae with groups of conidia and conidiogenous apparatus; **M–W** melanized hyphae with groups of hyaline conidia (HC) and melanized conidia (MC); **X** melanized hyphae developing into chlamydospores; **Y, Z** conidia, some exhibiting yeast-like budding; **AA** hyaline and melanized conidia from melanized hyphae. Scale bars: 1 cm (**A–C**); 500 μm (**D**); 10 μm (**E–Z, AA**).

##### Type.

THAILAND • Chiang Mai Province, Mueang District, Suthep, isolated from teak flower (*Tectona grandis*; *Lamiaceae*, *Lamiales*), Aug 2024, P. Kodchasee, C. Senwanna, J. Kumla and N. Suwannarach, ***holotype*** = CMUB40136 (preserved in metabolically inactive state), living culture (ex-type) = SDBR-CMU832 = GMBCC2501 = TBRC 21781.

##### Cultural characteristics.

After 14 days of incubation at 25 °C in the dark, colonies on PDA reaching 35–42 mm diam., surface flat, spreading with filiform, irregular margin, velvety, slimy, yellowish white (2A2); on MEA reaching 33–40 mm diam., surface flat, spreading with filiform, irregular margin, velvety, slimy, orange white (5A2); on OA 36–40 mm diam., surface flat, spreading with filiform, irregular margin, velvety, slimy, yellowish white (2A2) at center, light gray (3D1) at the margin.

##### Description.

***Anamorph****in vitro* dematiaceous hyphomycetes. ***Vegetative hyphae*** (2–)3.8–8.6(–14.9) μm wide, hyaline to dark brown, getting darker and thicker with age, branched, septate, smooth. ***Conidiogenous cells*** (7.5–)8.6–10.5(–12.5) × (5.5–)6.2–7.6 μm (*x̄* = 11.1 × 7.8 μm, *n* = 20), undifferentiated, intercalary, terminal, producing conidia percurrently from indistinct, short lateral denticles, giving rise to conidia that aggregate in a mucoid mass, or differentiated on hyphae, hyaline to light brown, occasionally elongated, solitary, becoming brown to dark brown with age. ***Conidia*** (3.8–)5.7–10.4(–13.6) × (2.6–)4–5.6(–7) μm (*x̄* = 7.8 × 4.8 μm, *n* = 200), obovoid to ellipsoidal, elongated, or globose to subglobose, hyaline becoming melanized with age, aseptate, occasionally with an indistinct truncated base and a hilum, guttulate, smooth-walled, budding occasionally observed. ***Chlamydospores*** 6.0–9.5(–13.6) (*x̄* = 8.5 μm, *n* = 25) µm wide, produced in aerial hyphae, intercalary, solitary or in branched chains, subglobose, pigmented, dark brown, smooth-, thick-walled, septate, constricted near the septa. ***Endoconidia*** (3.7–)4.3–5.4(–6) × 2.7–3.6(–4.5) μm (*x̄* = 4.8 × 3.3 μm, *n* = 25), obovoid to ellipsoidal, hyaline, occasionally seen in intercalary hyphal cells.

Fermentation of glucose is negative. *D*-glucose, galactose, sorbose (weak), *N*-acetyl glucosamine, ribose (weak), xylose, *L*-arabinose (weak), *D*-arabinose (weak), *L*-rhamnose, sucrose, maltose, *α*-*α*-trehalose (weak), methyl-*α*-*D*-glucoside (weak), cellobiose, salicin (weak), melibiose, lactose (weak), raffinose, melizitose, glycerol (weak), erythritol (weak), ribitol (weak), glucitol (weak), mannitol (weak), galactitol (weak), *myo*-inositol (weak), *D*-glucono-1,5-lactone (weak), *D*-gluconate (weak), *D*-glucuronate (weak), *D*-galacturonic acid (weak), succinate (weak), citrate, ethanol (weak), and xylitol (weak) are assimilated, but inulin, soluble starch, *DL*-lactate, and methanol are not assimilated. Ammonium sulfate, ethylamine hydrochloride, *L*-lysine, cadaverine, and creatine are assimilated as sole nitrogen sources. Potassium nitrate and sodium nitrite are not assimilated. After 3 weeks, light brown sediment formed in *D*-glucose, *D*-galactose, *L*-sorbose, *N*-acetyl glucosamine, *D*-arabinose, *L*-rhamnose, sucrose, maltose, *α*-*α*-trehalose, cellobiose, salicin, melibiose, lactose, raffinose, melizitose, erythritol, ribitol, *D*-glucitol, *D*-mannitol, *myo*-Inositol, *D*-glucuronate, *D*-galacturonic acid, succinate, citrate, and ethanol. Black sediment formed in *D*-ribose, *D*-xylose, *L*-arabinose, methyl-*α*-*D*-glucoside, glycerol, galactitol, *D*-glucono-1,5-lactone, *D*-gluconate, and xylitol.

##### Cardinal temperatures for growth.

Minimum 10 °C, optimum in the range of 25 °C to 30 °C. No growth at 4 °C and 37 °C.

##### Additional strains examined.

THAILAND • Chiang Mai Province, Mueang District, Suthep, isolated from teak flower (*Tectona grandis*; *Lamiaceae*, *Lamiales*), Aug 2024, P. Kodchasee, C. Senwanna, J. Kumla and N. Suwannarach, living culture = SDBR-CMU830; living culture = SDBR-CMU831; peregrina flower (*Jatropha integerrima*; *Euphorbiaceae*, *Malpighiales*), Aug 2024, P. Kodchasee, C. Senwanna, J. Kumla and N. Suwannarach, living culture = SDBR-CMU840; SDBR-CMU850; Chang Phueak, isolated from daisy flower (*Thymophylla tenuiloba*; *Asteraceae*, *Asterales*), Jul 2024, P. Kodchasee, C. Senwanna, J. Kumla and N. Suwannarach, living culture = SDBR-CMU862.

##### Habitat and distribution.

Flowers of *Jatropha integerrima*, *Tectona grandis*, and *Thymophylla tenuiloba*. Known only from northern Thailand.

##### Notes.

The multilocus phylogenetic tree showed that *A. florale* SDBR-CMU830, SDBR-CMU831, SDBR-CMU832 (ex-type), SDBR-CMU840, SDBR-CMU850, and SDBR-CMU862 formed a well-supported monophyletic lineage (99% MLBS and 0.98 BIPP) that was sister to *A. albui*, *A. castaneae*, *A. floricola*, and *A. lannaense* (Fig. 2). Additionally, the PHI test results (Fig. 4) indicated significant genetic differentiation, supporting the recognition of *A. florale* as a distinct species from *A. albui*, *A. castaneae*, *A. floricola*, and *A. lannaense*. Morphologically, colonies of *A. florale* on MEA are orange white, clearly differing from the grayish-brown colonies of *A. albui*, the olive-brown colonies of *A. castaneae*, the olive-brown to black colonies with a yellowish-white margin of *A. floricola*, and the yellowish-white and dark-gray to black colonies of *A. lannaense*. Although the micromorphology of *A. florale* is similar to that of *A. albui*, *A. castaneae*, *A. floricola*, and *A. lannaense*, these taxa can be distinguished based on multilocus phylogenetic analyses. Moreover, *A. florale* differs from *A. lannaense* by the presence of endoconidia. Furthermore, *A. florale* can be distinguished from *A. floricola* and *A. lannaense* by its inability to assimilate *DL*-lactate (Table 1). It also differs from *A. floricola* in its ability to assimilate methyl-α-*D*-glucoside. In addition, *A. florale* differs from *A. albui* in its ability to assimilate *D*-arabinose, *L*-rhamnose, salicin, glycerol, erythritol, ribitol, *myo*-inositol, ethanol, xylitol, and creatine (Jumbam et al. 2025). Moreover, *A. florale* did not produce pigmentation when grown in *L*-sorbose, erythritol, *D*-mannitol, or *D*-glucono-1,5-lactone, whereas *A. albui* produced pigmentation (Jumbam et al. 2025). Hence, *A. florale* is introduced as a new species based on both morphological characteristics and molecular data.

#### Aureobasidium floricola

Taxon classificationFungiDothidealesSaccotheciaceae

Senwanna, Kodchasee, J. Kumla & N. Suwannar. sp. nov.

AC98C17B-802A-598E-8220-222CFFB32BB6

861962

Fig. 12

##### Etymology.

Refers to its association with flowers, the substrate from which this species was isolated.

**Figure 12.**
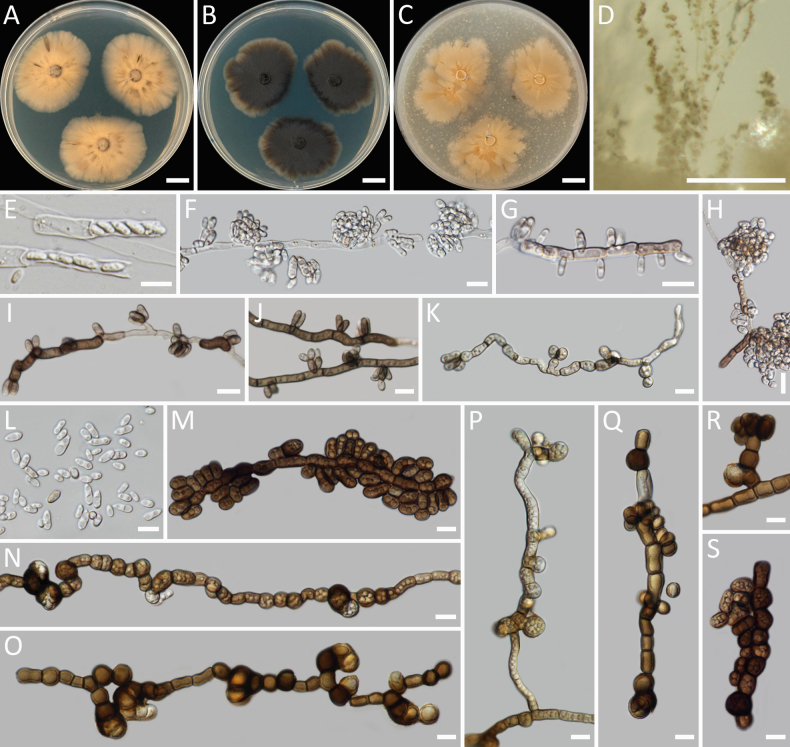
*Aureobasidium floricola* (SDBR-CMU829, ex-type). **A–C** colonies on PDA, MEA, and OA, respectively, after 14 d at 25 °C; **D** close-up of an immersed hyphal-tip colony on PDA after 14 d; **E** endoconidia; **F–H** hyphae with groups of conidia and conidiogenous apparatus; **I–K** conidiogenous cells integrated into vegetative hyphae with conidia; **L** conidia; **M** melanized conidia; **N–S** development of melanized hyphae, groups of melanized conidia, and chlamydospores. Scale bars: 1 cm (**A–C**); 500 μm (**D**); 10 μm (**E–S**).

##### Type.

THAILAND • Chiang Mai Province, Mueang District, Chang Phueak, isolated from kumquat flower (*Citrus japonica*; *Rutaceae*, *Sapindales*), Jul 2024, P. Kodchasee, C. Senwanna, J. Kumla and N. Suwannarach, ***holotype*** = CMUB40130 (preserved in metabolically inactive state), living culture (ex-type) = SDBR-CMU829 = GMBCC2500 = TBRC 21782.

##### Cultural characteristics.

After 14 days of incubation at 25 °C in the dark, colonies on PDA reaching 30–40 mm diam., surface flat, spreading, round with radiating margin, wrinkled, orange white (5A2), olive brown (4E5: tabby appearance), yellowish white (2A2) at margin; on MEA reaching 30–39 mm diam., surface flat, spreading, round with radiating margin, dry, velvety, olive brown (4E5) to black, yellowish white (2A2) at margin; on OA 26–34 mm diam., surface flat, spreading with irregular margin, yellowish white (2A2), orange white (5A2: tabby appearance).

##### Description.

***Anamorph****in vitro* dematiaceous hyphomycetes. ***Vegetative hyphae*** 2.7–7.5(–9.0) μm wide, hyaline to brown, branched, septate, smooth, thin-walled, becoming golden brown or dark brown, constricted at the septa, getting darker and thicker with age. ***Conidiogenous cells*** (4.0–)5.6–8.8(14.0) × (2.6–)3.5–6.1 μm (*x̄* = 7.2 × 4.2 μm, *n* = 25), undifferentiated, intercalary, terminal, producing conidia percurrently from indistinct, short lateral denticles, giving rise to conidia that aggregate in a mucoid mass, or differentiated on hyphae, hyaline to dark brown. ***Conidia*** (4.8–)7.3–11.0(–12.8) × (2.4–)3–7.1 μm (*x̄* = 8.9 × 4.7 μm, *n* = 200), obovoid to ellipsoidal, elongate, or subglobose, hyaline to light brown, becoming dark brown with age, aseptate, occasionally with an indistinct truncated base and a hilum, guttulate, smooth-walled. ***Chlamydospores*** (4.5–)7.5–16.7(–19.0) (*x̄* = 12.3 μm, *n* = 40) µm wide, produced in aerial hyphae, intercalary, in branched chains, subglobose to ellipsoidal, hyaline becoming light to dark brown, smooth-, thick-walled, septate, constricted near the septa. ***Endoconidia*** (3.9–)4.7–7.9 × 1.85–3.0(–3.7) μm (*x̄* = 6.0 × 2.7 μm, *n* = 27), obovoid to ellipsoidal, elongate, hyaline, occasionally seen in intercalary hyphal cells.

Fermentation of glucose is negative. *D*-glucose, galactose, sorbose, *N*-acetyl glucosamine, ribose, xylose, *L*-arabinose, *D*-arabinose (weak), *L*-rhamnose, sucrose, maltose, *α*-*α*-trehalose, cellobiose, salicin, melibiose, lactose, raffinose, melizitose, glycerol, erythritol, ribitol, glucitol, mannitol, galactitol, *myo*-inositol, *D*-glucono-1,5-lactone, *D*-gluconate, *D*-glucuronate, *D*-galacturonic acid, *DL*-lactate (weak), succinate, citrate, ethanol, and xylitol are assimilated, but methyl-*α*-*D*-glucoside, inulin, soluble starch, and methanol are not assimilated. Ammonium sulfate, ethylamine hydrochloride, *L*-lysine, cadaverine, and creatine are assimilated as sole nitrogen sources. Potassium nitrate and sodium nitrite are not assimilated. After 3 weeks, dark brown sediment formed in *D*-glucose, *D*-galactose, *L*-sorbose, *N*-acetyl glucosamine, *L*-rhamnose, maltose, *α*-*α*-trehalose, salicin, glycerol, *myo*-inositol, *D*-glucuronate, *D*-galacturonic acid, *DL*-lactate, and citrate. Black sediment formed in cellobiose, erythritol, *D*-glucono-1,5-lactone, *D*-gluconate, and succinate, while white sediment observed in *L*-arabinose.

##### Cardinal temperatures for growth.

Minimum 10 °C, optimum in the range of 25 °C to 30 °C. No growth at 4 °C and 37 °C.

##### Additional strains examined.

THAILAND • Phayao Province, Mueang District, isolated from Egyptian star cluster flower (*Pentas lanceolata*; *Rubiaceae*, *Gentianales*), Aug 2024, P. Kodchasee, C. Senwanna, J. Kumla and N. Suwannarach, living culture = SDBR-CMU853.

##### Habitat and distribution.

Flowers of *Citrus japonica* and *Pentas lanceolata*. Known only from northern Thailand.

##### Notes.

In the multilocus phylogenetic tree, *A. floricola* SDBR-CMU829 (ex-type) and SDBR-CMU853 clustered with *A. albui* SA57 and formed a distinct lineage separate from the clade of *A. albui* NRRL 61482 and SDBR-CMU866 (Fig. 2). The ITS, LSU, and *rpb2* sequences of *A. floricola* were identical (100%) to those of *A. albui* SA57; however, the ITS, LSU, and *rpb2* sequences showed 100%, 97.90%, and 96.97% similarity, respectively, to those of *A. albui* NRRL 61482 (ex-type), differing by 3.03% in *rpb2* (18/594 bp, including gaps). Additionally, the ITS, LSU, *rpb2*, *tef1*, and *tub* sequences of *A. floricola* showed 100%, 100%, 96.70%, 99.64%, and 100% similarity, respectively, to those of *A. albui* SDBR-CMU866 obtained in this study, differing by 3.30% in *rpb2* (31/939 bp, including gaps). However, further analyses of morphological characteristics, growth temperature profiles, and additional genetic loci are required to determine whether *A. albui* SA57 represents the same species as *A. floricola* or a distinct species, warranting further investigation in future studies. *Aureobasidium floricola* shares micromorphological features similar to those of *A. albui*; however, it is readily distinguished by its distinct colony morphology. Colonies of *A. floricola* are orange white to olive brown with a yellowish-white margin on PDA, olive brown to black with a yellowish-white margin on MEA, and yellowish white mixed with orange white on OA, whereas *A. albui* forms white to pale yellow colonies on PDA, greenish-brown to dark-brown colonies on MEA, and whitish-gray colonies on OA (Jumbam et al. 2025; this study). Furthermore, the PHI test revealed no significant genetic recombination between *A. floricola* and *A. albui* (Fig. 4), indicating that they represent distinct species. However, *A. floricola* differs from *A. albui* in its ability to assimilate *D*-arabinose, *L*-rhamnose, salicin, glycerol, erythritol, ribitol, *myo*-inositol, ethanol, xylitol, and creatine, while being unable to utilize potassium nitrate or sodium nitrite (Jumbam et al. 2025). Furthermore, *A. albui* produced pigmentation when grown on *L*-sorbose, erythritol, *D*-mannitol, or *D*-glucono-1,5-lactone, whereas *A. floricola* did not (Jumbam et al. 2025). Therefore, *A. floricola* is described herein as a new species.

#### Aureobasidium florigenum

Taxon classificationFungiDothidealesSaccotheciaceae

Senwanna, Kodchasee, J. Kumla & N. Suwannar. sp. nov.

70ACB130-2EB4-5CB1-A0D3-D62CA83D34B8

861963

Fig. 13

##### Etymology.

Refers to its origin from flowers, from which this species was isolated.

**Figure 13.**
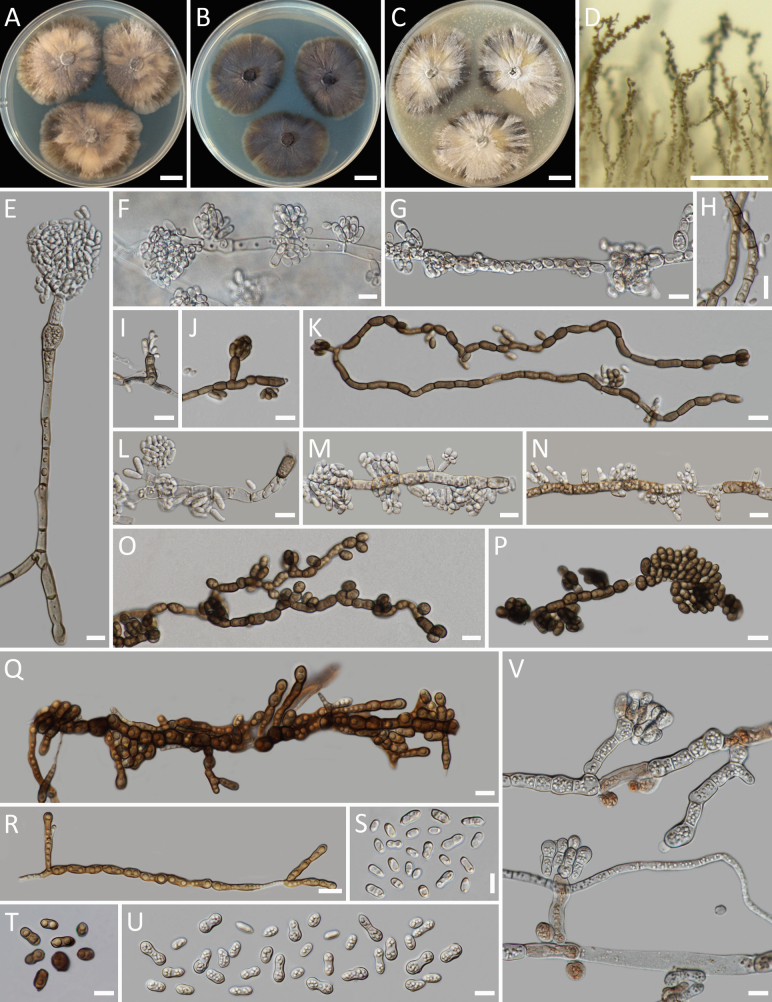
*Aureobasidium florigenum* (SDBR-CMU865, ex-type). **A–C** colonies on PDA, MEA, and OA, respectively, after 14 d at 25 °C; **D** close-up of an immersed hyphal-tip colony on PDA after 14 d; **E, F** hyphae with groups of conidia and conidiogenous apparatus; **G** endoconidia; **H** hyphae with multiple lateral pegs; **I–L** conidiogenous cells integrated into vegetative hyphae with conidia; **M, N** conidiogenous apparatus; **O–R** melanized hyphae with groups of melanized conidia and chlamydospores; **S** conidia; **T** melanized conidia; **U** hyaline conidia produced from a conidiophore-like structure; **V** conidiophore-like structure producing hyaline conidia. Scale bars: 1 cm (**A–C**); 500 μm (**D**); 10 μm (**E–V**).

##### Type.

THAILAND • Chiang Mai Province, Mueang District, Suthep, isolated from marigold flower (*Tagetes erecta*; *Asteraceae*, *Asterales*), Aug 2024, P. Kodchasee, C. Senwanna, J. Kumla and N. Suwannarach, ***holotype*** = CMUB40137 (preserved in metabolically inactive state), living culture (ex-type) = SDBR-CMU865 = GMBCC2512 = TBRC 21783.

##### Cultural characteristics.

After 14 days of incubation at 25 °C in the dark, colonies on PDA reaching 35–50 mm diam., surface flat, spreading, round with radiating margin, velvety, dry, gray (5C1) at center, orange white (5A2), olive brown (4E5: tabby appearance); on MEA reaching 30–42 mm diam., surface flat, spreading, round with radiating margin, wrinkled, velvety, black, olive brown (4E5: tabby appearance); on OA 37–49 mm diam., surface flat, spreading with irregular margin, yellowish white (2A2), light gray (2B1: tabby appearance), with brownish gray (4E2) at the margin.

##### Description.

***Anamorph****in vitro* dematiaceous hyphomycetes. ***Vegetative hyphae*** (2.7–)4.0–6.3(–10.0) μm wide, hyaline to brown, branched, septate, smooth, thin-walled, getting darker and thicker with age, constricted at the septa. ***Conidiophore-like structure*** differentiated on hyphae, (5.0–)7.0–10(–12.3) μm wide, hyaline to dark brown, branched, septate, smooth, thin-walled. ***Conidiogenous cells*** (5.0–)8.0–14.0 × 2.2–4.7(–5.8) μm (*x̄* = 10.1 × 4.1 μm, *n* = 25), undifferentiated, intercalary, terminal, producing conidia percurrently from indistinct, short lateral denticles, giving rise to conidia that aggregate in a mucoid mass, or differentiated on hyphae, hyaline becoming dark brown with age. ***Conidia*** ellipsoidal to dumbbell shaped, hyaline, becoming light to dark brown with age, aseptate, occasionally with an indistinct truncated base and a hilum, smooth-walled, guttulate, (4.1–)6.3–13.0(–19.2) × (2.2–)3.4–6.3(–10) μm (*x̄* = 9.1 × 4.8 μm, *n* = 300). ***Chlamydospores*** (7.4–)10.5–17.0 (*x̄* = 13.0 μm, *n* = 25) µm wide, produced in aerial hyphae, mostly intercalary, solitary or in branched chains, subglobose to ellipsoidal, dark brown, smooth-, thick-walled, septate, constricted near the septa. ***Endoconidia*** (4.5–)5.2–8.0(–9.3) × (3.2–)3.5–4.3(–4.9) μm (*x̄* = 6.7 × 4.1 μm, *n* = 25), obovoid to ellipsoidal, hyaline, occasionally seen in intercalary hyphal cells.

Fermentation of glucose is negative. *D*-glucose, galactose, sorbose, *N*-acetyl glucosamine, ribose, xylose, *L*-arabinose, *D*-arabinose, *L*-rhamnose, sucrose, maltose, *α*-*α*-trehalose, methyl-*α*-*D*-glucoside, cellobiose, salicin, melibiose, lactose, raffinose, melizitose, glycerol, erythritol, ribitol, glucitol, mannitol, galactitol, *myo*-inositol, *D*-glucono-1,5-lactone, *D*-gluconate (weak), *D*-glucuronate, *D*-galacturonic acid, *DL*-lactate, succinate, citrate, ethanol, and xylitol are assimilated, but inulin, soluble starch, and methanol are not assimilated. Ammonium sulfate, ethylamine hydrochloride, *L*-lysine, cadaverine, and creatine are assimilated as sole nitrogen sources. Potassium nitrate and sodium nitrite are not assimilated. After 3 weeks, dark brown sediment formed in *D*-glucose, methyl-*α*-*D*-glucoside, erythritol, *myo*-inositol, and *D*-glucuronate. Black sediment formed in *L*-sorbose, *D*-gluconate, and ethanol, while white sediment observed in *D*-galactose, *D*-ribose, *D*-xylose, *L*-arabinose, *L*-rhamnose, and galactitol.

##### Cardinal temperatures for growth.

Minimum 10 °C, optimum in the range of 25 °C to 30 °C. No growth at 4 °C and 37 °C.

##### Additional strains examined.

THAILAND • Chiang Mai Province, Mueang District, Suthep, isolated from yellow elder flower (*Tecoma stans*; *Bignoniaceae*, *Lamiales*), Jul 2024, P. Kodchasee, C. Senwanna, J. Kumla and N. Suwannarach, living culture = SDBR-CMU828.

##### Habitat and distribution.

Flowers of *Tagetes erecta* and *Tecoma stans*. Known only from northern Thailand.

##### Notes.

Phylogenetically, *A. florigenum* SDBR-CMU865 (ex-type) and SDBR-CMU828 formed a well-supported monophyletic lineage (100% MLBS and 0.99 BIPP) that was sister to *A. anthicola* (Fig. 2). Furthermore, the PHI test confirmed the genetic distinctiveness of *A. florigenum* and *A. anthicola* (Fig. 4). The molecular, morphological, and physiological differences between *A. florigenum* and *A. anthicola* are described above. Therefore, *A. florigenum* is described herein as a new species.

#### Aureobasidium kwanphayaoense

Taxon classificationFungiDothidealesSaccotheciaceae

Senwanna, Kodchasee, J. Kumla & N. Suwannar. sp. nov.

8759B758-E030-501F-8B7B-0689D3357E3F

861964

Fig. 14

##### Etymology.

Refers to Kwan Phayao (Phayao Lake) in Mueang Phayao District, Phayao Province, Thailand, where the flowers containing the new fungus were collected.

**Figure 14.**
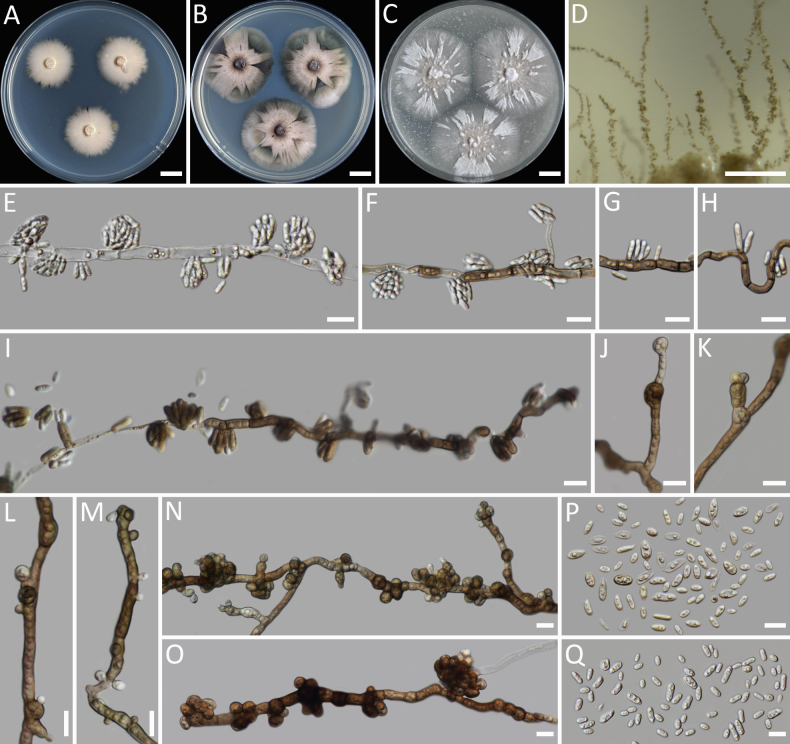
*Aureobasidium kwanphayaoense* (SDBR-CMU849, ex-type). **A–C** colonies on PDA, MEA, and OA, respectively, after 14 d at 25 °C; **D** close-up of an immersed hyphal-tip colony on PDA after 14 d; **E–I** conidiogenous cells integrated into vegetative hyphae with hyaline conidia; **J–O** melanized hyphae with groups of melanized conidia and chlamydospores; **P, Q** conidia. Scale bars: 1 cm (**A–C**); 500 μm (**D**); 10 μm (**E–Q**).

##### Type.

THAILAND • Phayao Province, Mueang District, isolated from chaya spinach flower (*Cnidoscolus aconitifolius*; *Euphorbiaceae*, *Malpighiales*), Aug 2024, P. Kodchasee, C. Senwanna, J. Kumla and N. Suwannarach, ***holotype*** = CMUB40139 (preserved in metabolically inactive state), living culture (ex-type) = SDBR-CMU849 = GMBCC2506 = TBRC 21784.

##### Cultural characteristics.

After 14 days of incubation at 25 °C in the dark, colonies on PDA reaching 28–30 mm diam., surface flat, spreading, round with radiating margin, velvety, slimy, yellowish white (2A2); on MEA reaching 40–48 mm diam., surface flat, spreading, round with radiating margin, velvety, orange gray (5B2) at center, grayish brown (7E3) at the margin; on OA 42–47 mm diam., surface flat, spreading with filiform, entire margin, powdery, fluffy, light gray (3D1).

##### Description.

***Anamorph****in vitro* dematiaceous hyphomycetes. ***Vegetative hyphae*** 2.8–7.2(–9.0) μm wide, hyaline to dark brown, branched, septate, smooth, thin-walled, getting darker and thicker with age, constricted at the septa. ***Conidiogenous cells*** 10.0–11.7 × 3.0–3.8 μm (*x̄* = 11 × 3.8 μm, *n* = 25), undifferentiated, intercalary, rarely terminal, producing conidia percurrently from short lateral denticles, giving rise to conidia that aggregate in a mucoid mass, or differentiated on hyphae, hyaline, becoming dark brown. ***Conidia*** obovoid to ellipsoidal, elongated, or globose to subglobose, hyaline to brown, becoming dark brown with age, aseptate, occasionally with an indistinct truncated base and a hilum, guttulate, smooth-walled, (4.0–)5.8–11.0(–14.2) × 2.0–4.4(–7.0) μm (*x̄* = 8.4 × 3.5 μm, *n* = 150). ***Chlamydospores*** (6.0–)7.0–14.5(–17.6) (*x̄* = 8.9 μm, *n* = 25) µm wide, produced in aerial hyphae, mostly intercalary, solitary or in branched chains, globose to subglobose, dark brown, smooth-, thick-walled, septate, constricted near the septa. ***Endoconidia*** absent.

Fermentation of glucose is negative. *D*-glucose, galactose, sorbose, *N*-acetyl glucosamine, ribose, xylose, *L*-arabinose, *D*-arabinose, *L*-rhamnose, sucrose, maltose, *α*-*α*-trehalose, methyl-*α*-*D*-glucoside, cellobiose, salicin, melibiose, lactose, raffinose, melizitose, glycerol, erythritol, ribitol, glucitol, mannitol, galactitol, *myo*-inositol, *D*-glucono-1,5-lactone, *D*-gluconate, *D*-glucuronate, *D*-galacturonic acid, *DL*-lactate, succinate, citrate, ethanol, and xylitol are assimilated, but inulin, soluble starch, and methanol are not assimilated. Ammonium sulfate, ethylamine hydrochloride, *L*-lysine, cadaverine, and creatine are assimilated as sole nitrogen sources. Potassium nitrate and sodium nitrite are not assimilated. After 3 weeks, dark brown sediment formed in L-sorbose, salicin, erythritol. Black sediment formed in *D*-glucose, *D*-arabinose, methyl-*α*-*D*-glucoside, raffinose, glycerol, *D*-glucitol, *myo*-inositol, *D*-glucono-1,5-lactone, *D*-gluconate, and ethanol.

##### Cardinal temperatures for growth.

Minimum 10 °C, optimum in the range of 25 °C to 30 °C. No growth at 4 °C and 37 °C.

##### Additional strains examined.

THAILAND • Phayao Province, Mueang District isolated from peregrina flower (*Jatropha integerrima*; *Euphorbiaceae*, *Malpighiales*), Aug 2024, P. Kodchasee, C. Senwanna, J. Kumla and N. Suwannarach, living culture = SDBR-CMU839; isolated from holy basil flower (*Ocimum tenuiflorum*; *Lamiaceae*; *Lamiales*), Aug 2024, P. Kodchasee, C. Senwanna, J. Kumla and N. Suwannarach, living culture = SDBR-CMU844; isolated from purslane flower (*Portulaca grandiflora*; *Portulacaceae*, *Caryophyllales*), Aug 2024, P. Kodchasee, C. Senwanna, J. Kumla and N. Suwannarach, living culture = SDBR-CMU860.

##### Habitat and distribution.

Flowers of *Cnidoscolus aconitifolius*, *Jatropha integerrima*, *Ocimum tenuiflorum*, and *Portulaca grandiflora*. Known only from northern Thailand.

##### Notes.

The multilocus phylogenetic tree (Fig. 2) showed that *A. kwanphayaoense* formed a distinct clade, supported by 100% MLBS and 1.0 BIPP, and was separated from other *Aureobasidium* species in the *A. thailandense* group. *Aureobasidium kwanphayaoense* formed a sister clade to *A. albui*, *A. anthicola*, *A. castaneae*, *A. florale*, *A. floricola*, *A. florigenum*, *A. lannaense*, and *A. vanuatuense*. Morphologically, the colony characteristics of *A. kwanphayaoense* differed from those of most phylogenetically related sister taxa but were similar to those of *A. albui*, *A. florale*, and *A. lannaense*. However, endoconidia are absent in *A. kwanphayaoense*, whereas they are present in *A. albui* and *A. florale*. In addition, *A. kwanphayaoense* can be distinguished from these taxa based on multilocus phylogenetic analyses and nucleotide differences in the *rpb2* and *tef1* loci (Table 2). Moreover, the results of the PHI test further supported the genetic distinctiveness of *A. kwanphayaoense* within the *A. thailandense* group (Fig. 4). Furthermore, *A. kwanphayaoense* can be distinguished from *A. anthicola*, *A. florale*, *A. floricola*, *A. lannaense*, and *A. florigenum* by its characteristic pattern of sediment formation in carbon assimilation tests (Table 1). *Aureobasidium kwanphayaoense* can be distinguished from *A. anthicola* by the absence of pigmentation on salicin, *D*-glucono-1,5-lactone, and *D*-gluconate, which is characteristic of *A. anthicola* (Table 1). In addition, *A. kwanphayaoense* also differs from *A. floricola* in its ability to assimilate methyl-α-*D*-glucoside. *Aureobasidium albui* and *A. vanuatuense* are clearly separated from *A. kwanphayaoense* by their inability to assimilate *L*-arabinose, *L*-rhamnose, salicin, glycerol, ribitol, *myo*-inositol, ethanol, and xylitol as carbon sources, as well as creatine as a nitrogen source (Jumbam et al. 2025). Based on the combined morphological, physiological, and molecular evidence, *A. kwanphayaoense* is described herein as a new species.

**Table 2.** Comparison of ITS, LSU, *rpb2*, *tef1*, and *tub* nucleotides between *Aureobasidium kwanphayaoense* and related species. An asterisk (*) indicates the type strain.

***A. kwanphayaoense* vs**.	**Strain**	**Sequence region (base pairs)**				
		**ITS**	**LSU**	***rpb2***	***tef1***	***tub***
*A. albui*	NRRL 61482*	2/561 (0.3%)	21/815 (2.6%)	2/594 (0.3%)	–	–
	SDBR-CMU866	2/561 (0.3%)	1/849 (0.1%)	8/921 (0.9%)	25/266 (9.4%)	0/399 (0%)
*A. anthicola*	SDBR-CMU851*	2/561 (0.3%)	2/849 (0.2%)	49/921 (5.3%)	2/266 (0.8%)	0/399 (0%)
*A. castaneae*	CFCC 54591*	5/496 (1.0%)	1/587 (0.2%)	–	–	–
	SDBR-CMU835	6/561 (1.1%)	1/849 (0.1%)	32/921 (3.8%)	26/266 (9.8%)	5/399(1.3%)
*A. florale*	SDBR-CMU832*	2/561 (0.3%)	1/849 (0.1%)	27/921 (2.9%)	0/266 (0%)	3/399(0.8%)
*A. floricola*	SDBR-CMU829*	2/561 (0.3%)	1/849 (0.1%)	29/921 (3.1%)	26/266 (9.8%)	0/399 (0%)
*A. florigenum*	SDBR-CMU865*	0/561 (0%)	0/849 (0%)	34/921 (3.7%)	1/266 (0.4%)	0/399 (0%)
*A. lannaense*	SDBR-CMU863*	2/561 (0.3%)	1/849 (0.1%)	30/921 (3.3%)	14/266 (5.3%)	3/399(0.8%)
*A. vanuatuense*	NRRL 64180*	3/561 (0.5%)	0/849 (0%)	15/739 (2.0%)	–	–

“–” indicates no result due to unavailable sequence data.

#### Aureobasidium lannaense

Taxon classificationFungiDothidealesSaccotheciaceae

Senwanna, Kodchasee, J. Kumla & N. Suwannar. sp. nov.

0ABD8DE0-E987-5569-B8E1-3907BABE4BB8

 861965

Fig. 15

##### Etymology.

Refers to the Kingdom of Lanna, the historic name of northern Thailand, where the flowers containing the new fungus were collected.

**Figure 15.**
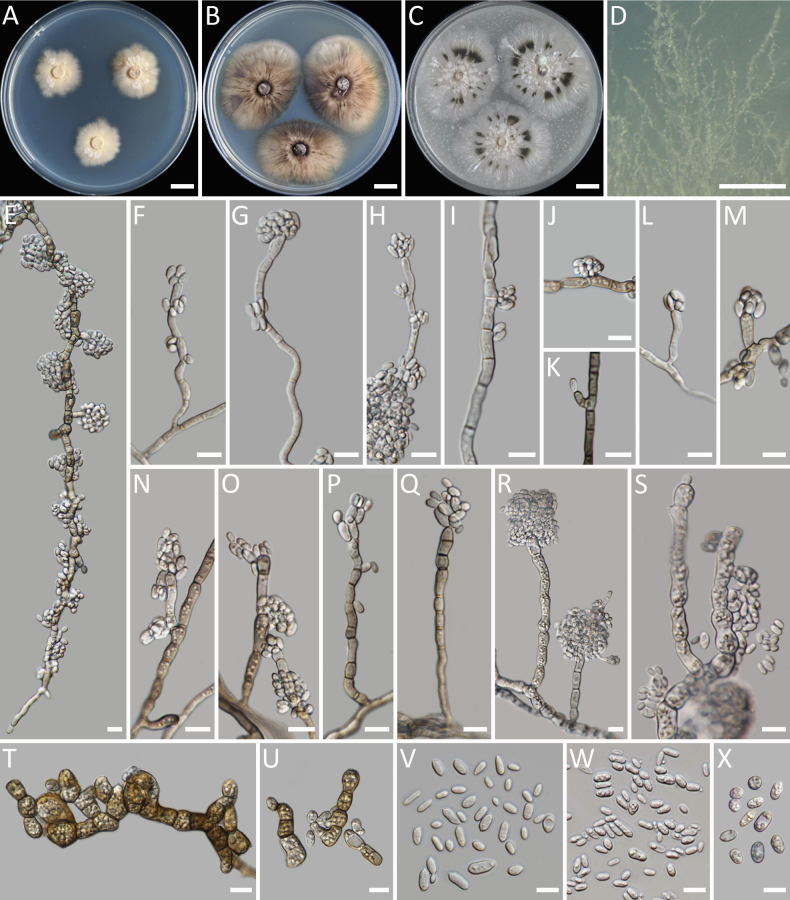
*Aureobasidium lannaense* (SDBR-CMU863, ex-type). **A–C** colonies on PDA, MEA, and OA, respectively, after 14 d at 25 °C; **D** close-up of an immersed hyphal-tip colony on PDA after 14 d; **E–J** hyphae with groups of conidia; **K–O** conidiogenous cells integrated into vegetative hyphae with conidia; **P, Q** conidiogenous apparatus; **R, S** development of melanized hyphae with groups of conidia; **T, U** melanized hyphae with chlamydospores and melanized conidia; **V, W** conidia, some exhibiting yeast-like budding; **X** hyaline and melanized conidia from melanized hyphae. Scale bars: 1 cm (**A–C**); 500 μm (**D**); 10 μm (**E–X**).

##### Type.

THAILAND • Chiang Mai Province, Mueang District, Suthep, isolated from flosreginae flower (*Lagerstroemia speciosa*; *Lythraceae*, *Myrtales*), Aug 2024, P. Kodchasee, C. Senwanna, J. Kumla and N. Suwannarach, ***holotype*** = CMUB40135 (preserved in metabolically inactive state), living culture (ex-type) = SDBR-CMU863 = GMBCC2511 = TBRC 21785.

##### Cultural characteristics.

After 14 days of incubation at 25 °C in the dark, colonies on PDA reaching 25–28 mm diam., surface flat, spreading with filiform, irregular margin, velvety, fluffy, slimy, yellowish white (2A2); on MEA reaching 45–54 mm diam., surface flat, spreading, round with radiating margin, velvety, yellowish white (2A2), dark gray (3F1) to black (tabby appearance); on OA 42–47 mm diam., surface flat, spreading with irregular margin, powdery, light gray (4D1) and olive brown (4E5: tabby appearance), with light gray (4D1) at the margin.

##### Description.

***Anamorph****in vitro* dematiaceous hyphomycetes. ***Vegetative hyphae*** 3.0–10.0(–16.0) μm wide, hyaline to dark brown, branched, septate, smooth, thin-walled, becoming light brown to golden brown, constricted at the septa, thick-walled. ***Conidiogenous cells*** (5.0–)7.5–15.0(–21.0) × (3.0–)4.2–7.0(–9.0) μm (*x̄* = 11.8 × 5.8 μm, *n* = 25), undifferentiated, intercalary, rarely terminal, producing conidia percurrently from indistinct, short lateral denticles, giving rise to conidia that aggregate in a mucoid mass, or differentiated on hyphae, hyaline to dark brown. ***Conidia*** oval, obovoid to ellipsoidal, elongated, or globose to subglobose, hyaline, becoming light brown with age, aseptate, occasionally with an indistinct truncated base and a hilum, guttulate, smooth-walled, (4.0–)5.4–9.0(–11.0) × 2.5–4.7(–6.3) μm (*x̄* = 7.0 × 3.8 μm, *n* = 200), budding occasionally observed. ***Chlamydospores*** (7.4–)8.7–11(–14.9) (*x̄* = 10.0 μm, *n* = 25) µm wide, produced in aerial hyphae, mostly intercalary, solitary or in branched chains, globose to subglobose or ellipsoidal, hyaline becoming golden brown, smooth-, thick-walled, septate, constricted near the septa. ***Endoconidia*** absent.

Fermentation of glucose is negative. *D*-glucose, galactose, sorbose, *N*-acetyl glucosamine, ribose, xylose, *L*-arabinose, *D*-arabinose (weak), *L*-rhamnose, sucrose, maltose, *α*-*α*-trehalose, methyl-*α*-*D*-glucoside (weak), cellobiose, salicin, melibiose, lactose, raffinose, melizitose, glycerol, erythritol, ribitol, glucitol, mannitol, galactitol, *myo*-inositol, *D*-glucono-1,5-lactone, *D*-gluconate, *D*-glucuronate, *D*-galacturonic acid, *DL*-lactate (weak), succinate, citrate, ethanol, and xylitol (weak) are assimilated, but inulin, soluble starch, and methanol are not assimilated. Ammonium sulfate, ethylamine hydrochloride, *L*-lysine, cadaverine, and creatine are assimilated as sole nitrogen sources. Potassium nitrate and sodium nitrite are not assimilated. After 3 weeks, black sediment formed in *D*-glucose, *D*-arabinose, methyl-*α*-*D*-glucoside, and *D*-gluconate, while white sediment observed in lactose.

##### Cardinal temperatures for growth.

Minimum 10 °C, optimum in the range of 25 °C to 30 °C. No growth at 4 °C and 37 °C.

##### Additional strains examined.

THAILAND • Chiang Mai Province, Mueang District, Suthep, isolated from Indian head ginger flower (*Cheilocostus speciosus*; *Costaceae*, *Zingiberales*), Sep 2024, P. Kodchasee, C. Senwanna, J. Kumla and N. Suwannarach, living culture = SDBR-CMU833.

##### Habitat and distribution.

Flowers of *Cheilocostus speciosus* and *Lagerstroemia speciosa*. Known only from northern Thailand.

##### Notes.

In the multilocus phylogenetic tree, *A. lannaense* SDBR-CMU833 and SDBR-CMU863 (ex-type) formed a distinct monophyletic lineage (100% MLBS and 0.99 BIPP) that was sister to *A. albui* and *A. floricola* (Fig. 2). Comparisons of nucleotide sequences showed that the *rpb2* sequence of *A. lannaense* showed 96.97% similarity to that of *A. albui* NRRL 61482 (ex-type) (18/594 bp, including gaps) and 96.59% similarity to that of *A. albui* SDBR-CMU866 (32/921 bp, including gaps), whereas the *tef1* sequence showed 93.93% similarity to that of *A. albui* SDBR-CMU866 (17/266 bp, including gaps). The *tef1* sequence of *A. lannaense* showed 93.51% similarity to that of *A. floricola* SDBR-CMU829 (ex-type) (18/266 bp, including gaps). Morphological comparison revealed distinct colony characteristics among the three species. On MEA, *A. lannaense* produced yellowish-white to dark-gray to black colonies, *A. floricola* produced olive-brown to black colonies with yellowish-white margins, and *A. albui* produced yellowish-brown to smoke-brown colonies with white margins. On OA, *A. lannaense* formed light-gray to olive-brown colonies with light-gray margins, differing from *A. floricola*, which formed yellowish-white to orange-white colonies, and *A. albui*, which formed whitish-gray colonies with light-gray margins. *Aureobasidium lannaense* can be distinguished from *A. albui* and *A. floricola* by its lack of endoconidium production. Growth at 4 °C was observed only in *A. albui*, whereas both *A. lannaense* and *A. floricola* did not grow at this temperature. Moreover, the PHI test further supported the genetic distinctiveness of *A. lannaense*, *A. albui*, and *A. floricola*, confirming that they represent distinct species (Fig. 4). *Aureobasidium lannaense* can be distinguished from *A. floricola* by its ability to assimilate methyl-*α*-*D*-glucoside (Table 1). In addition, *A. lannaense* differs from *A. albui* in its ability to assimilate *D*-arabinose, *L*-rhamnose, salicin, glycerol, erythritol, ribitol, *myo*-inositol, ethanol, and xylitol as carbon sources, as well as creatine as a nitrogen source (Jumbam et al. 2025). Therefore, *A. lannaense* is described herein as a new species.

#### Aureobasidium melanogenum

Taxon classificationFungiDothidealesSaccotheciaceae

(Herm.-Nijh.) Zalar, Gostincar & Gunde-Cim., BMC Genomics 15: 16 (2014)

A370677C-6044-52A7-B2E0-2E8FB8C780B1

807698

Fig. 16

##### Cultural characteristics.

After 14 days of incubation at 25 °C in the dark, colonies on PDA reaching 45–65 mm diam., surface flat, spreading with entire margin, zonate, orange white (5A2), with brownish gray (4E2) to olive brown (4E6) at the margin; on MEA reaching 65–70 mm diam., surface flat, spreading with entire margin, velvety, zonate, dark brown (6F8) at center, becoming brownish orange (6C8), with brownish gray (4E2) and white at the margin; on OA 60–75 mm diam., surface flat, spreading with irregular margin, velvety, grayish orange (6B5), with brownish gray (4E2) at the margin.

**Figure 16.**
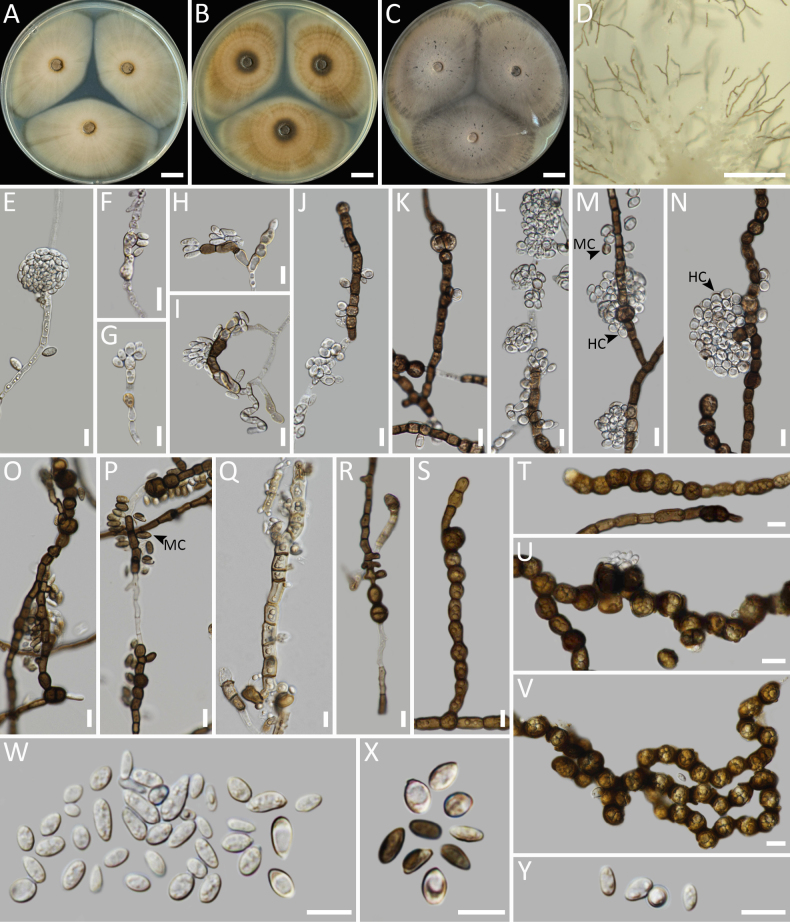
*Aureobasidium melanogenum* (SDBR-CMU855). **A–C** colonies on PDA, MEA, and OA, respectively, after 14 d at 25 °C; **D** close-up of an immersed hyphal-tip colony on PDA after 14 d; **E** hyphae with groups of hyaline conidia; **F–I***Hormonema*-like conidiogenous cells with hyaline conidia; **J–Q** melanized hyphae with groups of hyaline conidia (HC) and melanized conidia (MC); **R–T** melanized hyphae developing into chlamydospores; **U, V, Y** chlamydospores budding to produce hyaline conidia; **W, X** conidia. Scale bars: 1 cm (**A–C**); 500 μm (**D**); 10 μm (**E–Y**).

##### Description.

***Anamorph****in vitro* dematiaceous hyphomycetes. ***Vegetative hyphae*** (2.3–)3.0–5.5(–8.0) μm wide, mostly hyaline, light brown to brown, branched, septate, constricted at the septa, smooth, thin-walled, getting darker and thicker with age. ***Conidiogenous cells*** (3.8–)5.3–11.0(–12.5) × (3.2–)4.0–7.5(–8.3) μm (*x̄* = 8.7 × 5.3 μm, *n* = 25), undifferentiated, intercalary, rarely terminal, producing conidia percurrently from indistinct lateral denticles, giving rise to conidia that aggregate in a mucoid mass, or differentiated from hyphae, hyaline to lightly melanized. ***Conidia*** (3.6–)5.5–8.5(–11.4) × (2.0–)2.8–6.0 μm (*x̄* = 7.1 × 4.1 μm, *n* = 150), obovoid to ellipsoid, or subglobose, hyaline, becoming lightly melanized with age, aseptate, occasionally with an indistinct truncated base and a hilum, guttulate, smooth-walled. ***Chlamydospores*** (10.0)11.5–14.0(–16.3) μm wide (*x̄* = 12.8 μm, *n* = 50), frequent produced in aerial hyphae, mostly intercalary, solitary or in branched chains, subglobose to ellipsoidal, pigmented, light to dark brown, smooth-, thick-walled, septate, constricted near the septa. ***Endoconidia*** absent.

##### Cardinal temperatures for growth.

Minimum 10 °C, optimum in the range of 25 °C to 30 °C. No growth at 4 °C and 37 °C.

##### Strains examined.

THAILAND • Chiang Mai, Mueang District, Chang Phueak, isolated from jasmine flower (*Jasminum sambac*; *Oleaceae*, *Lamiales*), Jul 2024, P. Kodchasee, C. Senwanna, J. Kumla and N. Suwannarach, living culture = SDBR-CMU817; Phayao Province, Mueang District, isolated from holy basil flower (*Ocimum tenuiflorum*; *Lamiaceae*; *Lamiales*), Aug 2024, P. Kodchasee, C. Senwanna, J. Kumla and N. Suwannarach, living culture = SDBR-CMU855.

##### Habitat and distribution.

Flowers of *Jasminum sambac* and *Ocimum tenuiflorum* in Thailand (this study). *Aureobasidium melanogenum* is found worldwide in diverse environments and materials, including air, soil, water, and indoor and plant surfaces, and is also considered an opportunistic human pathogen (Zalar et al. 2008; Gostinčar et al. 2014; Nasr et al. 2018; da Silva et al. 2024; Černoša et al. 2025; Patel et al. 2025).

##### Notes.

The multilocus phylogenetic tree (Fig. 2) showed that strains SDBR-CMU817 and SDBR-CMU855 clustered with other strains of *A. melanogenum*. The ITS, LSU, and *tef1* sequences were identical (100% similarity) to those of *A. melanogenum* CBS 105.22 (ex-type), whereas the *rpb2* and *tub* sequences showed 98.90% and 98.00% similarity, respectively. The morphology of strains SDBR-CMU817 and SDBR-CMU855 is consistent with the species description of *A. melanogenum* provided by Zalar et al. (2008); therefore, both strains were identified as *A. melanogenum*.

#### Aureobasidium musti

Taxon classificationFungiDothidealesSaccotheciaceae

C. Onetto, S. Schmidt, M. Roach & A. Borneman ex Jumbam & Aime, Persoonia 54: 179 (2025)

BBCD4B85-C912-5DC9-9BAD-A556F10FE058

858478

Fig. 17

##### Cultural characteristics.

After 14 days of incubation at 25 °C in the dark, colonies on PDA reaching 59–67 mm diam., surface flat, spreading with filiform, entire margin, velvety, shiny, reddish white (10A2); on MEA reaching 50–57 mm diam., surface flat, round with radiating, filamentous, gradient from pale yellow (2A3), light yellow (2A5) and yellow (2A8); on OA 60–67 mm diam., surface flat, spreading with entire margin, velvety, shiny, reddish white (10A2).

**Figure 17.**
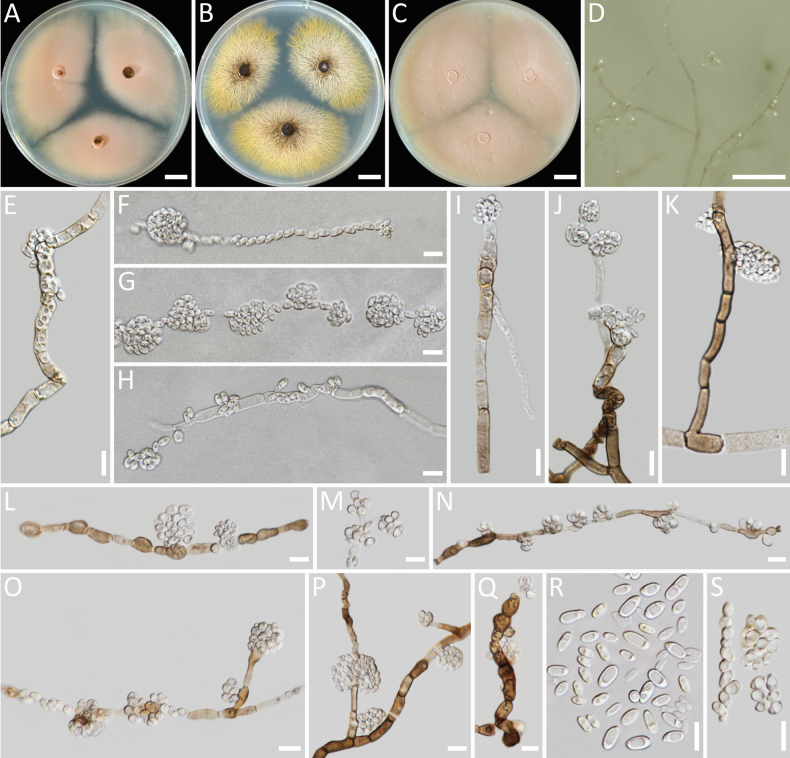
*Aureobasidium musti* (SDBR-CMU825). **A–C** colonies on PDA, MEA, and OA, respectively, after 14 d at 25 °C; **D** close-up of an immersed hyphal-tip colony on PDA after 14 d; **E, F** endoconidia; **F–N** hyphae with groups of hyaline conidia; **O, P** conidiogenous cells integrated into vegetative hyphae with hyaline conidia; **Q** melanized hyphae developing into chlamydospores; **R, S** conidia. Scale bars: 1 cm (**A–C**); 500 μm (**D**); 10 μm (**E–S**).

##### Description.

***Anamorph****in vitro* dematiaceous hyphomycetes. ***Vegetative hyphae*** (2.4–)3.0–8.0(–11.1) μm wide, hyaline to brown, becoming dark brown with age, branched, septate, smooth, thin-walled, getting darker and thicker with age, constricted at the septa. ***Conidiogenous cells*** (9.0–)9.8–14.0(–20.5) × (3.6–)4.5–6.5(–7.2) μm (*x̄* = 12.4 × 5.4 μm, *n* = 20), undifferentiated, intercalary, terminal, producing conidia percurrently from indistinct, short lateral denticles, giving rise to conidia that aggregate in a mucoid mass, or differentiated on hyphae, hyaline to dark brown. ***Conidia*** obovoid to ellipsoidal, or elongated, hyaline to light brown, aseptate, occasionally with an indistinct truncated base and a hilum, smooth-walled, guttulate, (4.5–)5.5–9.2(–10.7) × (2.3–)2.6–4.5(–4.8) μm (*x̄* = 7.6 × 3.7 μm, *n* = 150). ***Chlamydospores*** (6.2–)7.6–10.2(–11.6) (*x̄* = 8.7 μm, *n* = 25) µm wide, produced in aerial hyphae, mostly intercalary, solitary or in branched chains, subglobose, brown, smooth-, thick-walled, septate, constricted near the septa. ***Endoconidia*** (3.4–)4.3–7.2 × 2.6–3.9(–4.8) μm (*x̄* = 5.6 × 3.4 μm, *n* = 30), obovoid to ellipsoidal, hyaline, occasionally seen in intercalary hyphal cells.

##### Cardinal temperatures for growth.

Minimum 4 °C, optimum in the range of 25 °C to 30 °C, maximum 37 °C.

##### Strains examined.

THAILAND • Chiang Mai Province, Mueang District, Chang Phueak, isolated from confederate vine flower (*Antigonon leptopus*; *Polygonaceae*, *Caryophyllales*), Jul 2024, P. Kodchasee, C. Senwanna, J. Kumla and N. Suwannarach, living culture = SDBR-CMU825, Suthep, isolated from white cheesewood flower (*Alstonia scholaris*; *Apocynaceae*, *Gentianales*), Sep 2024, P. Kodchasee, C. Senwanna, J. Kumla and N. Suwannarach, living culture = SDBR-CMU864.

##### Habitat and distribution.

Flowers of *Alstonia scholaris* and *Antigonon leptopus* in Thailand (this study); endophyte from pine needles, isolated from a bird feather, the phylloplanes of *Cyrtomium falcatum*, *Lygodium japonicum*, and *Oryza sativa*, and an unidentified phylloplane in the USA (Jumbam et al. 2025); Shiraz grape juice in Australia (Jumbam et al. 2025).

##### Notes.

Phylogenetic analysis showed that strains SDBR-CMU825 and SDBR-CMU864 grouped in the same clade as *A. musti* and formed a sister clade to *A. chiangmaiense* (Fig. 2). The ITS and LSU sequences of both strains were identical (100% similarity) to those of *A. musti* NRRL 64173 (ex-type). Additionally, the morphological characteristics of SDBR-CMU825 and SDBR-CMU864 are similar to those of *A. musti*. Thus, SDBR-CMU825 and SDBR-CMU864 were identified as *A. musti* based on combined morphological and phylogenetic evidence. The differences in molecular data, morphological characteristics, and physiological traits between *A. musti* and *A. chiangmaiense* have been described above. This is the first report of *A. musti* in Thailand.

#### Aureobasidium oroxyli

Taxon classificationFungiDothidealesSaccotheciaceae

Senwanna, Kodchasee, J. Kumla & N. Suwannar. sp. nov.

6E5F4408-0342-5FDE-A8A9-C6BB67E56EBB

861967

Fig. 18

##### Etymology.

Refers to the host genus *Oroxylum*, from which this species was isolated.

**Figure 18.**
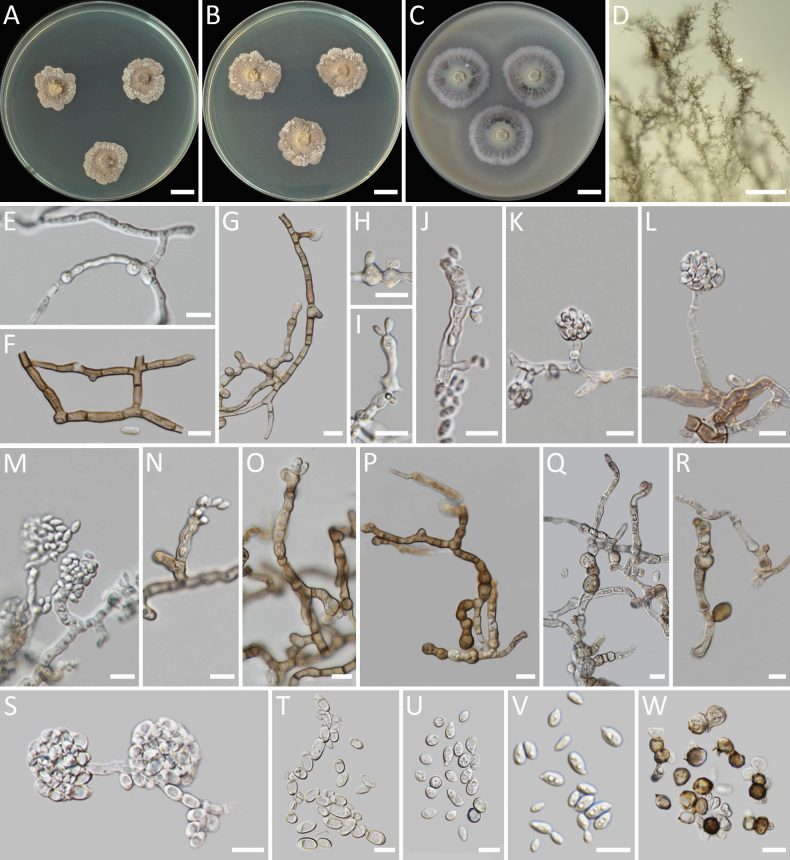
*Aureobasidium oroxyli* (SDBR-CMU827, ex-type). **A–C** colonies on PDA, MEA, and OA, respectively, after 14 d at 25 °C; **D** close-up of an immersed hyphal-tip colony on PDA after 14 d; **E, F** hyphal anastomoses; **G–J** hyphae with lateral pegs; **K, L** conidiogenous cells integrated into vegetative hyphae with hyaline conidia; **M–O***Hormonema*-like conidiogenous cells with hyaline conidia; **P–R** melanized hyphae developing into chlamydospores; **S** hyphae with a group of conidia; **P–V** conidia, some exhibiting yeast-like budding; **W** chlamydospores. Scale bars: 1 cm (**A–C**); 500 μm (**D**); 10 μm (**E–W**).

##### Type.

THAILAND • Chiang Mai Province, Phrao District, Nam Phrae, isolated from Indian trumpet flower (*Oroxylum indicum*; *Bignoniaceae*, *Lamiales*), Jul 2024, P. Kodchasee, C. Senwanna, J. Kumla and N. Suwannarach, ***holotype*** = CMUB40133 (preserved in metabolically inactive state), living culture (ex-type) = SDBR-CMU827 = GMBCC2499 = TBRC 21786.

##### Cultural characteristics.

After 14 days of incubation at 25 °C in the dark, colonies on PDA reaching 18–21 mm diam., surface round with raised, wrinkled margin, dry, reddish gray (10B2), with light gray (10C1) at the margin; on MEA reaching 20–25 mm diam., surface filamentous, slimy, round with raised, wrinkled, dry margin, grayish yellow (4B6) at center, with light gray (4C1) to yellowish gray (4B2) at the margin; on OA 25–30 mm diam., surface flat, spreading with irregular margin, wrinkled, yellowish white (2A2) at center, gradient from violet gray (17D2) to violet white (17A2), surrounded by light gray (17B1) clear zones on an agar medium.

##### Description.

***Anamorph****in vitro* dematiaceous hyphomycetes. ***Vegetative hyphae*** (2.0–)3.2–6.5(–8.0) μm wide, hyaline to brown, branched, septate, smooth, thin-walled, getting darker and thicker with age, constricted at the septa, anastomosis observed. ***Conidiogenous cells*** 9.0–18.0(–22.4) × 2.9–5.2(–6.4) μm (*x̄* = 13.8 × 4.2 μm, *n* = 25), undifferentiated, intercalary, rarely terminal, producing conidia percurrently from short lateral denticles, giving rise to conidia that aggregate in a mucoid mass, or differentiated on hyphae, hyaline to brown. ***Conidia (***4.0–)5.2–8.7(–11.3) × (2.0–)2.6–5.0(–7.0) μm (*x̄* = 6.8 × 3.6 μm, *n* = 200), obovoid to ellipsoidal, subglobose, hyaline, aseptate, occasionally with an indistinct truncated base and a hilum, smooth-walled, guttulate, budding occasionally observed. ***Chlamydospores*** 6.9–14.0(–17.5) μm (*x̄* = 10.6 μm, *n* = 25) µm wide, produced in aerial hyphae, mostly intercalary, solitary or in branched chains, subglobose to ellipsoidal, pigmented, light to dark brown, smooth-, thick-walled, septate, constricted near the septa. ***Endoconidia*** absent.

Fermentation of glucose is negative. *D*-glucose, galactose, sorbose, ribose, xylose, *L*-arabinose, *D*-arabinose, *L*-rhamnose, sucrose, maltose, *α*-*α*-trehalose, methyl-*α*-*D*-glucoside (weak), cellobiose, salicin, melibiose (weak), lactose, raffinose, melizitose, glycerol, erythritol, ribitol (weak), glucitol (weak), mannitol, *myo*-inositol, *D*-glucono-1,5-lactone, *D*-gluconate, *D*-glucuronate, *D*-galacturonic acid (weak), succinate, citrate, ethanol, and xylitol are assimilated, but *N*-acetyl glucosamine, inulin, soluble starch, galactitol, *DL*-lactate, and methanol are not assimilated. Ammonium sulfate, ethylamine hydrochloride, *L*-lysine, cadaverine, and creatine are assimilated as sole nitrogen sources. Potassium nitrate and sodium nitrite are not assimilated. After 3 weeks, light pink pigmentation was observed in *D*-glucose. Brown sediment formed in *D*-xylose, *L*-arabinose, melibiose, and raffinose, while light brown sediment produced in cellobiose. Dark brown sediment presents in *D*-mannitol and *D*-gluconate, whereas dark sediment observed in *D*-ribose.

##### Cardinal temperatures for growth.

Minimum 10 °C, optimum in the range of 25 °C to 30 °C, maximum 37 °C. No growth at 4 °C.

##### Additional strains examined.

THAILAND • Chiang Mai Province, Mueang District, Chang Phueak, isolated from Indian trumpet flower (*Oroxylum indicum*; *Bignoniaceae*, *Lamiales*), Jul 2024, P. Kodchasee, C. Senwanna, J. Kumla and N. Suwannarach, living culture = SDBR-CMU822.

##### Habitat and distribution.

Flowers of *Oroxylum indicum*. Known only from northern Thailand.

##### Notes.

Multilocus phylogenetic analyses revealed that *A. oroxyli* strains SDBR-CMU822 and SDBR-CMU827 (ex-type) formed a distinct monophyletic lineage with 100% MLBS and 1.00 BIPP support and were sister to *A. tremulum* (Fig. 2). The ITS and LSU sequence data of *A. oroxyli* showed similarities of 96.92% and 98.80%, respectively, to those of *A. tremulum* UN 1 (ex-type). However, the *rpb2*, *tef1*, and *tub* sequences could not be compared because sequences from the ex-type strain of *A. tremulum* were unavailable. Comparisons of ITS, LSU, *rpb2*, *tef1*, and *tub* sequences showed that *A. oroxyli* shared 99.56%, 96.99–97.32%, 91.39%, 79.86–80.65%, and 90.57% similarity, respectively, with six *A. tremulum* strains obtained in this study. Because of the limited information available for *A. tremulum*, which was originally described only from yeast cells, detailed morphological comparisons could not be made (Crous et al. 2019). Furthermore, *A. oroxyli* and the *A. tremulum* strains obtained in this study can be morphologically distinguished by their colony coloration on MEA, with *A. oroxyli* forming grayish-yellow colonies with light-gray to yellowish-gray margins, whereas *A. tremulum* formed brownish-gray colonies with purplish-gray margins. Growth at 37 °C was observed in *A. oroxyli*, whereas *A. tremulum* did not grow at this temperature (Crous et al. 2019; this study). Moreover, the conidia of *A. oroxyli* (4.0–11.3 µm) are generally shorter than those of *A. tremulum* (4.6–17.3 µm). *Aureobasidium oroxyli* can be distinguished from *A. tremulum* by its ability to assimilate lactose and glycerol and to produce pigmentation when grown on *D*-glucose (Crous et al. 2019). Thus, *A. oroxyli* is introduced as a novel species based on morphological and phylogenetic evidence.

#### Aureobasidium phayaoense

Taxon classificationFungiDothidealesSaccotheciaceae

Senwanna, Kodchasee, J. Kumla & N. Suwannar. sp. nov.

FA33F582-F70D-52FB-BA1A-1C13F820CB89

861968

Fig. 19

##### Etymology.

Refers to Phayao Province, where the flowers containing the new fungus were collected.

**Figure 19.**
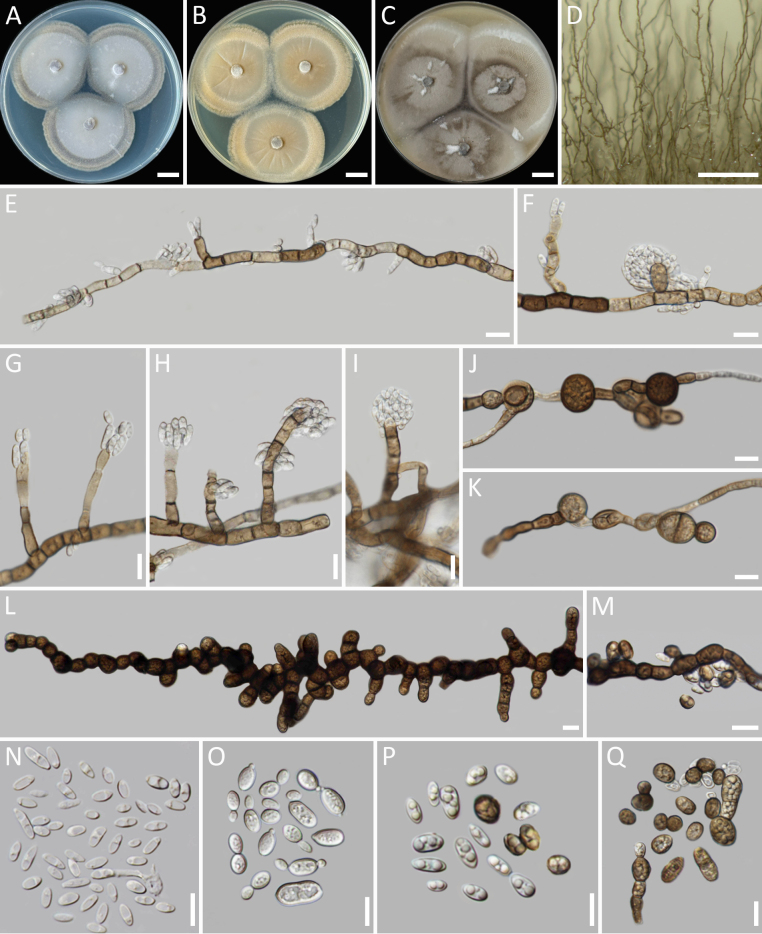
*Aureobasidium phayaoense* (SDBR-CMU852, ex-type). **A–C** colonies on PDA, MEA, and OA, respectively, after 14 d at 25 °C; **D** close-up of an immersed hyphal-tip colony on PDA after 14 d; **E–I** conidiogenous cells integrated into vegetative hyphae with hyaline conidia; **J, K** chlamydospores; **L** melanized hypha; **M** melanized hyphae with hyaline and melanized conidia; **N, O** conidia, some exhibiting yeast-like budding; **P, Q** hyaline and melanized conidia produced from melanized hyphae. Scale bars: 1 cm (**A–C**); 500 μm (**D**); 10 μm (**E–Q**).

##### Type.

THAILAND • Phayao Province, Mueang District, isolated from Egyptian star cluster flower (*Pentas lanceolata*; *Rubiaceae*, *Gentianales*), Aug 2024, P. Kodchasee, C. Senwanna, J. Kumla and N. Suwannarach, ***holotype*** = CMUB40126 (preserved in metabolically inactive state), living culture (ex-type) = SDBR-CMU852 = GMBCC2508 = TBRC 21787.

##### Cultural characteristics.

After 14 days of incubation at 25 °C in the dark, colonies on PDA reaching 39–45 mm diam., surface flat, spreading with entire margin, velvety, concentric, gradient from gray (4B1), brownish gray (4E2) to grayish brown (7E3), with yellowish white (2A2) at the margin; on MEA reaching 40–48 mm diam., surface flat, spreading with entire margin, wrinkled, velvety, concentric, gradient from dull yellow (3B3) to grayish yellow (4B6), with yellowish white (2A2) at the margin; on OA 55–65 mm diam., surface flat, spreading with entire margin, wrinkled, brownish orange (6C8), grayish brown (6E3) with light gray (6B1) to brownish gray (4E2) at the margin.

##### Description.

***Anamorph****in vitro* dematiaceous hyphomycetes. ***Vegetative hyphae*** (2.0–)3.0–9.0(–12.0) μm wide, hyaline, light to brown with age, branched, septate, smooth, thin-walled, becoming dark melanized, getting darker and thicker with age, constricted at the septa, minute echinulate. ***Conidiogenous cells*** (7.5–)8.7–13.8(–17.4) × (3.6–)4.5–6.5(–7.5) μm (*x̄* = 10.9 × 5.5 μm, *n* = 25) µm, undifferentiated, intercalary, terminal, producing conidia percurrently from indistinct, short lateral denticles, giving rise to conidia that aggregate in a mucoid mass, or differentiated on hyphae, occasionally elongated, solitary, light to dark brown. ***Conidia*** (4.0–)5.5–9.6(–11.8) × (1.9–)2.8–6.0(–7.4) μm (*x̄* = 7.6 × 4.3 μm, *n* = 170), oval, obovoid to ellipsoidal or elongated, or subglobose, hyaline, becoming light brown to brown with age, aseptate, occasionally with an indistinct truncated base and a hilum, guttulate, smooth-walled, budding occasionally observed. ***Chlamydospores*** (9.0–)10.4–14.0(–17.5) µm wide (*x̄* = 12.6 μm, *n* = 30), frequent produced in aerial hyphae, mostly intercalary, solitary, subglobose to ellipsoidal, pigmented, light to dark brown, septate, smooth-, thick-walled. ***Endoconidia*** absent.

Fermentation of glucose is negative. *D*-glucose, galactose, sorbose, *N*-acetyl glucosamine (weak), ribose, xylose, *L*-arabinose, *D*-arabinose, *L*-rhamnose, sucrose, maltose, *α-α-*trehalose, methyl-*α*-*D*-glucoside, cellobiose, salicin, melibiose (weak), lactose, raffinose, melizitose, glycerol, erythritol, ribitol, glucitol, mannitol, galactitol, *myo*-inositol, *D*-glucono-1,5-lactone, *D*-gluconate, *D*-glucuronate, *D*-galacturonic acid (weak), citrate, ethanol, and xylitol are assimilated, but inulin, soluble starch, *DL*-lactate, succinate, and methanol are not assimilated. Ammonium sulfate, ethylamine hydrochloride, *L*-lysine, cadaverine, and creatine are assimilated as sole nitrogen sources. Potassium nitrate and sodium nitrite are not assimilated. After 3 weeks, dark brown sediment formed in *N*-acetyl glucosamine, *D*-xylose, *L*-arabinose, *D*-arabinose, *L*-rhamnose, sucrose, maltose, methyl-*α*-*D*-glucoside, cellobiose, salicin, melibiose, lactose, raffinose, melizitose, glycerol, erythritol, ribitol, D-glucitol, galactitol, *D*-glucono-1,5-lactone, *D*-gluconate, *D*-glucuronate, *D*-galacturonic acid, and citrate, while light brown sediment formed in *α*-*α*-trehalose. Black sediment formed in *D*-glucose, *D*-galactose, *L*-sorbose, *D*-ribose, *D*-mannitol, *myo*-inositol, and xylitol, while white sediment observed in ethanol.

##### Cardinal temperatures for growth.

Minimum 10 °C, optimum in the range of 25 °C to 30 °C, maximum 37 °C. No growth at 4 °C.

##### Additional strains examined.

THAILAND • Phayao Province, Mueang District, isolated from bush clock vine flower (*Thunbergia erecta*; *Acanthaceae*, *Lamiales*), Aug 2024, P. Kodchasee, C. Senwanna, J. Kumla and N. Suwannarach, living culture = SDBR-CMU811 and SDBR-CMU857.

##### Habitat and distribution.

Flowers of *Pentas lanceolata* and *Thunbergia erecta*. Known only from northern Thailand.

##### Notes.

Phylogenetic analyses showed that *A. phayaoense* SDBR-CMU852 (ex-type) and SDBR-CMU857 formed a distinct lineage sister to *A. pollinicola*, with 80% MLBS and 0.90 BIPP support (Fig. 2). Comparisons of ITS, LSU, *rpb2*, *tef1*, and *tub* nucleotide sequences showed that *A. phayaoense* differed from *A. pollinicola* by 1.01% (6/592 bp, including gaps), 0.33% (3/901 bp, including gaps), 4.85% (46/948 bp, including gaps), 7.83% (21/268 bp, including gaps), and 2.93% (12/409 bp, including gaps), respectively. Although comparisons of the ITS, LSU, and *tub* loci indicated that *A. phayaoense* was not markedly different from *A. pollinicola*, the *rpb2* and *tef1* sequences revealed clear differences. Morphologically, the colony colors of *A. phayaoense* and *A. pollinicola* differed across culture media. On PDA, *A. phayaoense* produced gray to grayish-brown colonies with a yellowish-white margin, whereas *A. pollinicola* formed light- to dark-yellowish colonies with a yellowish-white margin. On MEA, colonies of *A. phayaoense* were dull yellow to grayish yellow with a yellowish-white margin, compared with the grayish-yellow colonies with a light-gray margin of *A. pollinicola*. On OA, *A. phayaoense* produced brownish-orange to grayish-brown colonies with a light-gray to brownish-gray margin, whereas *A. pollinicola* formed yellowish-white colonies with a light-gray margin. The micromorphological features of *A. phayaoense* and *A. pollinicola* are similar in size; however, *A. phayaoense* can be distinguished from *A. pollinicola* by the absence of endoconidium production. Moreover, PHI analyses revealed no evidence of significant recombination between *A. phayaoense* and *A. pollinicola* (Fig. 3). *Aureobasidium phayaoense* can be distinguished from *A. pollinicola* by its inability to assimilate *DL*-lactate and succinate (Table 1). Hence, *A. phayaoense* is introduced as a new species based on combined morphological and phylogenetic evidence.

#### Aureobasidium plumeriae

Taxon classificationFungiDothidealesSaccotheciaceae

Senwanna, Kodchasee, J. Kumla & N. Suwannar. sp. nov.

2D7E48B6-1BE7-5E56-9487-2B3AE134DD96

861969

Fig. 20

##### Etymology.

Refers to the host genus *Plumeria*, from which this species was isolated.

**Figure 20.**
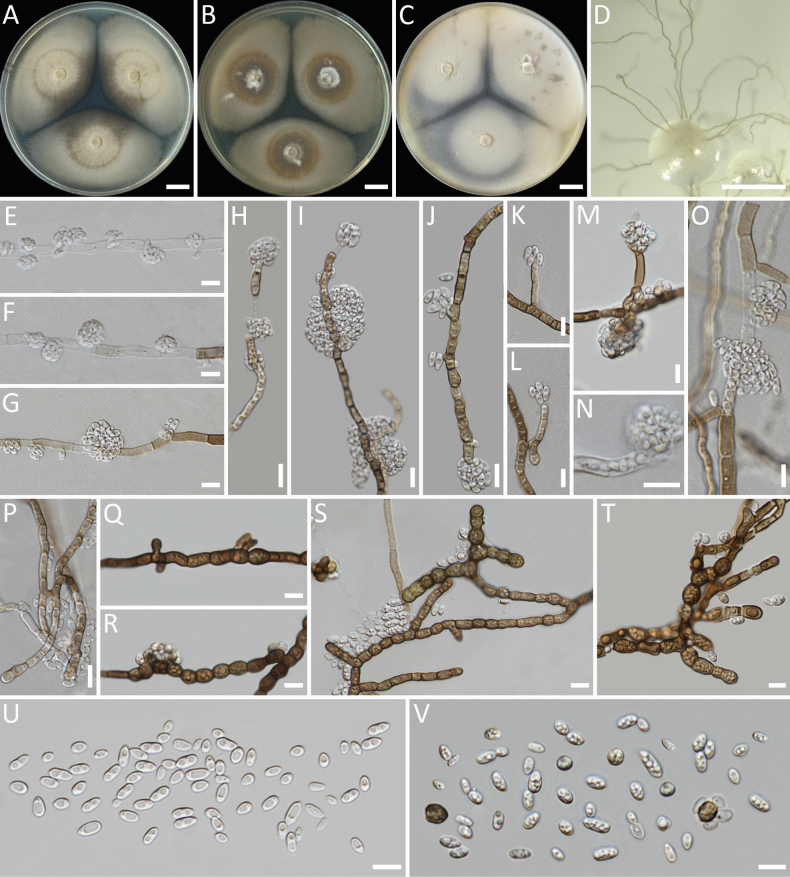
*Aureobasidium plumeriae* (SDBR-CMU836, ex-type). **A–C** colonies on PDA, MEA, and OA, respectively, after 14 d at 25 °C; **D** close-up of an immersed hyphal-tip colony on PDA after 14 d; **E–I** hyphae with groups of hyaline conidia; **J** hyphae with lateral pegs; **K–M** conidiogenous cells integrated into vegetative hyphae with hyaline conidia; **N, O** endoconidia; **P** hyphal anastomosis; **Q, S, T** development of melanized hyphae; **R** melanized hyphae with conidia; **U** conidia; **V** conidia produced from a melanized hypha. Scale bars: 1 cm (**A–C**); 500 μm (**D**); 10 μm (**E–V**).

##### Type.

THAILAND • Chiang Mai Province, Mueang District, Chang Phueak, isolated from frangipani flower (*Plumeria obtusa*; *Apocynaceae*, *Gentianales*), Jul 2024, P. Kodchasee, C. Senwanna, J. Kumla and N. Suwannarach, ***holotype*** = CMUB40128 (preserved in metabolically inactive state), living culture (ex-type) = SDBR-CMU836 = GMBCC2502 = TBRC 21788.

##### Cultural characteristics.

After 14 days of incubation at 25 °C in the dark, colonies on PDA reaching 58–64 mm diam., surface flat, spreading with entire margin, velvety, concentric, yellowish white (2A2), olive gray (1D2), with white to olive gray (1D2) at the margin; on MEA reaching 55–60 mm diam., surface flat, spreading with filiform margin, velvety, yellowish white (2A2) or pale yellow (2A3) at center, gradient from dark to light olive (2F6), with yellowish gray (4B2) to dull yellow (3B3) at the margin; on OA 65–70 mm diam., surface flat, spreading with entire margin, velvety, yellowish white (2A2), with light gray (2C1) at the margin.

##### Description.

***Anamorph****in vitro* dematiaceous hyphomycetes. ***Vegetative hyphae*** (2.2–)3.3–8.0(–10) μm wide, hyaline, becoming light brown to brown melanized with age, branched, septate, smooth, thin-walled, getting thicker, constricted at the septa with age, anastomosis observed. ***Conidiogenous cells*** (4.8–)7.0–14.0(–18) × 3.0–5.0 μm (*x̄* = 11.9 × 3.8 μm, *n* = 25), undifferentiated, intercalary, terminal, producing conidia percurrently from short lateral denticles, giving rise to conidia that aggregate in a mucoid mass, or differentiated on hyphae, hyaline to dark brown. ***Conidia*** (4.2–)5.2–8.7(–11.7) × 2–4.6(–6.0) μm (*x̄* = 7.0 × 3.9 μm, *n* = 200), oval, ellipsoidal to obovoid, or globose to subglobose, hyaline, becoming light brown to brown with age, aseptate, occasionally with an indistinct truncated base and a hilum, guttulate, smooth-walled. ***Chlamydospores*** (6.5–)7.8–9.0(–10) (*x̄* = 8.5 μm, *n* = 25) µm wide, produced in aerial hyphae, intercalary, solitary or in branched chains, subglobose, dark brown, smooth-, thick-walled, septate, constricted near the septa. ***Endoconidia*** 3.2–5.6(–6.3) × 1.8–2.8(–3.5) μm (*x̄* = 4.4 × 2.6 μm, *n* = 25), obovoid to ellipsoidal, hyaline, occasionally seen in intercalary hyphal cells.

Fermentation of glucose is negative. *D*-glucose, galactose (weak), sorbose (weak), *N*-acetyl glucosamine, ribose, xylose, *L*-arabinose, *D*-arabinose, *L*-rhamnose, sucrose, maltose, *α*-*α*-trehalose, methyl-*α*-*D*-glucoside, cellobiose, salicin, melibiose (weak), lactose (weak), raffinose, melizitose (weak), glycerol (weak), erythritol (weak), ribitol, glucitol, mannitol, galactitol, *myo*-inositol (weak), *D*-glucono-1,5-lactone, *D*-gluconate, *D*-glucuronate (weak), *D*-galacturonic acid (weak), *DL*-lactate, succinate (weak), citrate (weak), ethanol, and xylitol are assimilated, but inulin, soluble starch, and methanol are not assimilated. Ammonium sulfate, ethylamine hydrochloride, *L*-lysine, cadaverine, and creatine are assimilated as sole nitrogen sources. Potassium nitrate and sodium nitrite are not assimilated. After 3 weeks, black sediment formed in *D*-glucose, *D*-galactose, *L*-sorbose, *N*-acetyl glucosamine, *D*-xylose, *L*-arabinose, *L*-rhamnose, sucrose, maltose, *α*-*α*-trehalose, methyl-*α*-*D*-glucoside, cellobiose, salicin, melibiose, lactose, raffinose, melizitose, glycerol, erythritol, ribitol, *D*-glucitol, *D*-mannitol, galactitol, *myo*-inositol, *D*-glucono-1,5-lactone, *D*-gluconate, *D*-glucuronate, *D*-galacturonic acid, *DL*-lactate, succinate, and citrate, while white sediment observed in *D*-ribose, *D*-arabinose, ethanol, and xylitol.

##### Cardinal temperatures for growth.

Minimum 10 °C, optimum in the range of 25 °C to 30 °C. No growth at 4 °C and 37 °C.

##### Additional strains examined.

THAILAND • Chiang Mai Province, Mueang District, Chang Phueak, isolated from frangipani flower (*Plumeria obtusa*; *Apocynaceae*, *Gentianales*), Jul 2024, P. Kodchasee, C. Senwanna, J. Kumla and N. Suwannarach, living culture = SDBR-CMU826.

##### Habitat and distribution.

Flowers of *Plumeria obtusa*. Known only from northern Thailand.

##### Notes.

The multilocus phylogenetic tree (Fig. 2) revealed that *A. plumeriae* SDBR-CMU826 and SDBR-CMU836 (ex-type) formed a monophyletic lineage with strong support (100% MLBS and 0.98 BIPP). *Aureobasidium plumeriae* formed a sister clade to *A. motuoense*, *A. phayaoense*, *A. pollinicola*, and *A. siamense*. Sequence comparison showed that the ITS sequence of *A. plumeriae* showed 98.77%, 99.66%, 99.83%, and 100% similarity to those of *A. motuoense*, *A. siamense*, *A. pollinicola*, and *A. phayaoense*, respectively, whereas the LSU sequences showed 100% similarity among all related species. In contrast, comparisons of the *rpb2*, *tef1*, and *tub* sequences revealed lower sequence similarity between *A. plumeriae* and its closest relatives, with 96.01%, 92.83%, and 97.24% similarity to *A. phayaoense*; 96.33%, 91.99%, and 97.24% similarity to *A. pollinicola*; and 96.24%, 92.44%, and 97.24% similarity to *A. siamense*, respectively. However, differences in the *rpb2*, *tef1*, and *tub* sequence data could not be compared because sequences of *A. motuoense* were unavailable. Morphologically, the colony characteristics of *A. plumeriae* differed from those of most phylogenetically related sister taxa. In terms of growth temperature, *A. plumeriae* did not grow at 37 °C, whereas *A. phayaoense* and *A. pollinicola* grew at this temperature. *Aureobasidium plumeriae* differs from *A. phayaoense* and *A. siamense* by the presence of endoconidia. In addition, *A. plumeriae* differs from *A. motuoense*, *A. pollinicola*, and *A. siamense* in having smaller conidia [(4.2–)5.2–8.7(–11.7) × 2.0–4.6(–6.0) μm vs. (6.5–)10.0–17.0 × 3.9–6.5(–7.4) μm vs. (3.2–)6.0–9.4(–14.5) × (2.1–)3.3–5.5(–6.6) μm vs. (5.1–)7.4–11.0(–16.2) × (2.1–)3.6–5.9(–8.2) μm] (Wu et al. 2023; this study). Furthermore, the PHI test indicated no significant recombination among these taxa (Fig. 3), supporting the recognition of *A. motuoense*, *A. phayaoense*, *A. plumeriae*, *A. pollinicola*, and *A. siamense* as distinct species. Based on physiological tests, *A. plumeriae* differs from *A. phayaoense* in its ability to assimilate *DL*-lactate and succinate (Table 1). In contrast, *A. siamense* differs from *A. plumeriae* by producing pigment when grown on *D*-glucono-1,5-lactone. Moreover, *A. pollinicola* can be differentiated from *A. plumeriae* by differences in sediment formation characteristics (Table 1). Thus, *A. plumeriae* is introduced as a new species based on phylogenetic analyses, morphological and phenotypic characteristics, and growth temperature data.

#### Aureobasidium pollinicola

Taxon classificationFungiDothidealesSaccotheciaceae

Senwanna, Kodchasee, J. Kumla & N. Suwannar. sp. nov.

A6F08C3C-BCA2-50C1-8729-572267504B38

861970

Fig. 21

##### Etymology.

Refers to its association with pollen, from which this species was isolated.

**Figure 21.**
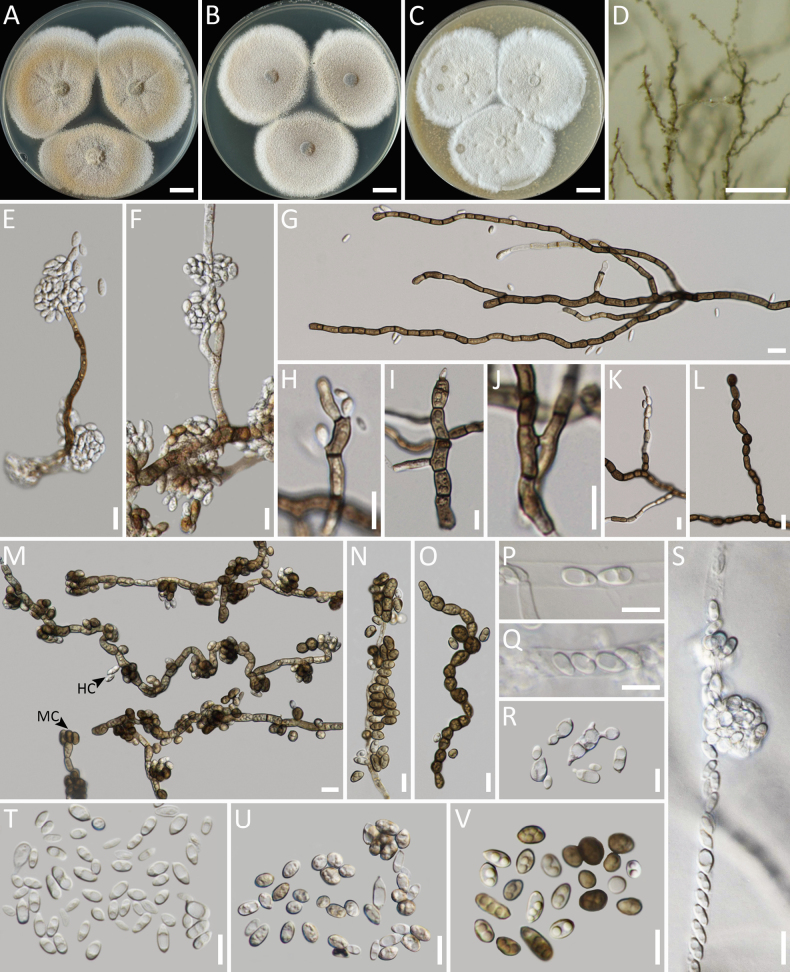
*Aureobasidium pollinicola* (SDBR-CMU861, ex-type). **A–C** colonies on PDA, MEA, and OA, respectively, after 14 d at 25 °C; **D** close-up of an immersed hyphal-tip colony on PDA after 14 d; **E–G** hyphae with groups of hyaline conidia; **H, I** hyphae with multiple lateral pegs and conidia; **J** hyphal anastomosis; **K, L** hyphae developing into melanized hyphae; **M, N** melanized hyphae with groups of hyaline conidia (HC) and melanized conidia (MC); **O** melanized hyphae developing into chlamydospores; **P, Q, S** endoconidia; **T** conidia; **U, V** hyaline and melanized conidia produced from melanized hyphae. Scale bars: 1 cm (**A–C**); 500 μm (**D**); 10 μm (**E–V**).

##### Type.

THAILAND • Chiang Mai Province, Mueang District, Chang Phueak, isolated from marguerite daisy flower (*Argyranthemum frutescens*; *Asteraceae*, *Asterales*), Jul 2024, P. Kodchasee, C. Senwanna, J. Kumla and N. Suwannarach, ***holotype*** = CMUB40134 (preserved in metabolically inactive state), living culture (ex-type) = SDBR-CMU861 = GMBCC2510 = TBRC 21789.

##### Cultural characteristics.

After 14 days of incubation at 25 °C in the dark, colonies on PDA reaching 50–55 mm diam., surface flat, spreading with entire margin, powdery, wrinkled, gradient from light to dark yellowish gray (4B2), with yellowish white (2A2) at the margin; on MEA reaching 40–46 mm diam., surface flat, powdery, spreading with entire margin, grayish yellow (4B4), with light gray (2B2) at the margin; on OA 42–47 mm diam., surface flat, powdery, wrinkled, spreading with entire margin, yellowish white (2A2), with light gray (2B2) at the margin.

##### Description.

***Anamorph****in vitro* dematiaceous hyphomycetes. ***Vegetative hyphae*** (2.8–)3.5–8.0(–9.5) μm wide, hyaline to light brown, becoming dark brown melanized, branched, septate, smooth-, thin-walled, getting thicker, constricted at the septa with age. ***Conidiogenous cells*** (6.0–)10.0–16.0(–24.6) × (3.8–)4.0–5.7 μm (*x̄* = 13.0 × 4.6 μm, *n* = 25), undifferentiated, intercalary, terminal, producing conidia percurrently from indistinct, short lateral denticles, giving rise to conidia that aggregate in a mucoid mass, or differentiated on hyphae, hyaline to dark brown. ***Conidia*** fusoid, obovoid to ellipsoidal, or oval, or globose to subglobose, hyaline, becoming light brown to brown melanized, aseptate, occasionally with an indistinct truncated base and a hilum, guttulate, smooth-walled, (3.2–)6.0–9.4(–14.5) × (2.1–)3.3–5.5(–6.6) μm (*x̄* = 7.7 × 4.5 μm, *n* = 200), budding occasionally observed. ***Chlamydospores*** (6.8–)7.5–9.1(–10.2) μm wide (*x̄* = 8.3 μm, *n* = 25), produced in aerial hyphae, mostly intercalary, solitary or in branched chains, globose to subglobose, light to dark brown, smooth-, thick-walled, septate, 1–2 cells, constricted near the septa. ***Endoconidia*** (2.9–)5.5–7.5(–8.2) × (2.0–)2.3–3.8(–4.5) μm (*x̄* = 6.5 × 3.2 μm, *n* = 35), obovoid to ellipsoidal, hyaline, occasionally seen in intercalary hyphal cells.

Fermentation of glucose is negative. *D*-glucose, galactose, sorbose, *N*-acetyl glucosamine, ribose, xylose, *L*-arabinose (weak), *D*-arabinose, *L*-rhamnose (weak), sucrose (weak), maltose, *α*-*α*-trehalose (weak), methyl-*α*-*D*-glucoside (weak), cellobiose (weak), salicin (weak), melibiose (weak), lactose, raffinose (weak), melizitose, glycerol, erythritol (weak), ribitol, glucitol, mannitol (weak), galactitol (weak), *myo*-inositol (weak), *D*-glucono-1,5-lactone (weak), *D*-gluconate, *D*-glucuronate (weak), *D*-galacturonic acid, *DL*-lactate (weak), succinate, citrate, ethanol (weak), and xylitol (weak) are assimilated, but inulin, soluble starch, and methanol are not assimilated. Ammonium sulfate, ethylamine hydrochloride, *L*-lysine, cadaverine, and creatine are assimilated as sole nitrogen sources. Potassium nitrate and sodium nitrite are not assimilated. After 3 weeks, light brown sediment formed in *D*-xylose, erythritol, *D*-mannitol, galactitol, and *myo*-inositol. Black sediment formed in *D*-glucose, *D*-galactose, *L*-sorbose, *N*-acetyl glucosamine, *D*-ribose, *L*-arabinose, *D-*arabinose, *L*-rhamnose, sucrose, maltose, *α*-*α*-trehalose, methyl-*α*-*D*-glucoside, cellobiose, salicin, melibiose, lactose, raffinose, melizitose, glycerol, ribitol, *D*-glucitol, *D*-glucono-1,5-lactone, *D*-gluconate, *D*-glucuronate, *D*-galacturonic acid, *DL*-lactate, succinate, citrate, ethanol, and xylitol.

##### Cardinal temperatures for growth.

Minimum 10 °C, optimum in the range of 25 °C to 30 °C, maximum 37 °C. No growth at 4 °C.

##### Additional strains examined.

THAILAND • Phayao Province, Mueang District, isolated from frangipani flower (*Plumeria obtusa*; *Apocynaceae*, *Gentianales*), Aug 2024, P. Kodchasee, C. Senwanna, J. Kumla and N. Suwannarach, living culture = SDBR-CMU810; isolated from vinca flower (*Catharanthus roseus*; *Apocynaceae*, *Gentianales*), Aug 2024, P. Kodchasee, C. Senwanna, J. Kumla and N. Suwannarach, living culture = SDBR-CMU813 and SDBR-CMU843.

##### Habitat and distribution.

Flowers of *Argyranthemum frutescens*, *Catharanthus roseus*, and *Plumeria obtusa*. Known only from northern Thailand.

##### Notes.

Phylogenetic analyses (Fig. 2) showed that *A. pollinicola* SDBR-CMU810, SDBR-CMU813, SDBR-CMU843, and SDBR-CMU861 (ex-type) formed a distinct monophyletic lineage with 100% MLBS and 0.97 BIPP support that was sister to *A. phayaoense*. However, molecular sequence data, morphological and physiological characteristics, and PHI analyses clearly distinguished them as distinct species, as mentioned above. Therefore, *A. pollinicola* is described herein as a new species.

#### Aureobasidium saisamorniae

Taxon classificationFungiDothidealesSaccotheciaceae

Senwanna, Kodchasee, J. Kumla & N. Suwannar. sp. nov.

806CADC2-75D7-5469-91B0-61E6A6FBCA9F

861972

Fig. 22

##### Etymology.

Named in honor of Thai mycologist Saisamorn Lumyong in celebration of her 75th birthday.

**Figure 22.**
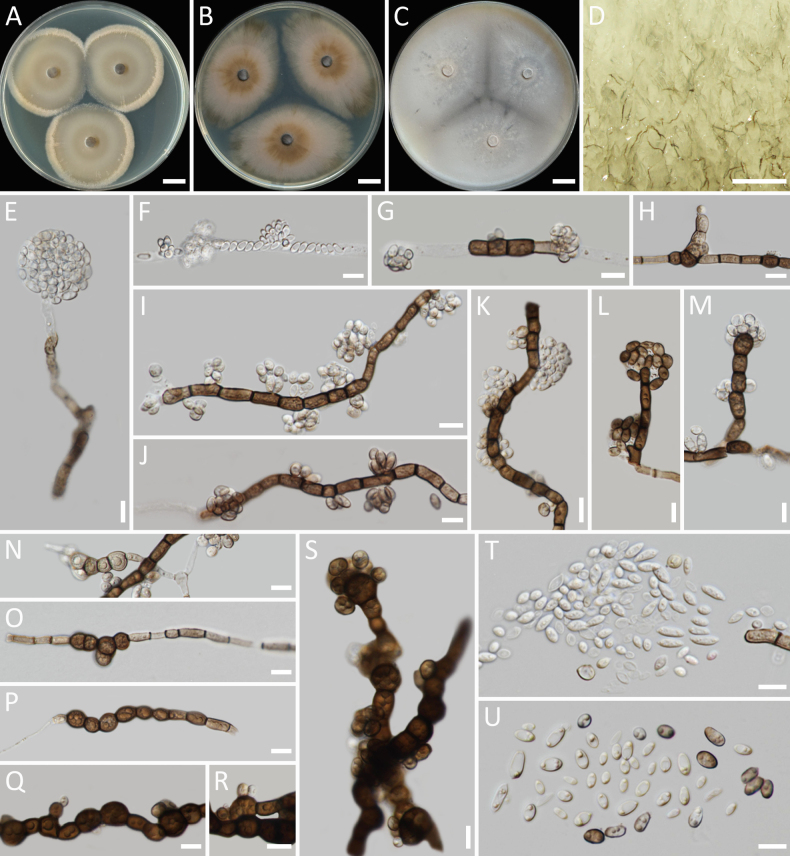
*Aureobasidium saisamorniae* (SDBR-CMU848, ex-type). **A–C** colonies on PDA, MEA, and OA, respectively, after 14 d at 25 °C; **D** close-up of an immersed hyphal-tip colony on PDA after 14 d; **E** hyphae with a group of conidia; **F** endoconidia; **G–J** conidiogenous apparatus; **H, L** conidiogenous cells integrated into vegetative hyphae with groups of conidia; **M, Q–S** melanized hyphae with groups of hyaline and melanized conidia; **N–P** development of chlamydospores; **T** conidia; **U** hyaline and melanized conidia from melanized hyphae. Scale bars: 1 cm (**A–C**); 500 μm (**D**); 10 μm (**E–U**).

##### Type.

THAILAND • Phayao Province, Mueang District, isolated from butter daisy flower (*Melampodium divaricatum*; *Asteraceae*, *Asterales*), Jul 2024, P. Kodchasee, C. Senwanna, J. Kumla and N. Suwannarach, ***holotype*** = CMUB40131 (preserved in metabolically inactive state), living culture (ex-type) = SDBR-CMU848 = GMBCC2505 = TBRC 21790.

##### Cultural characteristics.

After 14 days of incubation at 25 °C in the dark, colonies on PDA reaching 39–41 mm diam., surface flat, spreading, round with slight raised margin, velvety, gradient from light to dark yellowish gray (4B2), with pale yellow (2A3) at the margin; on MEA reaching 45–59 mm diam., surface flat, spreading with filiform, entire margin, yellowish brown (5E8) at center, pale orange (6A3), with grayish yellow (4B4) at the margin; on OA 60–65 mm diam., surface flat, spreading with entire margin, yellowish white (2A2), with light gray (2B1) at the margin.

##### Description.

***Anamorph****in vitro* dematiaceous hyphomycetes. ***Vegetative hyphae*** (2.5–)3.9–6.5(–10) μm wide, hyaline to dark brown, branched, septate, smooth, thin-walled, getting darker and thicker with age, constricted at the septa. ***Conidiogenous cells*** 8.3–10.6(–16.4) × 4.0–5.7(–9.6) μm (*x̄* = 10.4 × 5.7 μm, *n* = 25), undifferentiated, intercalary, terminal, producing conidia percurrently from indistinct, short lateral denticles, giving rise to conidia that aggregate in a mucoid mass, or differentiated on hyphae, hyaline to dark brown. ***Conidia*** (3.6–)5.4–8(–10.7) × 2.2–4.5(–5.7) μm (*x̄* = 6.5 × 3.5 μm, *n* = 200) µm, fusoid, obovoid to ellipsoidal, or subglobose, hyaline, becoming light brown to brown with age, aseptate, occasionally with an indistinct truncated base and a hilum, guttulate, smooth-walled. ***Chlamydospores*** (6.5–)7.2–13.5(–16.0) μm wide (*x̄* = 9.8 μm, *n* = 35), frequent produced in aerial hyphae, mostly intercalary, solitary, subglobose to ellipsoidal, pigmented, light to dark brown, smooth-, thick-walled, septate. ***Endoconidia*** (4.5–)5.2–7.0(–7.8) × (2.0–)2.5–3.7(–4.5) μm (*x̄* = 6.1 × 3.3 μm, *n* = 25), obovoid to ellipsoidal, hyaline, occasionally seen in intercalary hyphal cells.

Fermentation of glucose is negative. *D*-glucose, galactose (weak), sorbose (weak), *N*-acetyl glucosamine, ribose, xylose, *L*-arabinose, *D*-arabinose (weak), *L*-rhamnose, sucrose, maltose, *α*-*α*-trehalose (weak), methyl-*α*-*D*-glucoside (weak), cellobiose, salicin, melibiose (weak), lactose, raffinose, melizitose, glycerol, erythritol, ribitol, glucitol, mannitol, galactitol (weak), *myo*-inositol, *D*-glucono-1,5-lactone, *D*-gluconate (weak), *D*-glucuronate, *D*-galacturonic acid (weak), *DL*-lactate, succinate, citrate, ethanol, and xylitol are assimilated, but inulin, soluble starch, and methanol are not assimilated. Ammonium sulfate, ethylamine hydrochloride, *L*-lysine, cadaverine, and creatine are assimilated as sole nitrogen sources. Potassium nitrate and sodium nitrite are not assimilated. After 3 weeks, black sediment formed in *D*-glucose, *D*-galactose, *L*-sorbose, *N*-acetyl glucosamine, *D*-ribose, *D*-xylose, *L*-arabinose, *D*-arabinose, *L*-rhamnose, sucrose, maltose, *α*-*α*-trehalose, methyl-*α*-*D*-glucoside, cellobiose, salicin, melibiose, lactose, raffinose, melizitose, glycerol, erythritol, ribitol, *D*-glucitol, *D*-mannitol, galactitol, *myo*-inositol, *D*-glucono-1,5-lactone, *D*-gluconate, *D*-glucuronate, *D*-galacturonic acid, *DL*-lactate, succinate, citrate, ethanol, and xylitol.

##### Cardinal temperatures for growth.

Minimum 10 °C, optimum in the range of 25 °C to 30 °C, maximum 37 °C. No growth at 4 °C.

##### Additional strains examined.

THAILAND • Phayao Province, Mueang District, isolated from Chinese ixora flower (*Ixora chinensis*; *Rubiaceae*, *Gentianales*), Aug 2024, P. Kodchasee, C. Senwanna, J. Kumla and N. Suwannarach, living culture = SDBR-CMU846; isolated from holy basil flower (*Ocimum tenuiflorum*; *Lamiaceae*; *Lamiales*), Aug 2024, P. Kodchasee, C. Senwanna, J. Kumla and N. Suwannarach, living culture = SDBR-CMU854.

##### Habitat and distribution.

Fowers of *Ixora chinensis*, *Melampodium divaricatum*, and *Ocimum tenuiflorum*. Known only from northern Thailand.

##### Notes.

The multilocus phylogenetic tree (Fig. 2) indicated that *A. saisamorniae* strains SDBR-CMU846, SDBR-CMU848 (ex-type), and SDBR-CMU854 formed a well-supported monophyletic lineage (100% MLBS and 0.95 BIPP) and were sister to *A. savitreeae* (72% MLBS and 0.94 BIPP). The ITS and LSU sequences of *A. saisamorniae* showed 100% similarity to those of *A. savitreeae*, whereas the *rpb2*, *tef1*, and *tub* sequences exhibited similarities of 99.90%, 94.74%, and 99.75%, respectively. A comparison of nucleotides in the *tef1* sequence showed that *A. saisamorniae* differed by 5.3% (15/285 bp, including gaps). Morphologically, colonies of *A. saisamorniae* on MEA were pale orange to yellowish brown at the center with a grayish-yellow margin, and those on OA were yellowish white with a light-gray margin, whereas colonies of *A. savitreeae* on MEA were orange white, and those on OA were pale yellow with a light brownish-gray margin. In terms of growth temperature, *A. saisamorniae* was capable of growing at 37 °C, whereas *A. savitreeae* was unable to grow at this temperature. Furthermore, the PHI test supported the genetic distinctiveness of *A. saisamorniae* and *A. savitreeae* (Fig. 3), indicating that they represent different species. Physiologically, *A. saisamorniae* produced a black sediment during carbon assimilation, whereas *A. savitreeae* produced brown sediment (Table 1). In addition, *A. savitreeae* is characterized by the production of light pink pigmentation on *D*-glucose, *D*-xylose, erythritol, *L*-arabinose, melezitose, raffinose, and sucrose, whereas *A. saisamorniae* does not produce pigmentation (Table 1). Thus, *A. saisamorniae* was introduced as a new species.

#### Aureobasidium savitreeae

Taxon classificationFungiDothidealesSaccotheciaceae

Senwanna, Kodchasee, J. Kumla & N. Suwannar. sp. nov.

B693E14C-1FEA-5479-9B47-9EEDD56F0808

861973

Fig. 23

##### Etymology.

Named in honor of Thai mycologist Savitree Limtong for her contributions to Thai Mycology.

**Figure 23.**
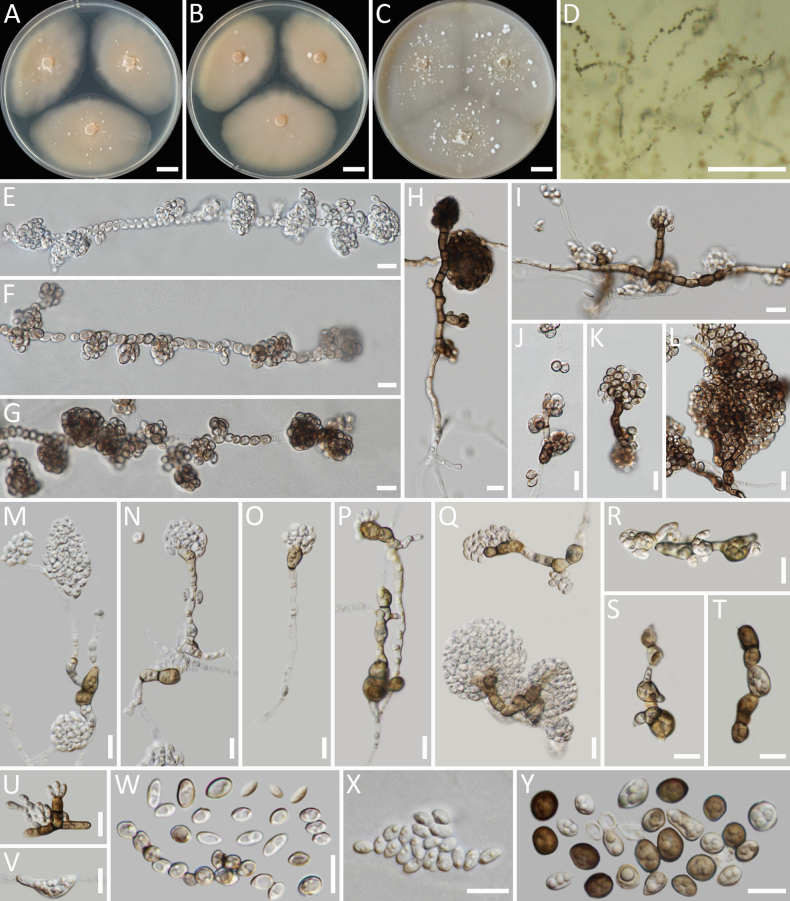
*Aureobasidium savitreeae* (SDBR-CMU824, ex-type). **A–C** colonies on PDA, MEA, and OA, respectively, after 14 d at 25 °C; **D** close-up of an immersed hyphal-tip colony on PDA after 14 d; **E–G** endoconidia and hyphae with groups of conidia; **H, J–L** conidiogenous apparatus; **I** conidiogenous cells integrated into vegetative hyphae with hyaline conidia; **M–Q, U***Hormonema*-like conidiogenous cells with hyaline conidia; **R–T, V** melanized hyphae developing into chlamydospores; **W** conidia; **X** conidia produced from melanized hyphae; **Y** chlamydospores. Scale bars: 1 cm (**A–C**); 500 μm (**D**); 10 μm (**E–Y**).

##### Type.

THAILAND • Chiang Mai Province, Mueang District, Chang Phueak, isolated from confederate vine flower (*Antigonon leptopus*; *Polygonaceae*, *Caryophyllales*), Jul 2024, P. Kodchasee, C. Senwanna, J. Kumla and N. Suwannarach, ***holotype*** = CMUB40132 (preserved in metabolically inactive state), living culture (ex-type) = SDBR-CMU824 = GMBCC2498 = TBRC 21791.

##### Cultural characteristics.

After 14 days of incubation at 25 °C in the dark, colonies on PDA reaching 45–55 mm diam., surface flat, spreading with filiform, entire margin, velvety, pale yellow (2A3); on MEA reaching 47–56 mm diam., surface flat, spreading with filiform, entire margin, orange white (5A2); on OA 65–70 mm diam., surface flat, spreading with entire margin, fluffy, pale yellow (2A3), with light brownish gray (4E2) at the margin.

##### Description.

***Anamorph****in vitro* dematiaceous hyphomycetes. ***Vegetative hyphae*** 2.3–5.5(–6.8) μm wide, hyaline to dark brown, branched, septate, smooth, thin-walled, getting dark melanized and thicker, constricted at the septa with age. ***Conidiogenous cells*** (6.3–)8.0–17.5(–20) × (3.3–)4.0–5.5(–6.4) μm (*x̄* = 12.5 × 4.6 μm, *n* = 25), undifferentiated, intercalary, terminal, producing conidia percurrently from indistinct, short lateral denticles, giving rise to conidia that aggregate in a mucoid mass, or differentiated on hyphae, occasionally elongated, solitary, hyaline to dark brown. ***Conidia*** (3.5–)5.2–6.8(–9.1) × (2.2–)3.0–4.5(–6.5) μm (*x̄* = 6.0 × 3.7 μm, *n* = 200), fusoid, obovoid to ellipsoidal, hyaline to light brown, becoming brown melanized with age, aseptate, occasionally with an indistinct truncated base and a hilum, guttulate, smooth-walled. ***Chlamydospores*** (4.4–)5.4–9.0(–11.8) × 2.6–7.6 μm (*x̄* = 7.2 × 5.5 μm, *n* = 100), frequent produced in aerial hyphae, mostly intercalary, solitary or in branched chains, ellipsoidal, globose to subglobose, pigmented, light to dark brown, smooth-, thick-walled, septate, constricted near the septa. ***Endoconidia (***3.8–)4.9–7.2(–8.0) × (2.2–)3.0–4.8(–5.5) μm (*x̄* = 6.1 × 3.9 μm, *n* = 50), obovoid to ellipsoidal, hyaline, occasionally seen in intercalary hyphal cells.

Fermentation of glucose is negative. *D*-glucose, galactose (weak), sorbose (weak), *N*-acetyl glucosamine (weak), ribose (weak), xylose, *L*-arabinose, *D*-arabinose (weak), *L*-rhamnose, sucrose, maltose (weak), *α*-*α*-trehalose, methyl-*α*-*D*-glucoside, cellobiose, salicin (weak), melibiose (weak), lactose (weak), raffinose, melizitose, glycerol (weak), erythritol (weak), ribitol (weak), glucitol, mannitol (weak), galactitol, *myo*-inositol (weak), *D*-glucono-1,5-lactone (weak), *D*-gluconate (weak), *D*-glucuronate (weak), *D*-galacturonic acid (weak), *DL*-lactate, succinate, citrate, ethanol (weak), and xylitol (weak) are assimilated, but inulin, soluble starch, and methanol are not assimilated. Ammonium sulfate, ethylamine hydrochloride, *L*-lysine, cadaverine, and creatine are assimilated as sole nitrogen sources. Potassium nitrate and sodium nitrite are not assimilated. After 3 weeks, light pink pigmentation and brown sediment were observed in *D*-glucose, *D*-xylose, *L*-arabinose, sucrose, raffinose, melizitose, and erythritol. Brown sediment formed in *D*-galactose, *L*-sorbose, *N*-acetyl glucosamine, *D*-ribose, *D*-arabinose, *L*-rhamnose, maltose, *α*-*α*-trehalose, methyl-*α*-*D*-glucoside, cellobiose, salicin, melibiose, lactose, glycerol, ribitol, *D*-glucitol, *D*-mannitol, galactitol, *myo*-inositol, *D*-glucono-1,5-lactone, *D*-gluconate, *D*-glucuronate, *D*-galacturonic acid, *DL*-lactate, succinate, citrate, ethanol, and xylitol.

##### Cardinal temperatures for growth.

Minimum 10 °C, optimum in the range of 25 °C to 30 °C. No growth at 4 °C and 37 °C.

##### Additional strains examined.

THAILAND • Phayao Province, Mueang District, isolated from oleander flower (*Nerium oleander*; *Apocynaceae*, *Gentianales*), Aug 2024, P. Kodchasee, C. Senwanna, J. Kumla and N. Suwannarach, living culture = SDBR-CMU841.

##### Habitat and distribution.

Flowers of *Antigonon leptopus* and *Nerium oleander*. Known only from northern Thailand.

##### Notes.

The multilocus phylogenetic tree (Fig. 2) indicated that *A. savitreeae* formed a monophyletic clade (100% MLBS and 0.96 BIPP support) and was sister to *A. saisamorniae*. However, differences in molecular data, growth temperature, and morphological and physiological characteristics, as mentioned above, together with the PHI test results (Fig. 3), supported the recognition of *A. savitreeae* and *A. saisamorniae* as distinct species. Therefore, *A. savitreeae* is introduced here as a new species.

#### Aureobasidium siamense

Taxon classificationFungiDothidealesSaccotheciaceae

Senwanna, Kodchasee, J. Kumla & N. Suwannar. sp. nov.

5AA2FBB3-9CC8-55A7-AAEC-B2876AEFBA63

861974

Fig. 24

##### Etymology.

Refers to Siam (the former name of Thailand), where the new species was found.

**Figure 24.**
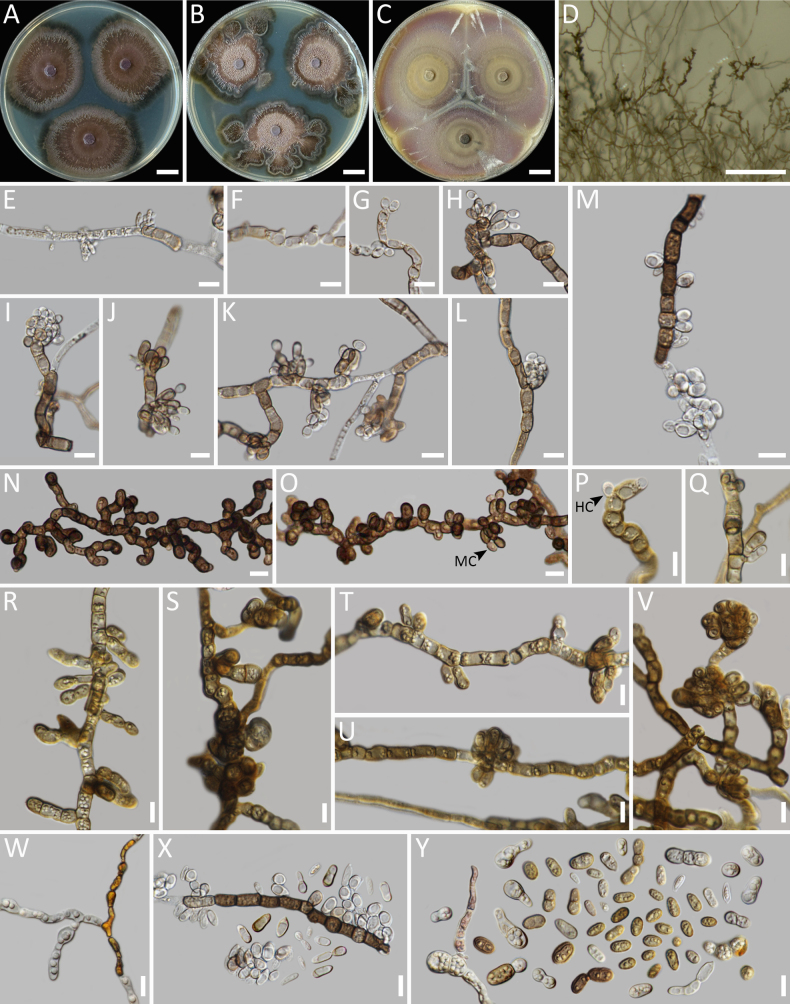
*Aureobasidium siamense* (SDBR-CMU847, ex-type). **A–C** colonies on PDA, MEA, and OA, respectively, after 14 d at 25 °C; **D** close-up of an immersed hyphal-tip colony on PDA after 14 d; **E** hyphae with groups of hyaline conidia; **F** hyphae with lateral pegs; **G–M** conidiogenous apparatus; **N, O** melanized hyphae developing into chlamydospores; **P–V** melanized hyphae with groups of hyaline conidia (HC) and melanized conidia (MC); **W** development of melanized hyphae; **X** conidia; **Y** melanized conidia. Scale bars: 1 cm (**A–C**); 500 μm (**D**); 10 μm (**E–Y**).

##### Type.

THAILAND • Phayao Province, Mueang District, isolated from butter daisy flower (*Melampodium divaricatum*; *Asteraceae*, *Asterales*), Jul 2024, P. Kodchasee, C. Senwanna, J. Kumla and N. Suwannarach, ***holotype*** = CMUB40127 (preserved in metabolically inactive state), living culture (ex-type) = SDBR-CMU847 = GMBCC2504 = TBRC 21792.

##### Cultural characteristics.

After 14 days of incubation at 25 °C in the dark, colonies on PDA reaching 45–53 mm diam., surface flat, spreading, round with radiating margin, velvety, shiny, light brown (6D4) to brown (6E8), with brownish gray (4E2) to black at the margin; on MEA reaching 48–59 mm diam., surface flat, spreading with lobate margin, velvety, shiny, wrinkled, light brown (6D4) to brown (6E8) or grayish brown (6E3), with brownish gray (4E2) to black at the margin; on OA 60–69 mm diam., surface flat, spreading with entire margin, velvety, gradient from light to dark grayish yellow (4B4), with grayish brown (6E3) to dull red (11C4) at the margin.

##### Description.

***Anamorph****in vitro* dematiaceous hyphomycetes. ***Vegetative hyphae*** 4.0–8.0(–10.2) μm wide, hyaline to dark brown, branched, septate, smooth, thin-walled, getting darker and thicker, constricted at the septa with age. ***Conidiogenous cells*** (8.9–)9.5–14.8(–16.1) × 4.8–6.0(–7.2) μm (*x̄* = 11.9 × 6.2 μm, *n* = 25), undifferentiated intercalary, terminal, producing conidia percurrently from indistinct lateral denticles, giving rise to conidia that aggregate in a mucoid mass, or differentiated from hyphae, occasionally elongated, solitary, hyaline to light brown, becoming golden brown to dark brown with age. ***Conidia*** (5.1–)7.4–11.0(–16.2) × (2.1–)3.6–5.9(–8.2) μm (*x̄* = 9.3 × 5.0 μm, *n* = 200), obovoid to ellipsoidal, hyaline to light brown, aseptate, occasionally with an indistinct truncated base and a hilum, guttulate, smooth-walled, becoming light brown to brown with age, 0–1-septate. ***Chlamydospores*** 6.6–10.6(–12.7) μm wide (*x̄* = 9.0 μm, *n* = 25), produced in aerial hyphae, mostly intercalary, solitary or in branched chains, subglobose, dark brown, smooth-, thick-walled, septate, constricted near the septa. ***Endoconidia*** absent.

Fermentation of glucose is negative. *D*-glucose, galactose, sorbose, *N*-acetyl glucosamine, ribose, xylose, *L*-arabinose, *D*-arabinose, *L-*rhamnose, sucrose, maltose, *α-α-*trehalose, methyl-*α*-*D*-glucoside (weak), cellobiose (weak), salicin, melibiose (weak), lactose (weak), raffinose (weak), melizitose (weak), glycerol, erythritol (weak), ribitol, glucitol, mannitol (weak), galactitol, *myo*-inositol (weak), *D*-glucono-1,5-lactone, *D*-gluconate (weak), *D*-glucuronate (weak), *D*-galacturonic acid (weak), *DL*-lactate (weak), succinate (weak), citrate (weak), ethanol (weak), and xylitol (weak) are assimilated, but inulin, soluble starch, and methanol are not assimilated. Ammonium sulfate, ethylamine hydrochloride, *L*-lysine, cadaverine, and creatine are assimilated as sole nitrogen sources. Potassium nitrate and sodium nitrite are not assimilated. After 3 weeks, light brown pigmentation and dark brown sediment were observed in *D*-glucono-1,5-lactone. Dark brown sediment formed in *D*-galactose, *L*-sorbose, and salicin, whereas light brown sediment presented in *D*-glucose. Back sediment formed in *D*-ribose, *D*-arabinose, and *D*-gluconate, while white sediment preformed in *N*-ace­tyl glucosamine, *D*-xylose, *L*-arabinose, *L*-rhamnose, sucrose, maltose, *α*-*α*-trehalose, methyl-*α*-*D*-glucoside, cellobiose, melibiose, lactose, raffinose, melizitose, glycerol, erythritol, ribitol, *D*-glucitol, *D*-mannitol, galactitol, *myo*-inositol, *D*-glucuronate, *D*-galacturonic acid, *DL*-lactate, succinate, citrate, ethanol, and xylitol.

##### Cardinal temperatures for growth.

Minimum 10 °C, optimum in the range of 25 °C to 30 °C. No growth at 4 °C and 37 °C.

##### Additional strains examined.

THAILAND • Phayao Province, Mueang District, isolated from Egyptian star cluster flower (*Pentas lanceolata*; *Rubiaceae*, *Gentianales*), Aug 2024, P. Kodchasee, C. Senwanna, J. Kumla and N. Suwannarach, living culture = SDBR-CMU837.

##### Habitat and distribution.

Flowers of *Melampodium divaricatum* and *Pentas lanceolata*. Known only from northern Thailand.

##### Notes.

Phylogenetic analyses showed that *A. siamense* strains SDBR-CMU837 and SDBR-CMU847 (ex-type) formed a distinct lineage with strong support (100% MLBS and 0.99 BIPP) and were sister to *A. motuoense* (Fig. 2). The ITS and LSU sequences of *A. siamense* showed 98.77% and 100% similarity, respectively, to those of *A. motuoense* strains XZY411-4 (ex-type), E26-4, E31-4, and E82-2. However, the *rpb2*, *tef1*, and *tub* sequences could not be compared because sequences of *A. motuoense* were unavailable. Morphologically, *A. siamense* is distinguishable from *A. motuoense* based on differences in colony characteristics. Colonies of *A. siamense* on PDA are light brown to brown with brownish-gray to black margins; those on MEA are light brown to grayish brown with brownish-gray to black margins; and those on OA are light to dark grayish yellow with grayish-brown to dull-red margins. In contrast, colonies of *A. motuoense* on PDA are dark brown with black zones; those on MEA are brownish olivaceous; and those on OA are pitch black. *Aureobasidium siamense* is distinguished from *A. motuoense* by the production of endoconidia. In addition, *A. siamense* differs from *A. motuoense* in having longer conidiogenous cells [(8.9–)9.5–14.8(–16.1) × 4.8–6.0(–7.2) μm vs. 6.4–12.2 × 3.6–4.5 μm] (Wu et al. 2023). However, because of the lack of available physiological characteristics of *A. motuoense*, a direct comparison between *A. siamense* and *A. motuoense* could not be made. Thus, *A. siamense* is described herein as a new species based on morphological and multilocus phylogenetic analyses.

#### Aureobasidium tremulum

Taxon classificationFungiDothidealesSaccotheciaceae

Inamdar, Roh. Sharma & Adhapure, Persoonia 42: 383 (2019)

08D4B103-1EEB-500C-8660-BFA2A377FC54

829941

Fig. 25

##### Cultural characteristics.

After 14 days of incubation at 25 °C in the dark, colonies on PDA reaching 27–30 mm diam., surface flat, spreading with irregular, lobate margin, orange white (5A2), light gray (5B2) at the margin; on MEA reaching 24–35 mm diam., surface flat, spreading with irregular margin, velvety, purplish gray (14D2) in outer ring, brownish gray (4E2) towards to the center; on OA 30–50 mm diam., surface flat, spreading with irregular margin, velvety, aerial mycelium sparse, floccose, light gray (4B1) in outer ring, grayish brown (5E3) towards to the center, yellowish brown (5E8) at center.

**Figure 25.**
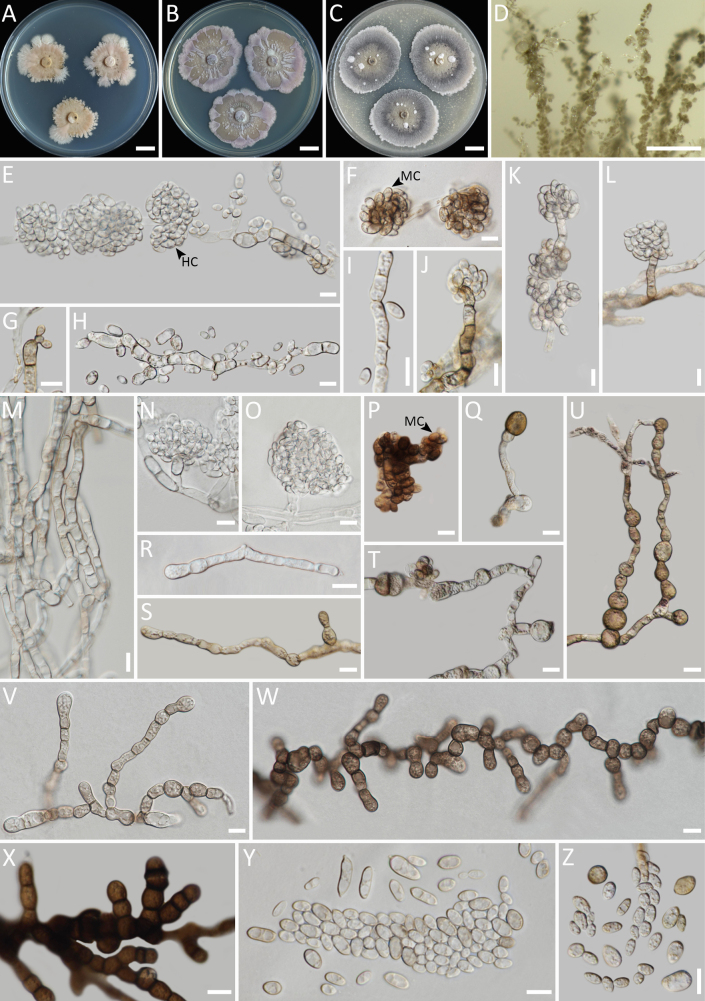
*Aureobasidium tremulum* (SDBR-CMU816). **A–C** colonies on PDA, MEA, and OA, respectively, after 14 d at 25 °C; **D** close-up of an immersed hyphal-tip colony on PDA after 14 d; **E, F** hyphae with groups of hyaline conidia (HC) and melanized conidia (MC); **G** conidiogenous apparatus; **H–J** hyphae with lateral pegs; **K, L, N, O** conidiogenous cells integrated into vegetative hyphae with hyaline conidia; **M** hyphal anastomosis; **P** melanized hyphae with groups of melanized conidia (MC); **Q** germinated conidia developing into chlamydospores; **R–X** hyphae and melanized hyphae developing into chlamydospores; **Y** conidia; **Z** conidia and chlamydospores. Scale bars: 1 cm (**A–C**); 500 μm (**D**); 10 μm (**E–Z**).

##### Description.

***Anamorph****in vitro* dematiaceous hyphomycetes. ***Vegetative hyphae*** (2.4–)4.0–5.8(–7.6) μm wide, mostly hyaline, branched, septate, smooth-, thin-walled, getting darker and thicker, constricted at the septa, minutely echinulate with age, anastomosis observed. ***Conidiogenous cells*** (8.5–)9.5–17.5(–20.6) × (3.7–)5.2–7.0(–8.5) μm (*x̄* = 13.9 × 6.1 μm, *n* = 25), undifferentiated, intercalary, rarely terminal, or producing conidia percurrently from short lateral denticles, giving rise to conidia that aggregate in a mucoid mass, or differentiated from hyphae. ***Conidia*** (4.6–)6.8–10.0(–17.3) × (2.8–)3.9–5.5(–7.2) μm (*x̄* = 8.1 × 4.5 μm, *n* = 170), fusoid, obovoid to ellipsoid, or oval, or globose, hyaline, becoming light brown to brown with age, aseptate, occasionally with an indistinct truncated base and a hilum, minutely echinulate, budding occasionally observed. ***Chlamydospores*** (7.0–)8.5–13.0(–16) μm wide (*x̄* = 10.6 μm, *n* = 30), frequent produced in aerial hyphae, mostly intercalary, solitary or in branched chains, sub globose to ellipsoidal, pigmented, light to dark brown, smooth-, thick-walled, septate, constricted near the septa. ***Endoconidia*** absent.

##### Cardinal temperatures for growth.

Minimum 10 °C, optimum in the range of 25 °C to 30 °C. No growth at 4 °C and 37 °C.

##### Strains examined.

THAILAND • Chiang Mai Province, Mueang District, Chang Phueak, isolated from Spanish needle flower (*Bidens pilosa*; *Asteraceae*, *Asterales*), Jul 2024, P. Kodchasee, C. Senwanna, J. Kumla and N. Suwannarach, living culture = SDBR-CMU814; isolated from Carey rose flower (*Rosa hybrida* ‘AUSweather’; *Rosaceae*, *Rosales*), Jul 2024, P. Kodchasee, C. Senwanna, J. Kumla and N. Suwannarach, living culture = SDBR-CMU820; isolated from holy basil flower (*Ocimum tenuiflorum*; *Lamiaceae*; *Lamiales*), Aug 2024, P. Kodchasee, C. Senwanna, J. Kumla and N. Suwannarach, living culture = SDBR-CMU856); Chiang Mai Province, Mueang District, Suthep, isolated from American cassia flower (*Senna spectabilis*; *Fabaceae*, *Fabales*), Jul 2024, P. Kodchasee, C. Senwanna, J. Kumla and N. Suwannarach, living culture = SDBR-CMU816 and SDBR-CMU834; Phayao Province, Mueang District, isolated from tropical hibiscus flower (*Hibiscus rosa-sinensis*; *Malvaceae*, *Malvales*), Aug 2024, P. Kodchasee, C. Senwanna, J. Kumla and N. Suwannarach, living culture = SDBR-CMU838 F277.

##### Habitat and distribution.

From laboratory contaminants in India (Crous et al. 2019); flowers of *Bidens pilosa*, *Hibiscus rosa-sinensis*, *Ocimum tenuiflorum*, *Rosa hybrida* ‘AUSweather,’ and *Senna spectabilis* in Thailand (this study).

##### Notes.

Phylogenetically, strains SDBR-CMU814, SDBR-CMU816, SDBR-CMU820, SDBR-CMU834, SDBR-CMU838, and SDBR-CMU856 belonged to the *A. tremulum* clade (Fig. 2), which formed a sister clade to *A. oroxyli*. The differences in sequence data and morphological characteristics between *A. tremulum* and *A. oroxyli* are mentioned above. However, the morphological characteristics of the strains could not be compared with those of the type strain of *A. tremulum* because it is known only from its yeast form (Crous et al. 2019). Comparisons of the ITS, LSU, *rpb2*, and *tub* sequences among *A. tremulum* strains SDBR-CMU834 and SDBR-CMU838 and strains SDBR-CMU816 and SDBR-CMU820 showed no significant differences. Although strains SDBR-CMU814 and SDBR-CMU856 formed a sister clade to *A. tremulum* and differed by only 3.2% (9/277 bp, including gaps) in the *tef1* sequence, their morphological characteristics, growth temperature, and the currently available data do not support recognition as a distinct species. Until morphological data and sequences of reliable genes (*rpb2*, *tef1*, and *tub*) from the type strain are obtained and verified, these strains are therefore treated as *A. tremulum*. This study represents the first report of *A. tremulum* in Thailand.

## Discussion

Species identification within the genus *Aureobasidium* is challenging because of extensive morphological variation and overlapping phenotypic characteristics among taxa (de Hoog and Yurlova 1994; Yurlova and de Hoog 1997). Physiological characteristics (e.g., growth temperature profiles and carbon and nitrogen assimilation profiles) and exopolysaccharide structure have also been used to support species identification (Hermanides-Nijhof 1977; Yurlova and de Hoog 1997; Urzì et al. 1999; Prasongsuk et al. 2005; Wu et al. 2023; Jumbam et al. 2025). Molecular approaches have been used for the identification of *Aureobasidium* species. Commonly used barcode regions, such as ITS and LSU, often provide insufficient resolution for species delimitation (da Silva et al. 2024). Genomic analyses later demonstrated that these varieties represent distinct species in the *A. pullulans* group (Gostinčar et al. 2014). Protein-coding genes (e.g., *elo2*, *rpb2*, *tef1*, and *tub*) have therefore been recommended for resolving phylogenetic relationships and delineating species boundaries in *Aureobasidium* (Zalar et al. 2008; Gostinčar et al. 2014; Parra et al. 2023; da Silva et al. 2024; Czachura and Piątek 2025). Consequently, accurate identification requires an integrative approach that combines morphological data with multilocus phylogenetic analyses (da Silva et al. 2024; Jumbam et al. 2025). Moreover, owing to the high morphological and genetic variability among species, genealogical concordance phylogenetic species recognition (GCPSR) and phylogenetic species recognition (PSR) have been applied to resolve cryptic fungal taxa, including *Aureobasidium* (Liu et al. 2016; Hilário et al. 2021; Jumbam et al. 2025). Comparative assessments of species recognition methods demonstrated that GCPSR provides the most effective framework for delimiting species boundaries within *Aureobasidium*. Furthermore, physiological characteristics, particularly carbon assimilation and fermentation profiles, represent useful complementary characteristics that can aid in the discrimination of closely related taxa and support phylogenetically inferred species boundaries (Jumbam et al. 2025).

In this study, 16 novel species and five previously known species of *Aureobasidium*, isolated from 31 flower species in northern Thailand, are described based on morphological and physiological characteristics, growth temperature profiles, and multilocus phylogenetic analyses of ITS, LSU, *rpb2*, *tef1*, and *tub* sequence data. The combined analyses supported the classification of *Aureobasidium* into five groups, namely, the *A. melanogenum*, *A. namibiae*, *A. pullulans*, *A. subglaciale*, and *A. thailandense* groups, and corroborated the placement of the representative strains, consistent with previous phylogenetic studies (Zalar et al. 2008; da Silva et al. 2024; Jumbam et al. 2025). Phylogenetic analyses based on multilocus sequence data revealed that the majority of *Aureobasidium* species obtained in this study clustered within the *A. melanogenum* species group (viz. *A. chiangmaiense*, *A. melanogenum*, *A. musti*, *A. phayaoense*, *A. plumeriae*, *A. pollinicola*, *A. saisamorniae*, *A. savitreeae*, and *A. siamense*) and the *A. thailandense* species group (viz. *A. albui*, *A. anthicola*, *A. castaneae*, *A. florale*, *A. floricola*, *A. florigenum*, *A. lannaense*, and *A. kwanphayaoense*). *Aureobasidium bougainvilleae*, *A. catharanthi*, *A. oroxyli*, and *A. tremulum* formed a distinct, well-supported clade within *Aureobasidium* (Fig. 2). In addition to pairwise DNA sequence comparisons among related species within the *A. melanogenum* and *A. thailandense* groups, this study demonstrates that *Aureobasidium* species can be effectively distinguished based on *rpb2* and *tef1* sequences, which provide high phylogenetic resolution, whereas ITS and *tub* are useful for differentiating certain species. It is therefore recommended that future taxonomic studies of *Aureobasidium* employ a standardized set of molecular markers, including ITS, *rpb2*, *tef1*, and additional informative loci, to ensure consistent species identification and facilitate comprehensive phylogenetic analyses across the genus. Furthermore, the GCPSR, GMYC, and PTP (bPTP, mlbPTP, and mPTP) analyses were implemented for species delimitation within an integrative taxonomic framework combining multilocus phylogenetic analyses. The GCPSR and GMYC approaches delimited species boundaries that were largely congruent with well-supported clades, thereby providing independent support for species-level differentiation, particularly within the *A. melanogenum* and *A. thailandense* groups. However, GMYC tended to oversplit lineages, consistent with previous studies suggesting that it may overestimate species numbers (Fujisawa and Barraclough 2013; Talavera et al. 2013). In contrast, discrepancies among the mlbPTP, bPTP, and mPTP delimitation methods were observed in closely related lineages within species complexes (i.e., the *A. melanogenum*, *A. namibiae*, *A. pullulans*, and *A. thailandense* groups), in agreement with Jumbam et al. (2025). These inconsistencies likely reflect evolutionary processes such as incomplete lineage sorting or ongoing gene flow, which can obscure species boundaries. Consequently, reliance on a single delimitation method may result in the over- or underestimation of species diversity. In the present study, GCPSR analyses were considered the most suitable approach for species delimitation, consistent with the findings of Jumbam et al. (2025). The phylogenetic structure observed in this study highlights the high diversity and complex evolutionary history of *Aureobasidium*. The presence of multiple well-supported clades, combined with the detection of numerous putative new species, suggests that species diversity in this genus remains underestimated. Although most species boundaries are well supported, some groups exhibit complex patterns that highlight the need for additional data (e.g., population-level sampling, ecological traits, or genomic analyses) to fully resolve species limits. Physiological characteristics provided valuable supporting evidence for distinguishing closely related species within the genus *Aureobasidium*. In particular, diagnostic differences in carbon assimilation profiles, pigmentation development, and sediment formation during substrate utilization proved useful for differentiating the newly described species from their closest phylogenetic relatives. These findings are consistent with the recommendations of Jumbam et al. (2025), who emphasized that physiological characterization, particularly carbon assimilation and fermentation tests, should complement morphological and multilocus phylogenetic analyses in a polyphasic approach to species delimitation within *Aureobasidium*. Therefore, based on the findings of the present study, the adoption of a polyphasic approach for species identification and delimitation in *Aureobasidium* is recommended by integrating multilocus phylogenetic, morphological, and physiological data. This integrative framework provides a more robust and reliable basis for recognizing species boundaries, particularly among morphologically similar and cryptic species. Future studies should incorporate broader taxon sampling and additional gene loci to improve the resolution of species boundaries within *Aureobasidium*, alongside the integration of genomic data, multiple species-delimitation approaches, and ecological and physiological data.

*Aureobasidium* is a ubiquitous and cosmopolitan genus isolated from diverse substrates worldwide (Zalar et al. 2008; Zajc et al. 2020, 2022; Lee et al. 2021; Wang et al. 2022b; da Silva et al. 2024; Jumbam et al. 2025). However, species within the genus exhibit either broad or restricted distributions, reflecting ecological differentiation rather than random variation within populations (Zalar et al. 2008; Gostinčar et al. 2014, 2019; Černoša et al. 2021; Zajc et al. 2022). This difference may reflect distinct evolutionary strategies and ecological adaptations within the genus (Jia et al. 2019; Akbari et al. 2024; Yang et al. 2024). For example, *A. melanogenum* and *A. pullulans* are highly versatile and polyextremotolerant species known for their ability to thrive in diverse and extreme environments, reflecting their remarkable ecological adaptability (Zalar et al. 2008; Gostinčar et al. 2019; Zajc et al. 2020; Černoša et al. 2021). Owing to these adaptations, genome-based studies of these species from different environments have been conducted to investigate their ecological versatility, biotechnological and metabolic potential, stress tolerance, and taxonomic features (Gostinčar et al. 2014; Černoša et al. 2021). Notably, these studies have revealed contrasting population structures. *Aureobasidium pullulans* exhibits high levels of recombination, which may obscure species boundaries and complicate phylogenetic inference, consistent with its flexible lifestyle and frequent exposure to diverse environmental conditions (Gostinčar et al. 2014). In contrast, *A. melanogenum*, which is associated with more restricted habitats, shows little to no evidence of recombination within the species, a pattern also observed in *A. subglaciale* (Zajc et al. 2022). As mentioned above, *Aureobasidium* species, particularly *A. pullulans* and *A. thailandense*, have frequently been isolated from floral habitats (Temple et al. 2020; Félix et al. 2021; Di Francesco et al. 2023; Agarbati et al. 2024). The frequent occurrence and high diversity of *Aureobasidium* species in floral environments can be attributed to the combined effects of ecological suitability, physiological adaptability, and dispersal dynamics (Temple et al. 2020; Morris et al. 2020; Ruraż et al. 2023). Flowers represent nutrient-rich yet selective microhabitats, driven by sugar-rich nectar, fluctuating environmental conditions, and short life spans, all of which favor fast-growing and stress-tolerant microorganisms (Ushio et al. 2015; Vannette et al. 2021; Niu et al. 2022). Therefore, microorganisms associated with flowers are adapted for survival through traits such as rapid colonization and persistence under osmotic and desiccation stress (Herrera et al. 2008; Mittelbach et al. 2015; Morris et al. 2020). *Aureobasidium* exhibits a wide range of stress-tolerance traits that enable its persistence on exposed plant surfaces, including osmotolerance in high-sugar environments, resistance to ultraviolet radiation via melanin production, and the secretion of extracellular polysaccharides that facilitate adhesion and biofilm formation (Gostinčar et al. 2014; Di Francesco et al. 2023; Rensink et al. 2024). Furthermore, floral structures serve as sites of microbial exchange, where pollinators facilitate the transfer of microorganisms among plant hosts, thereby increasing species mixing and promoting microbial diversity (Morris et al. 2020; Cullen et al. 2021; Lignon et al. 2025). This process promotes the continual dispersal of *Aureobasidium* from diverse reservoirs into floral environments, consistent with its broad ecological amplitude and generalist life strategy (Andrews and Harris 2000; Glushakova and Chernov 2007; Zalar et al. 2008), and likely contributes to its persistence and elevated diversity within these habitats.

This study highlights that flowers represent an important source for investigating the diversity of *Aureobasidium* species and emphasizes the need for further investigations. The adaptive and survival mechanisms of flower-associated *Aureobasidium* identified in this study warrant further investigation. Furthermore, future studies should employ different culture media and culture-independent methods to comprehensively characterize the diversity of *Aureobasidium* in floral environments, including seasonal variations, floral species, and floral distribution patterns. In addition, integrating genome-based approaches, broader geographic sampling, and ecological investigations will be essential to elucidate the diversity, distribution, and functional roles of flower-associated *Aureobasidium* species.

## Conclusion

This study demonstrates that flowers in northern Thailand harbor unexpectedly high *Aureobasidium* diversity (21 species from 31 plant species), highlighting floral habitats as important and previously underexplored reservoirs of fungal diversity in tropical ecosystems. The discovery of 16 novel species, together with five known species and four species recorded for the first time in Thailand, substantially expands current knowledge of the taxonomy, diversity, and geographic distribution of *Aureobasidium*. The application of a polyphasic approach integrating multilocus phylogenetic analyses, morphological and physiological characteristics, growth temperature profiles, and GCPSR supported by PHI tests provides robust species delimitation within this complex group, particularly within the *A. melanogenum* and *A. thailandense* groups. These findings significantly enhance current knowledge of *Aureobasidium* in tropical regions and underscore the ecological significance of flowers as key fungal niches, highlighting the need for further systematic surveys of flower-associated mycobiota in tropical ecosystems.

## Supplementary Material

XML Treatment for Aureobasidium albui

XML Treatment for Aureobasidium anthicola

XML Treatment for Aureobasidium bougainvilleae

XML Treatment for Aureobasidium castaneae

XML Treatment for Aureobasidium catharanthi

XML Treatment for Aureobasidium chiangmaiense

XML Treatment for Aureobasidium florale

XML Treatment for Aureobasidium floricola

XML Treatment for Aureobasidium florigenum

XML Treatment for Aureobasidium kwanphayaoense

XML Treatment for Aureobasidium lannaense

XML Treatment for Aureobasidium melanogenum

XML Treatment for Aureobasidium musti

XML Treatment for Aureobasidium oroxyli

XML Treatment for Aureobasidium phayaoense

XML Treatment for Aureobasidium plumeriae

XML Treatment for Aureobasidium pollinicola

XML Treatment for Aureobasidium saisamorniae

XML Treatment for Aureobasidium savitreeae

XML Treatment for Aureobasidium siamense

XML Treatment for Aureobasidium tremulum

## Additional information

### Artificial Intelligence (AI) use

The authors accept full responsibility for the content of the manuscript, including the disclosure of any use of AI.

No AI tools were used in the preparation of this manuscript.

### Adherence to national and international regulations

All the fungal strains used in this study have been legally obtained, respecting the Convention on Biological Diversity (Rio Convention).

### Author ORCIDs

C. Senwanna https://orcid.org/0000-0002-1008-4514

P. Kodchasee https://orcid.org/0000-0003-0432-4910

C. Kathongthung https://orcid.org/0009-0007-0517-6063

M.C. Samarakoon https://orcid.org/0000-0002-4815-125X

P. Khunnamwong https://orcid.org/0000-0001-7536-929X

J. Kumla https://orcid.org/0000-0002-3673-6541

N. Suwannarach https://orcid.org/0000-0002-2653-1913

### Data availability

The DNA sequence data obtained in this study have been deposited in GenBank (https://www.ncbi.nlm.nih.gov/genbank/). The alignments have been deposited in TreeBASE (https://www.treebase.org/). Data are available in the main text or the supplementary information.

## Supplementary materials

Supplementary material 1 Single locus phylogenetic trees Data type docx https://binary.pensoft.net/file/1732658This dataset is made available under the Open Database License (http://opendatacommons.org/licenses/odbl/1.0/). The Open Database License (ODbL) is a license agreement intended to allow users to freely share, modify, and use this Dataset while maintaining this same freedom for others, provided that the original source and author(s) are credited. Chanokned Senwanna, Pratthana Kodchasee, Chirayut Kathongthung, Milan C. Samarakoon, Pannida Khunnamwong, Jaturong Kumla, Nakarin Suwannarach

Supplementary material 2 Sampling location, flower species, strain, and species identification based on sequence similarity of *Aureobasidium* in this study Data type docx https://binary.pensoft.net/file/1732659This dataset is made available under the Open Database License (http://opendatacommons.org/licenses/odbl/1.0/). The Open Database License (ODbL) is a license agreement intended to allow users to freely share, modify, and use this Dataset while maintaining this same freedom for others, provided that the original source and author(s) are credited. Chanokned Senwanna, Pratthana Kodchasee, Chirayut Kathongthung, Milan C. Samarakoon, Pannida Khunnamwong, Jaturong Kumla, Nakarin Suwannarach

Supplementary material 3 Fungal name, strain numbers and corresponding GenBank accession numbers of the taxa used in the phylogenetic analyses Data type docx https://binary.pensoft.net/file/1732660This dataset is made available under the Open Database License (http://opendatacommons.org/licenses/odbl/1.0/). The Open Database License (ODbL) is a license agreement intended to allow users to freely share, modify, and use this Dataset while maintaining this same freedom for others, provided that the original source and author(s) are credited. Chanokned Senwanna, Pratthana Kodchasee, Chirayut Kathongthung, Milan C. Samarakoon, Pannida Khunnamwong, Jaturong Kumla, Nakarin Suwannarach
