# Supplementary material for: Unexpected diversity of flower-associated Aureobasidium reveals sixteen novel species from northern Thailand
Source: IMA Fungus. 2026 Aug 3;17:e188009. doi: 10.3897/imafungus.17.188009 (PMC13458542; doi:10.3897/imafungus.17.188009)
Supplement: Supplementary file 1 [file imafungus-17-e188009-s001.docx]

**Figure S1.** Phylogenetic tree generated from maximum likelihood analysis of *Aureobasidium* based on a combined ITS sequence dataset. Bootstrap values ≥ 50% MLBS and Bayesian posterior probabilities (BIPP) ≥ 0.90 are shown above nodes and defined as ML/PP. The tree is rooted to *Delphinella strobiligena* CBS 735.71 and *Sydowia polyspora* CBS 750.71. The fungal strains obtained in this study are blue. Ex-type strains are in bold. The scale bar represents the expected number of nucleotide substitutions per site.

**Figure S1.** Continued.

**Figure S2.** Phylogenetic tree generated from maximum likelihood analysis of *Aureobasidium* based on a combined LSU sequence dataset. Bootstrap values ≥ 50% MLBS and Bayesian posterior probabilities (BIPP) ≥ 0.90 are shown above nodes and defined as ML/PP. The tree is rooted to *Delphinella strobiligena* CBS 735.71 and *Sydowia polyspora* CBS 750.71. The fungal strains obtained in this study are blue. Ex-type strains are in bold. The scale bar represents the expected number of nucleotide substitutions per site.

**Figure S2.** Continued.

**Figure S3.** Phylogenetic tree generated from maximum likelihood analysis of *Aureobasidium* based on a combined *rpb2* sequence dataset. Bootstrap values ≥ 50% MLBS and Bayesian posterior probabilities (BIPP) ≥ 0.90 are shown above nodes and defined as ML/PP. The tree is rooted to *Sydowia polyspora* CBS 750.71. The fungal strains obtained in this study are blue. Ex-type strains are in bold. The scale bar represents the expected number of nucleotide substitutions per site.

**Figure S4.**  Phylogenetic tree generated from maximum likelihood analysis of *Aureobasidium* based on a combined *tef1* sequence dataset. Bootstrap values ≥ 50% MLBS and Bayesian posterior probabilities (BIPP) ≥ 0.90 are shown above nodes and defined as ML/PP. The tree is rooted to *Pseudoseptoria obscura* CBS 135103. The fungal strains obtained in this study are blue. Ex-type strains are in bold. The scale bar represents the expected number of nucleotide substitutions per site.

**Figure S5.**  Phylogenetic tree generated from maximum likelihood analysis of *Aureobasidium* based on a combined *tub* sequence dataset. Bootstrap values ≥ 50% MLBS and Bayesian posterior probabilities (BIPP) ≥ 0.90 are shown above nodes and defined as ML/PP. The tree is rooted to *Pseudoseptoria obscura* CBS 135103. The fungal strains obtained in this study are blue. Ex-type strains are in bold. The scale bar represents the expected number of nucleotide substitutions per site.
