# Supplementary material for: Unexpected diversity of flower-associated Aureobasidium reveals sixteen novel species from northern Thailand
Source: IMA Fungus. 2026 Aug 3;17:e188009. doi: 10.3897/imafungus.17.188009 (PMC13458542; doi:10.3897/imafungus.17.188009)
Supplement: Supplementary file 2 [file imafungus-17-e188009-s002.docx]

**Table S1.** Sampling location, flower species, strain, and species identification based on ITS sequence similarity of *Aureobasidium* in this study.

| **Sampling location** | **Flower species** | **Strains** | **Sequence similarity*** | **Species identification** |
| --- | --- | --- | --- | --- |
| Chiang Mai Province | *Alstonia scholaris* | SDBR-CMU864 | *A*. *musti* | *A*. *musti* |
|  |  | SDBR-CMU866 | *A. albui* | *A. albui* |
|  | *Antigonon leptopus* | SDBR-CMU824 | Undescribed species | *A*. *savitreeae* sp. nov. |
|  |  | SDBR-CMU825 | *A*. *musti* | *A*. *musti* |
|  | *Argyranthemum frutescens* | SDBR-CMU861 | Undescribed species | *A*. *pollinicola* sp. nov. |
|  | *Bidens Pilosa* | SDBR-CMU814 | *A*. *tremulum* | *A*. *tremulum* |
|  | *Cheilocostus speciosus* | SDBR-CMU833 | Undescribed species | *A*. *lannaense* sp. nov. |
|  | *Citrus japonica* | SDBR-CMU829 | Undescribed species | *A. floricola* sp. nov. |
|  | *Ehretia microphylla* | SDBR-CMU821 | Undescribed species | *A. anthicola* sp. nov. |
|  | *Exacum affine* | SDBR-CMU818 | Undescribed species | *A. chiangmaiense* sp. nov. |
|  | *Jasminum sambac* | SDBR-CMU817 | *A*. *melanogenum* | *A*. *melanogenum* |
|  | *Jatropha integerrima* | SDBR-CMU840 | Undescribed species | *A. florale* sp. nov. |
|  |  | SDBR-CMU850 | Undescribed species | *A. florale* sp. nov. |
|  | *Lagerstroemia speciosa* | SDBR-CMU863 | Undescribed species | *A*. *lannaense* sp. nov. |
|  | *Nerium oleander* | SDBR-CMU819 | Undescribed species | *A. chiangmaiense* sp. nov. |
|  | *Ocimum tenuiflorum* | SDBR-CMU856 | *A*. *tremulum* | *A*. *tremulum* |
|  | *Oroxylum indicum* | SDBR-CMU822 | Undescribed species | *A. oroxyli* sp. nov. |
|  |  | SDBR-CMU827 | Undescribed species | *A. oroxyli* sp. nov. |
|  | *Plumeria obtusa* | SDBR-CMU826 | Undescribed species | *A*. *plumeriae* sp. nov. |
|  |  | SDBR-CMU836 | Undescribed species | *A*. *plumeriae* sp. nov. |
|  | *Rosa hybrida* | SDBR-CMU820 | *A*. *tremulum* | *A*. *tremulum* |
|  | *Senna spectabilis* | SDBR-CMU816 | *A*. *tremulum* | *A*. *tremulum* |
|  |  | SDBR-CMU834 | *A*. *tremulum* | *A*. *tremulum* |
|  |  | SDBR-CMU835 | *A*. *castaneae* | *A*. *castaneae* |
|  | *Tagetes erecta* | SDBR-CMU865 | Undescribed species | *A*. *florigenum* sp. nov. |
|  | *Tecoma stans* | SDBR-CMU828 | Undescribed species | *A*. *florigenum* sp. nov. |
|  | *Tectona grandis* | SDBR-CMU830 | Undescribed species | *A. florale* sp. nov. |
|  |  | SDBR-CMU831 | Undescribed species | *A. florale* sp. nov. |
|  |  | SDBR-CMU832 | Undescribed species | *A. florale* sp. nov. |
|  | *Thymophylla tenuiloba* | SDBR-CMU862 | Undescribed species | *A. florale* sp. nov. |
| Phayao Province | *Plumeria pudica* | SDBR-CMU851 | Undescribed species | *A. anthicola* sp. nov. |
|  | *Bougainvillea hybrid* | SDBR-CMU858 | Undescribed species | *A*. *bougainvilleae* sp. nov. |
|  |  | SDBR-CMU859 | Undescribed species | *A*. *bougainvilleae* sp. nov. |
|  | *Catharanthus roseus* | SDBR-CMU812 | Undescribed species | *A. catharanthi* sp. nov. |
|  |  | SDBR-CMU813 | Undescribed species | *A*. *pollinicola* sp. nov. |
|  |  | SDBR-CMU842 | Undescribed species | *A. catharanthi* sp. nov. |
|  |  | SDBR-CMU843 | Undescribed species | *A*. *pollinicola* sp. nov. |
|  | *Cnidoscolus aconitifolius* | SDBR-CMU849 | Undescribed species | *A. kwanphayaoense* sp. nov. |
|  | *Hibiscus rosa-sinensis* | SDBR-CMU838 | *A*. *tremulum* | *A*. *tremulum* |
|  | *Ixora chinensis* | SDBR-CMU846 | Undescribed species | *A*. *saisamorniae* sp. nov. |
|  | *Jatropha integerrima* | SDBR-CMU839 | Undescribed species | *A. kwanphayaoense* sp. nov. |
|  | *Melampodium divaricatum* | SDBR-CMU847 | Undescribed species | *A*. *siamense* sp. nov. |
|  |  | SDBR-CMU848 | Undescribed species | *A*. *saisamorniae* sp. nov. |
|  | *Nerium oleander* | SDBR-CMU841 | Undescribed species | *A*. *savitreeae* sp. nov. |
|  | *Ocimum tenuiflorum* | SDBR-CMU844 | Undescribed species | *A. kwanphayaoense* sp. nov. |
|  |  | SDBR-CMU854 | Undescribed species | *A*. *saisamorniae* sp. nov. |
|  |  | SDBR-CMU855 | *A*. *melanogenum* | *A*. *melanogenum* |
|  | *Pentas lanceolata* | SDBR-CMU837 | Undescribed species | *A*. *siamense* sp. nov. |
|  |  | SDBR-CMU852 | Undescribed species | *A*. *phayaoense* sp. nov. |
|  |  | SDBR-CMU853 | Undescribed species | *A. floricola* sp. nov. |
|  | *Plumeria obtuse* | SDBR-CMU810 | Undescribed species | *A*. *pollinicola* sp. nov. |
|  | *Portulaca grandiflora* | SDBR-CMU860 | Undescribed species | *A. kwanphayaoense* sp. nov. |
|  | *Thunbergia erecta* | SDBR-CMU811 | Undescribed species | *A*. *phayaoense* sp. nov. |
|  |  | SDBR-CMU857 | Undescribed species | *A*. *phayaoense* sp. nov. |

* more than 99% sequence identity with the type strain of a described species in ITS region, less than 99% as “Undescribed species.
