# Supplementary material for: Unexpected diversity of flower-associated Aureobasidium reveals sixteen novel species from northern Thailand
Source: IMA Fungus. 2026 Aug 3;17:e188009. doi: 10.3897/imafungus.17.188009 (PMC13458542; doi:10.3897/imafungus.17.188009)
Supplement: Supplementary file 3 [file imafungus-17-e188009-s003.docx]

**Table S2.** Fungal name, strain numbers and corresponding GenBank accession numbers of the taxa used in the phylogenetic analyses. The type species are indicated in superscript “T”. The newly generated sequences are indicated in black bold.

| **Taxa** | **Strain numbers** | **GenBank Accession number** | | | | | **Reference** |
| --- | --- | --- | --- | --- | --- | --- | --- |
|  |  | **ITS** | **LSU** | ***rpb2*** | ***tef1*** | ***tub*** |  |
| *Aureobasidium aerium* | CFCC 50324^T^ | ON007058 | ON007081 | – | – | – | Wang et al. (2022) |
| *Aureobasidium aerium* | CFCC 56931 | ON007059 | ON007082 | – | – | – | Wang et al. (2022) |
| *Aureobasidium albui* | NRRL 61482^T^ | OK376936 | OK353650 | OK485130 | OK413253 | OK485072 | Jumbam et al. (2025) |
| *Aureobasidium albui* | SA57 | OK376939 | OK353651 | OM321986 | OK413254 | OK485073 | Jumbam et al. (2025) |
| ***Aureobasidium albui*** | **SDBR-CMU866** | **PX919960** | **PX920014** | **PZ022248** | **PZ022348** | **PZ022294** | **This study** |
| ***Aureobasidium anthicola*** | **SDBR-CMU821** | **PX919961** | **PX920015** | **PZ022249** | **PZ022349** | **PZ022295** | **This study** |
| ***Aureobasidium anthicola*** | **SDBR-CMU851^T^** | **PX919962** | **PX920016** | **PZ022250** | **PZ022350** | **PZ022296** | **This study** |
| *Aureobasidium aubasidani* | CBS 100524^T^ | FJ150905 | FJ150952 | – | FJ157900 | FJ157867 | Zalar et al. (2008) |
| *Aureobasidium aurantiacum* | BRIP 68300a^T^ | OM417256 | OM333570 | ON624213 | – | ON995119 | Steinrucken et al. (2022) |
| *Aureobasidium bupleuri* | CBS 131304^T^ | KT693738 | – | KT693981 | – | – | Haelewaters et al. (2021) |
| ***Aureobasidium bougainvilleae*** | **SDBR-CMU858^T^** | **PX919963** | **PX920017** | **PZ022251** | **PZ022351** | **PZ022297** | **This study** |
| ***Aureobasidium bougainvilleae*** | **SDBR-CMU859** | **PX919964** | **PX920018** | **PZ022252** | **PZ022352** | **PZ022298** | **This study** |
| *Aureobasidium castaneae* | CFCC 54591^T^ | MW364284 | MW364275 | – | – | – | Jiang et al. (2021) |
| ***Aureobasidium castaneae*** | **SDBR-CMU835** | **PX919965** | **PX920019** | **PZ022253** | **PZ022353** | **PZ022299** | **This study** |
| *Aureobasidium caatingaense* | URM 8594^T^ | OR340179 | OR340237 | – | PQ140158 | PP840908 | da Silva et al. (2024) |
| *Aureobasidium caatingaense* | URM 8596 | OR340180 | OR340238 | – | PQ140159 | PP840909 | da Silva et al. (2024) |
| ***Aureobasidium catharanthi*** | **SDBR-CMU812** | **PX919966** | **PX920020** | **PZ022254** | **PZ022354** | **PZ022300** | **This study** |
| ***Aureobasidium catharanthi*** | **SDBR-CMU842^T^** | **PX919967** | **PX920021** | **PZ022255** | **PZ022355** | **PZ022301** | **This study** |
| *Aureobasidium caulivorum* | CBS 242.64^T^ | FJ150871 | FJ150944 | KT693983 | – | – | Zalar et al. (2008) |
| *Aureobasidium cavalettoi* | INDP001 | OK376876 | – | – | – | – | Jumbam et al. (2025) |
| *Aureobasidium cavalettoi* | NRRL 64177^T^ | OK376877 | OK353610 | OK485112 | OK413225 | OK485046 | Jumbam et al. (2025) |
| ***Aureobasidium chiangmaiense*** | **SDBR-CMU818** | **PX919968** | **PX920022** | **PZ022256** | **PZ022356** | **PZ022302** | **This study** |
| ***Aureobasidium chiangmaiense*** | **SDBR-CMU819^T^** | **PX919969** | **PX920023** | **PZ022257** | **PZ022357** | **PZ022303** | **This study** |
| *Aureobasidium diazvalderramae* | MCA7156 | OK376906 | OK353631 | OK485122 | OK413238 | OK485060 | Jumbam et al. (2025) |
| *Aureobasidium diazvalderramae* | NRRL 64175^T^ | OK376904 | OK353629 | OK485121 | OK413237 | OK485059 | Jumbam et al. (2025) |
| *Aureobasidium ellingtonae* | NRRL 64185^T^ | OK376885 | OK353615 | OK485116 | OK413230 | OK485051 | Jumbam et al. (2025) |
| *Aureobasidium ellingtonae* | JB68 | OK376883 | OK353614 | OL505737 | OK413229 | OK485050 | Jumbam et al. (2025) |
| *Aureobasidium epipinicola* | CBS 152341^T^ | OR334647 | OR334848 | – | PV067689 | PV067682 | Czachura and Piątek (2025) |
| *Aureobasidium essambei* | NRRL 64172^T^ | OK376911 | OK353636 | OM321982 | OK413243 | OK485064 | Jumbam et al. (2025) |
| *Aureobasidium fermentans* | CBS 298.56^T^ | MH857648 | MH869192 | – | – | – | Vu et al. (2019) |
| *Aureobasidium faidherbiae* | CBS 149677^T^ | OQ628485 | OQ629067 | OQ627945 | OQ627952 | – | Crous et al. (2023) |
| *Aureobasidium faidherbiae* | SQU-KH09 | KU945836 | – | – | – | – | Crous et al. (2023) |
| ***Aureobasidium florale*** | **SDBR-CMU830** | **PX919970** | **PX920024** | **PZ022242** | **PZ022358** | **PZ022304** | **This study** |
| ***Aureobasidium florale*** | **SDBR-CMU831** | **PX919971** | **PX920025** | **PZ022243** | **PZ022359** | **PZ022305** | **This study** |
| ***Aureobasidium florale*** | **SDBR-CMU832^T^** | **PX919972** | **PX920026** | **PZ022244** | **PZ022360** | **PZ022306** | **This study** |
| ***Aureobasidium florale*** | **SDBR-CMU840** | **PX919973** | **PX920027** | **PZ022245** | **PZ022361** | **PZ022307** | **This study** |
| ***Aureobasidium florale*** | **SDBR-CMU850** | **PX919974** | **PX920028** | **PZ022246** | **PZ022362** | **PZ022308** | **This study** |
| ***Aureobasidium florale*** | **SDBR-CMU862** | **PX919975** | **PX920029** | **PZ022247** | **PZ022363** | **PZ022309** | **This study** |
| ***Aureobasidium floricola*** | **SDBR-CMU829^T^** | **PX919976** | **PX920030** | **PZ022258** | **PZ022364** | **PZ022310** | **This study** |
| ***Aureobasidium floricola*** | **SDBR-CMU853** | **PX919977** | **PX920031** | **PZ022259** | **PZ022365** | **PZ022311** | **This study** |
| ***Aureobasidium florigenum*** | **SDBR-CMU828** | **PX919978** | **PX920032** | **PZ022260** | **PZ022366** | **PZ022312** | **This study** |
| ***Aureobasidium florigenum*** | **SDBR-CMU865^T^** | **PX919979** | **PX920033** | **PZ022261** | **PZ022367** | **PZ022313** | **This study** |
| *Aureobasidium harposporum* | CBS 122914 | KT693741 | – | – | – | – | van Nieuwenhuijzen et al. (2016) |
| *Aureobasidium harposporum* | F121513^T^ | JN886794 | JN886794 | – | – | – | Bills et al. (2012) |
| *Aureobasidium insectorum* | KCL139^T^ | OP856707 | OP857208 | – | – | – | Wu et al. (2023) |
| *Aureobasidium insectorum* | LPL-1C | OP856705 | OP857207 | – | – | – | Wu et al. (2023) |
| *Aureobasidium insectorum* | XZY65-10 | OP856706 | OP857206 | – | – | – | Wu et al. (2023) |
| *Aureobasidium insectorum* | XZY249M1 | OP856713 | OP857214 | – | – | – | Wu et al. (2023) |
| *Aureobasidium intercalariosporum* | CGMCC 2.7208^T^ | OP856704 | OP857205 | – | – | – | Wu et al. (2023) |
| *Aureobasidium intercalariosporum* | MGL11-3 | OP856703 | OP857204 | – | – | – | Wu et al. (2023) |
| *Aureobasidium iranianum* | CCTU 268^T^ | KM093738 | KM093739 | – | – | – | Arzanlou and Khodaei (2012) |
| *Aureobasidium iranianum* | KH-P2 | KF758573 | KF758577 | – | KF758576 | KF758575 | da Silva et al. (2024) |
| *Aureobasidium iranianum* | QCC:M016/17 | KY781746 | KY781747 | – | – | – | da Silva et al. (2024) |
| *Aureobasidium khasianum* | NFCCI-4275^T^ | MH188305 | MH188306 | – | – | – | Prabhugaonkar and Pratibha (2018) |
| ***Aureobasidium kwanphayaoense*** | **SDBR-CMU839** | **PX919980** | **PX920034** | **PZ022262** | **PZ022368** | **PZ022314** | **This study** |
| ***Aureobasidium kwanphayaoense*** | **SDBR-CMU844** | **PX919981** | **PX920035** | **PZ022263** | **PZ022369** | **PZ022315** | **This study** |
| ***Aureobasidium kwanphayaoense*** | **SDBR-CMU849^T^** | **PX919982** | **PX920036** | **PZ022264** | **PZ022370** | **PZ022316** | **This study** |
| ***Aureobasidium kwanphayaoense*** | **SDBR-CMU860** | **PX919983** | **PX920037** | **PZ022265** | **PZ022371** | **PZ022317** | **This study** |
| ***Aureobasidium lannaense*** | **SDBR-CMU833** | **PX919984** | **PX920038** | **PZ022266** | **PZ022372** | **PZ022318** | **This study** |
| ***Aureobasidium lannaense*** | **SDBR-CMU863^T^** | **PX919985** | **PX920039** | **PZ022267** | **PZ022373** | **PZ022319** | **This study** |
| *Aureobasidium leucospermi* | CBS 130593^T^ | KT693727 | MH877257 | KT693970 | – | – | This study |
| *Aureobasidium leucospermi* | CPC 15081 | JN712487 | JN712553 | – | – | – | This study |
| *Aureobasidium lini* | CBS 125.21^T^ | FJ150897 | FJ150946 | – | FJ157908 | FJ157873 | Zalar et al. (2008) |
| *Aureobasidium mangrovei* | IBRC:M 30265^T^ | NR_174637 | NG_078639 | – | – | – | Nasr et al. (2018) |
| *Aureobasidium mangrovei* | IBRC:M 30266 | KY089087 | KY089086 | – | – | – | Nasr et al. (2018) |
| *Aureobasidium mahoniae* | CBS 388.92^T^ | KT693746 | EU754213 | KT693987 | FJ157915 | FJ157874 | Zalar et al. (2008) |
| *Aureobasidium melanogenum* | 5JB | OR340285 | OR340188 | – | PQ140143 | PP840900 | da Silva et al. (2024) |
| *Aureobasidium melanogenum* | CBS 105.22^T^ | FJ150886 | FJ150926 | KT693972 | FJ157887 | FJ157858 | Zalar et al. (2008) |
| *Aureobasidium melanogenum* | CBS 621.80 | FJ150885 | FJ150921 | – | FJ157890 | FJ157859 | Zalar et al. (2008) |
| *Aureobasidium melanogenum* | CDH2020-10 | MT863788 | MT863787 | – | – | – | Lee et al. (2021) |
| *Aureobasidium melanogenum* | EXF-924 | FJ150883 | FJ150918 | – | FJ157885 | FJ157850 | Lee et al. (2021) |
| *Aureobasidium melanogenum* | FCCUFG 29 | OR340282 | OR340185 | – | PQ140147 | PP840905 | da Silva et al. (2024) |
| ***Aureobasidium melanogenum*** | **SDBR-CMU817** | **PX919986** | **PX920040** | **PZ022268** | **PZ022374** | **PZ022320** | **This study** |
| ***Aureobasidium melanogenum*** | **SDBR-CMU855** | **PX919987** | **PX920041** | **PZ022269** | **PZ022375** | **PZ022321** | **This study** |
| *Aureobasidium microtermitis* | GTS2.7^T^ | MW276135 | MW276136 | – | – | – | Crous et al. (2021) |
| *Aureobasidium microtermitis* | SRA1.2 | MW692853 | MW031809 | – | – | – | Crous et al. (2021) |
| *Aureobasidium microstictum* | CBS 342.66 | FJ150903 | FJ150945 | KT693985 |  | FJ157872 | Zalar et al. (2008) |
| *Aureobasidium motuoense* | E31-1 | OP856709 | OP857210 | – | – | – | Wu et al. (2023) |
| *Aureobasidium motuoense* | E26-4 | OP856708 | OP857209 | – | – | – | Wu et al. (2023) |
| *Aureobasidium motuoense* | E82-2 | OP856702 | OP857203 | – | – | – | Wu et al. (2023) |
| *Aureobasidium motuoense* | XZY411-4^T^ | OP856710 | OP857211 | – | – | – | Wu et al. (2023) |
| *Aureobasidium musti* | MCA7640 | OK376909 | OK353634 | – | OK413241 | OK485063 | Jumbam et al. (2025) |
| *Aureobasidium musti* | MCA7652 | OK376910 | OK353635 | OM321981 | OK413242 | – | Jumbam et al. (2025) |
| *Aureobasidium musti* | NRRL 64173^T^ | OK376993 | OK353658 | – | OK413260 | OK485076 | Jumbam et al. (2025) |
| ***Aureobasidium musti*** | **SDBR-CMU825** | **PX919988** | **PX920042** | **PZ022270** | **PZ022376** | **PZ022322** | **This study** |
| ***Aureobasidium musti*** | **SDBR-CMU864** | **PX919989** | **PX920043** | **PZ022271** | **PZ022377** | **PZ022323** | **This study** |
| *Aureobasidium namibiae* | CBS 147.97^T^ | FJ150875 | FJ150937 | KT693973 | – | FJ157863 | Zalar et al. (2008) |
| ***Aureobasidium oroxyli*** | **SDBR-CMU822** | **PX919990** | **PX920044** | **PZ022240** | **PZ022378** | **PZ022324** | **This study** |
| ***Aureobasidium oroxyli*** | **SDBR-CMU827^T^** | **PX919991** | **PX920045** | **PZ022241** | **PZ022379** | **PZ022325** | **This study** |
| *Aureobasidium peruvianum* | NRRL 64183^T^ | OK376905 | OK353630 | OM321980 | OL505731 | – | Jumbam et al. (2025) |
| ***Aureobasidium phayaoense*** | **SDBR-CMU811** | **PX919992** | **PX920046** | **PZ022272** | **PZ022380** | **PZ022326** | **This study** |
| ***Aureobasidium phayaoense*** | **SDBR-CMU852^T^** | **PX919993** | **PX920047** | **PZ022273** | **PZ022381** | **PZ022327** | **This study** |
| ***Aureobasidium phayaoense*** | **SDBR-CMU857** | **PX919994** | **PX920048** | **PZ022274** | **PZ022382** | **PZ022328** | **This study** |
| *Aureobasidium pini* | CFCC 52778^T^ | MK184533 | MK184535 | – | – | – | Jiang et al. (2019) |
| *Aureobasidium pini* | JN035 | MK184534 | MK184536 | – | – | – | Jiang et al. (2019) |
| *Aureobasidium planticola* | CGMCC2.7199 ^T^ | OP856711 | OP857212 | – | – | – | Wu et al. (2023) |
| *Aureobasidium planticola* | FCCUFG 30 | OR340291 | OR340194 | – | – | – | da Silva et al. (2024) |
| ***Aureobasidium plumeriae*** | **SDBR-CMU826** | **PX919995** | **PX920049** | **PZ022275** | **PZ022383** | **PZ022329** | **This study** |
| ***Aureobasidium plumeriae*** | **SDBR-CMU836^T^** | **PX919996** | **PX920050** | **PZ022276** | **PZ022384** | **PZ022330** | **This study** |
| ***Aureobasidium pollinicola*** | **SDBR-CMU810** | **PX919997** | **PX920051** | **PZ022277** | **PZ022385** | **PZ022331** | **This study** |
| ***Aureobasidium pollinicola*** | **SDBR-CMU813** | **PX919998** | **PX920052** | **PZ022278** | **PZ022386** | **PZ022332** | **This study** |
| ***Aureobasidium pollinicola*** | **SDBR-CMU843** | **PX919999** | **PX920053** | **PZ022279** | **PZ022387** | **PZ022333** | **This study** |
| ***Aureobasidium pollinicola*** | **SDBR-CMU861^T^** | **PX920000** | **PX920054** | **PZ022280** | **PZ022388** | **PZ022334** | **This study** |
| *Aureobasidium proteae* | CBS 114273 | JN712491 | JN712557 | KT693974 | – | – | Crous et al. (2011) |
| *Aureobasidium proteae* | CPC 13701 | JN712490 | JN712556 | – | – | – | Crous et al. (2011) |
| *Aureobasidium proteae* | CBS 111970^T^ | JN712493 | JN712559 | – | – | – | Crous et al. (2011) |
| *Aureobasidium pullulans* | CBS 584.75^T^ | FJ150906 | FJ150942 | KT693976 | FJ157895 | FJ157869 | Zalar et al. (2008) |
| *Aureobasidium pullulans* | CBS 701.76 | FJ150907 | FJ150951 | – | – | FJ157865 | Zalar et al. (2008) |
| *Aureobasidium pullulans* | CBS 146.30 | FJ150902 | FJ150916 | – | – | FJ157871 | Zalar et al. (2008) |
| *Aureobasidium pullulans* | EXF-150 | OX346314 | – | OX346389 | – | – | Zalar et al. (2008) |
| *Aureobasidium pullulans* | CBS 100280 | FJ150910 | FJ150941 | KT693977 | FJ157906 | FJ157864 | Zalar et al. (2008) |
| *Aureobasidium rubi* | INDP43 | OK376878 | OK353611 | OK485113 | OK413226 | OK485047 | Jumbam et al. (2025) |
| *Aureobasidium rubi* | NRRL 64179^T^ | OK376892 | OK353620 | OM321979 | OK413232 | OK485054 | Jumbam et al. (2025) |
| ***Aureobasidium saisamorniae*** | **SDBR-CMU846** | **PX920001** | **PX920055** | **PZ022281** | **PZ022389** | **PZ022335** | **This study** |
| ***Aureobasidium saisamorniae*** | **SDBR-CMU848^T^** | **PX920002** | **PX920056** | **PZ022282** | **PZ022390** | **PZ022336** | **This study** |
| ***Aureobasidium saisamorniae*** | **SDBR-CMU854** | **PX920003** | **PX920057** | **PZ022283** | **PZ022391** | **PZ022337** | **This study** |
| *Aureobasidium santateresinhaense* | URM 8593^T^ | OR340177 | OR340235 | – | PQ140157 | PP840910 | da Silva et al. (2024) |
| *Aureobasidium santateresinhaense* | URM 8595 | OR340178 | OR340236 | – | PQ140156 | PP840911 | da Silva et al. (2024) |
| ***Aureobasidium savitreeae*** | **SDBR-CMU824^T^** | **PX920004** | **PX920058** | **PZ022284** | **PZ022392** | **PZ022338** | **This study** |
| ***Aureobasidium savitreeae*** | **SDBR-CMU841** | **PX920005** | **PX920059** | **PZ022285** | **PZ022393** | **PZ022339** | **This study** |
| ***Aureobasidium siamense*** | **SDBR-CMU836** | **PX920006** | **PX920060** | **PZ022286** | **PZ022394** | **PZ022340** | **This study** |
| ***Aureobasidium siamense*** | **SDBR-CMU847^T^** | **PX920007** | **PX920061** | **PZ022287** | **PZ022395** | **PZ022341** | **This study** |
| *Aureobasidium subglaciale* | EXF 2481^T^ | FJ150895 | FJ150913 | – | FJ157911 | FJ157878 | Zalar et al. (2008) |
| *Aureobasidium subglaciale* | EXF 3640 | FJ150896 | FJ150939 | – | FJ157913 | FJ157882 | Zalar et al. (2008) |
| *Aureobasidium subglaciale* | EXF-2491 | FJ150894 | FJ150936 | – | FJ157902 | FJ157880 | Zalar et al. (2008) |
| *Aureobasidium toomeae* | NRRL 64186^T^ | OK376912 | OK353638 | OK485125 | OL505732 | OK485065 | Jumbam et al. (2025) |
| *Aureobasidium tremulum* | ATS4.1 | ON954746 | OK500010 | – | – | – | Crous et al. (2019) |
| *Aureobasidium tremulum* | ATS4.2 | ON954747 | OK500011 | – | – | – |  |
| ***Aureobasidium tremulum*** | **SDBR-CMU814** | **PX920008** | **PX920062** | **PZ022288** | **PZ022396** | **PZ022342** | **This study** |
| ***Aureobasidium tremulum*** | **SDBR-CMU816** | **PX920009** | **PX920063** | **PZ022289** | **PZ022397** | **PZ022343** | **This study** |
| ***Aureobasidium tremulum*** | **SDBR-CMU820** | **PX920010** | **PX920064** | **PZ022290** | **PZ022398** | **PZ022344** | **This study** |
| ***Aureobasidium tremulum*** | **SDBR-CMU834** | **PX920011** | **PX920065** | **PZ022291** | **PZ022399** | **PZ022345** | **This study** |
| ***Aureobasidium tremulum*** | **SDBR-CMU838** | **PX920012** | **PX920066** | **PZ022292** | **PZ022400** | **PZ022346** | **This study** |
| ***Aureobasidium tremulum*** | **SDBR-CMU856** | **PX920013** | **PX920067** | **PZ022293** | **PZ022401** | **PZ022347** | **This study** |
| *Aureobasidium tremulum* | UN 1^T^ | MK503657 | MK503660 | – | – | – | Wu et al. (2023) |
| *Aureobasidium thailandense* | NRRL 58539^T^ | JX462674 | JX462674 | – | EU719352 | EU719407 | Peterson et al. (2013) |
| *Aureobasidium thailandense* | NRRL 58543 | JX462675 | JX462675 | – | – | – | Peterson et al. (2013) |
| *Aureobasidium uvarum* | AWRI4619 | CAIJEN010000012 | | – | – | – | Onetto et al. (2020) |
| *Aureobasidium uvarum* | AWRI4620^T^ | CAINUL010000013 | | – | – | – | Onetto et al. (2020) |
| *Aureobasidium vanuatuense* | NRRL 64180^T^ | OK376908 | OK353633 | OK485124 | OK413240 | OK485062 | Jumbam et al. (2025) |
| *Aureobasidium welwitschiae* | CBS 149676^T^ | OQ628484 | OQ629066 | – | OQ627959 | OQ627965 | Crous et al. (2023) |
| *Aureobasidium xishuangbannaense* | KUMCC21-0702 | ON426834 | OP363257 | – | – | – | Hyde et al. (2023) |
| *Aureobasidium xishuangbannaense* | KUMCC21-0703^T^ | ON426835 | OP363258 | – | – | – | Hyde et al. (2023) |
| *Aureobasidium* sp. (as *A. microstictum*) | CBS 114.64 | FJ150873 | FJ150940 | KT693986 | FJ157914 | – | Zalar et al. (2008) |
| *Aureobasidium* sp. | SAM12 | MN880467 | MN887589 | – | – | – | da Silva et al. (2024) |
| *Aureobasidium* sp. | C802 | KY931496 | – | – | – | – | da Silva et al. (2024) |
| *Aureobasidium* sp. 2 (as *A. zeae*) | A.z-1 | KP226696 | – | – | – | – | Jumbam et al. (2025) |
| *Aureobasidium* sp. 2 (as *A. zeae*) | TJ-3 | KP067091 | – | – | – | – | Jumbam et al. (2025) |
| *Delphinella strobiligena* | CBS 735.71 | MH860318 | MH872074 | – | – | – | Vu et al. (2019) |
| *Sydowia polyspora* | CBS:116.29 | MH855019 | MH866487 | GU357791 | – | – | Vu et al. (2019) |

**Reference** (The references in the table are separated from those in the main manuscript)

Arzanlou M, Khodaei S (2012) *Aureobasidium iranianum*, a new species on bamboo from Iran. Mycosphere 3: 404–408. https://doi.org/10.5943/mycosphere/3/4/2

Bills GF, Menéndez VG, Platas G (2012) *Kabatiella bupleuri* sp. nov. (*Dothideales*), a pleomorphic epiphyte and endophyte of the Mediterranean plant *Bupleurum gibraltarium* (Apiaceae). Mycologia 104: 962–973. https://doi.org/10.3852/12-003

Crous PW, Osieck ER, Jurjevi Ž et al. (2021) Fungal Planet description sheets: 1284–1382. Persoonia 47: 178–374. https://doi.org/10.3767/PERSOONIA.2021.47.06

Crous PW, Osieck ER, Shivas RG et al. (2023) Fungal Planet description sheets: 1478–1549. Persoonia-Molecular Phylogeny and Evolution of Fungi 50: 158–310. https://doi.org/10.3767/persoonia.2023.50.05

Crous PW, Summerell Ba, Swart L et al. (2011) Fungal pathogens of *Proteaceae*. Persoonia 27: 20–45. https://doi.org/10.3767/003158511X606239

Czachura P, Piątek M (2025) The genus *Aureobasidium* from sooty mould communities in Poland, including *A. epipinicola* sp. nov. and *A. insectorum*, new to Europe. Plant Pathology 74: 1593–1611. https://doi.org/10.1111/ppa.14109

da Silva AC, Ferro LO, Alves VC et al. (2024) Phylogenetic evidence revealed new *Aureobasidium* species (*Saccotheciaceae*, *Dothideales*) from Brazil. Mycological Progress 23: 70. https://doi.org/10.1007/s11557-024-02009-4

Haelewaters D, Urbina H, Brown S et al. (2021) Isolation and molecular characterization of the romaine lettuce phylloplane mycobiome. Journal of Fungi 7: e277. https://doi.org/10.3390/jof7040277

Hyde KD, Norphanphoun C, Ma J et al. (2023) Mycosphere notes 387–412-novel species of fungal taxa from around the world. Mycosphere 14: 663–744 https://doi.org/10.5943/mycosphere/14/1/8

Jumbam B, Zasada IA, Bensch K et al. (2025) A taxonomic revision of *Aureobasidium* (*Saccotheciaceae*, *Dothideales*) with new species, new names, and typifications. Persoonia 54: 147–195. https://doi.org/10.3114/persoonia.2025.54.05

Jiang N, Fan X, Tian C (2021) Identification and characterization of leaf-inhabiting fungi from *Castanea* plantations in China. Journal of Fungi 7: e64. https://doi.org/10.3390/ jof7010064

Jiang N, Liang YM, Tian CM (2019) *Aureobasidium pini* sp. nov. from pine needle in China. Phytotaxa 402: 199–206. https://doi.org/10.11646/PHYTOTAXA.402.4.3

Lee DH, Cho SE, Oh JY et al. (2021) A novel species of *Aureobasidium* (*Dothioraceae*) recovered from *Acer pseudosieboldianum* in Korea. Journal of Asia-Pacific Biodiversity 14: 657–661. https://doi.org/10.1016/j.japb.2021.08.005

Nasr S, Mohammadimehr M, Geranpayeh Vaghei M et al. (2018) *Aureobasidium mangrovei* sp. nov., an ascomycetous species recovered from Hara protected forests in the Persian Gulf, Iran. Antonie van Leeuwenhoek 111: 1697–1705. https://doi.org/10.1007/ s10482- 018-1059-z

Onetto CA, Schmidt SA, Roach MJ et al. (2020) Comparative genome analysis proposes three new *Aureobasidium* species isolated from grape juice. FEMS Yeast Research 20: foaa052. https://doi.org/10.1093/femsyr/foaa052

Peterson SW, Manitchotpisit P, Leathers TD (2013) *Aureobasidium thailandense* sp. nov. isolated from leaves and wooden surfaces. International Journal of Systematic and Evolutionary Microbiology, 63: 790–795. https://doi.org/10.1099/ijs.0.047613-0

Prabhugaonkar A, Pratibha J (2018) *Aureobasidium khasianum* (*Aureobasidiaceae*) a novel species with distinct morphology. Phytotaxa 374: 257–262. https://doi.org/10.11646/phytotaxa. 374.3.7

Steinrucken TV, Vitelli JS, Holdom DG et al. (2022) The diversity of microfungi associated with grasses in the *Sporobolus indicus* complex in Queensland, Australia. Frontiers in Fungal Biology 3: 956837. https://doi.org/10.3389/ffunb.2022.956837

van Nieuwenhuijzen EJ, Houbraken JAMP, Meijer M et al. (2016) *Aureobasidium melanogenum*: a native of dark biofinishes on oil treated wood. Antonie van Leeuwenhoek 109(5): 661–683. https://doi.org/10.1007/S10482-016-0668-7

Vu D, Groenewald M, de Vries M et al. (2019) Large-scale boosts coverage for kingdom fungi and reveals thresholds for fungal species and higher taxon delimitation. Studies in mycology 92(1): 135–154. https://doi.org/10.1016/J.SIMYCO.2018.05.001

Wang C-B, Jiang N, Tu Y et al. (2022b) *Aureobasidium aerium* (*Saccotheciaceae*, *Dothideales*), a new yeast-like fungus from the air in Beijing. China. Phytotaxa 544: 185–192. https://doi.org/10.11646/PHYTOTAXA.544.2.4

Wu F, Feng Z, Wang M et al. (2023) Proposal of four new *Aureobasidium* species for exopolysaccharide production. Journal of Fungi 9: 447. https://doi.org/10.3390/jof9040447

Zalar P, Gostinčar C, De Hoog GS et al. (2008) Redefinition of *Aureobasidium pullulans* and its varieties. Studies in Mycology 61: 21–38. https://doi.org/10.3114/sim.2008.61.02
